# In Situ Bioorthogonal Synthesis of PROTACs via Dual‐Responsive Cleavage for Synergistic Photo‐Immunotherapy

**DOI:** 10.1002/advs.77938

**Published:** 2026-09-27

**Authors:** Shiqin Jian, Jiasha Wu, Fusheng Xu, Yan Zuo, Huaxing Shen, Rui Ji, Luyi Wang, Na Li, Yanting Sun, Yongsheng Yu, Yejiao Shi, Honggang Hu, Feng Xu, Dan Huang, Xiaochun Hu

**Affiliations:** ^1^ School of Medicine Department of Chemistry College of Sciences Shanghai University Shanghai China; ^2^ Department of Medical Oncology Cancer Center Shanghai General Hospital Shanghai Jiao Tong University School of Medicine Shanghai China; ^3^ Institute of Translational Medicine Shanghai University Shanghai China; ^4^ Institute of Translational Medicine Shanghai Jiao Tong University Shanghai China; ^5^ School of Pharmacy Chengdu Medical College Chengdu China; ^6^ Department of Neurosurgery Huashan Hospital Shanghai Medical College Fudan University Shanghai China; ^7^ Department of Pathology Fudan University Shanghai Cancer Center Shanghai China; ^8^ Shanghai Integration and Innovation Center of Marine Medical Engineering Shanghai University Shanghai China

**Keywords:** dual‐responsive cleavage, in situ bioorthogonal synthesis, photo‐immunotherapy, proteolysis‐targeting chimera

## Abstract

Limited aqueous dispersibility and potential off‐target toxicity hinder the development and clinical translation of proteolysis‐targeting chimeras (PROTACs). Herein, a bioactive self‐delivering “Split Nano‐Assembly of Photosensitizers and Targeting Chimeras” (SNAP‐TAC) theranostic platform is developed to address these challenges and enable tumor‐selective synergistic photo‐immunotherapy. To ensure synchronized in vivo delivery, an intact BRD4 degrader is chemically split into a hydrophobic targeting precursor and an amphiphilic photosensitizer‐conjugated peptide, which spontaneously co‐assemble into discrete nanoparticles. Within the tumor microenvironment (TME), elevated cathepsin B and glutathione trigger dual‐responsive peptide cleavage and disulfide reduction. This unloads the bulky photosensitizer and exposes the reactive 1,2‐aminothiol motifs, driving the in situ bioorthogonal synthesis of the active PROTAC via metal‐free CBT‐Cys click condensation. Consequently, active degraders are preferentially generated in tumor‐associated environments. Additionally, the intrinsic fluorescence of the photosensitizer enables real‐time fluorescence tracking in vivo to guide localized therapy. Therapeutically, upon localized irradiation, the released photosensitizer induces immunogenic cell death, which synergizes with BRD4 depletion‐mediated PD‐L1 downregulation and immune‐pathway modulation to activate anti‐tumor immunity. In vivo evaluations demonstrate that this synergy effectively remodels the suppressive “cold” TME into a cytolytic “hot” phenotype. Ultimately, this chemical biology approach effectively addresses the intrinsic selectivity and delivery limitations of conventional PROTACs.

## Graphical abstract

A bioactive self‐delivering SNAP‐TAC platform responds to tumor‐associated cathepsin B and glutathione, enabling in situ bioorthogonal synthesis of a BRD4‐degrading proteolysis‐targeting chimera within the tumor microenvironment. The resulting BRD4 degradation and PD‐L1 downregulation synergize with photodynamic therapy‐induced immunogenic cell death, converting immunosuppressive ‘cold’ tumors into immune‐active ‘hot’ tumors and enhancing photo‐immunotherapy.

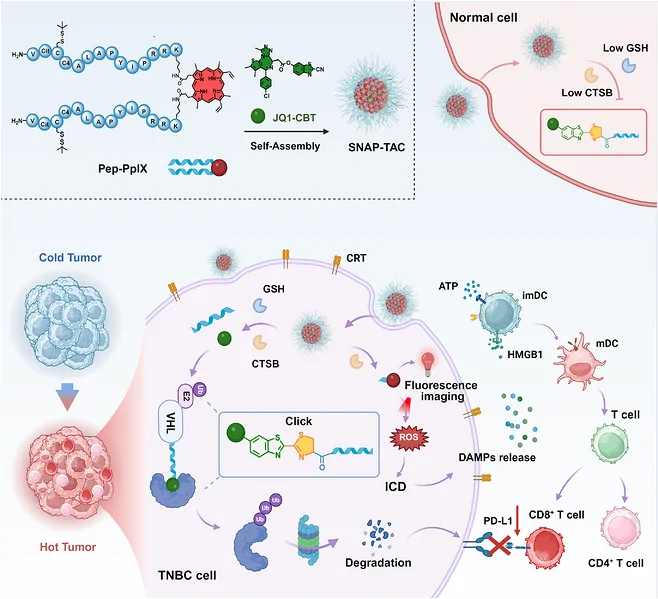

## 1 Introduction

Proteolysis‐targeting chimeras (PROTACs) achieve “event‐driven” degradation of pathogenic proteins by hijacking the endogenous ubiquitin‐proteasome system, providing a robust approach against traditionally undruggable targets [1, 2]. However, the requirement for simultaneous binding to both the target protein and the E3 ligase results in a relatively bulky and often hydrophobic molecular architecture. This inherent structural property leads to poor aqueous dispersibility, which significantly limits their in vivo delivery and overall efficacy [3, 4]. More critically, the systemic distribution of these constitutively active degraders lacks tumor selectivity, potentially resulting in severe off‐target toxicity in healthy tissues [5, 6]. Therefore, addressing the delivery barrier and achieving highly selective, tumor‐specific protein degradation remain formidable challenges for the clinical translation of PROTACs.

To address the dual challenges of poor dispersibility and systemic toxicity, encapsulating intact PROTACs into exogenous nanocarriers has been widely explored and has made significant contributions to the field [7]. However, these physical delivery systems can face potential limitations, including the premature leakage of active therapeutics during systemic circulation, limited drug‐loading capacity, and clearance‐related concerns associated with exogenous carrier materials [8, 9, 10]. To bypass these systemic delivery issues and achieve on‐demand activation, recent stimuli‐responsive nanoplatforms have elegantly utilized masked PROTAC prodrugs to enable tumor‐activated targeted protein degradation and photo‐immunotherapy [11, 12]. Complementary to the delivery of these caged intact PROTACs, an integrated chemical strategy—splitting the bulky PROTAC into inactive precursors for in situ bioorthogonal synthesis within the tumor microenvironment (TME)—has emerged as a chemically elegant alternative. This strategy improves tumor selectivity by restricting the generation of the active degrader to the target site. Currently, several established bioorthogonal toolkits are widely utilized for in vivo generation, such as copper‐catalyzed azide‐alkyne cycloaddition (CuAAC) [13], strain‐promoted azide‐alkyne cycloaddition (SPAAC, e.g., DBCO‐N_3_) [14, 15], and inverse electron‐demand Diels‐Alder (IEDDA) reactions [16]. Among these, the 2‐cyanobenzothiazole (CBT) and cysteine (Cys) click condensation stands out as a particularly attractive pathway for PROTAC synthesis. Inspired by the natural bioluminescence mechanism of fireflies, this highly biocompatible reaction proceeds rapidly under physiological conditions without the need for transition metal catalysts [17]. Importantly, both the CBT and 1,2‐aminothiol motifs are sterically compact, making them ideally suited for modifying precursor modules without introducing significant steric hindrance, thus presenting substantial translational prospects for safe and precise in situ drug generation [18, 19].

Despite the chemical elegance of the in situ CBT‐Cys reaction, a critical in vivo bottleneck remains, as administering the two clickable precursors in their free forms often leads to divergent pharmacokinetic profiles and unequal tumor accumulation due to their distinct physicochemical properties [20]. This pharmacokinetic disparity compromises the efficiency of in situ condensation, as the precursors cannot reach the tumor site synchronously [13, 21, 22]. While utilizing exogenous vehicles to co‐deliver these precursors could resolve the synchronization issue, it may reintroduce the aforementioned liabilities associated with inert carrier matrices. To achieve synchronized delivery while avoiding reliance on additional non‐functional exogenous carriers, bioactive self‐delivery offers a promising strategy. By rationally engineering one precursor as an amphiphilic, multifunctional, TME‐responsive peptide and pairing it with a hydrophobic small‐molecule effector, the components spontaneously co‐assemble into discrete nanoparticles driven by non‐covalent interactions [23]. This design integrates structural support, delivery, and therapeutic functions, facilitating the colocalized and synchronized accumulation of both precursors. Upon reaching the TME, specific physiological stimuli trigger peptide cleavage, disassembling the nanostructure and exposing the reactive motifs for in situ click condensation.

**SCHEME 1:**
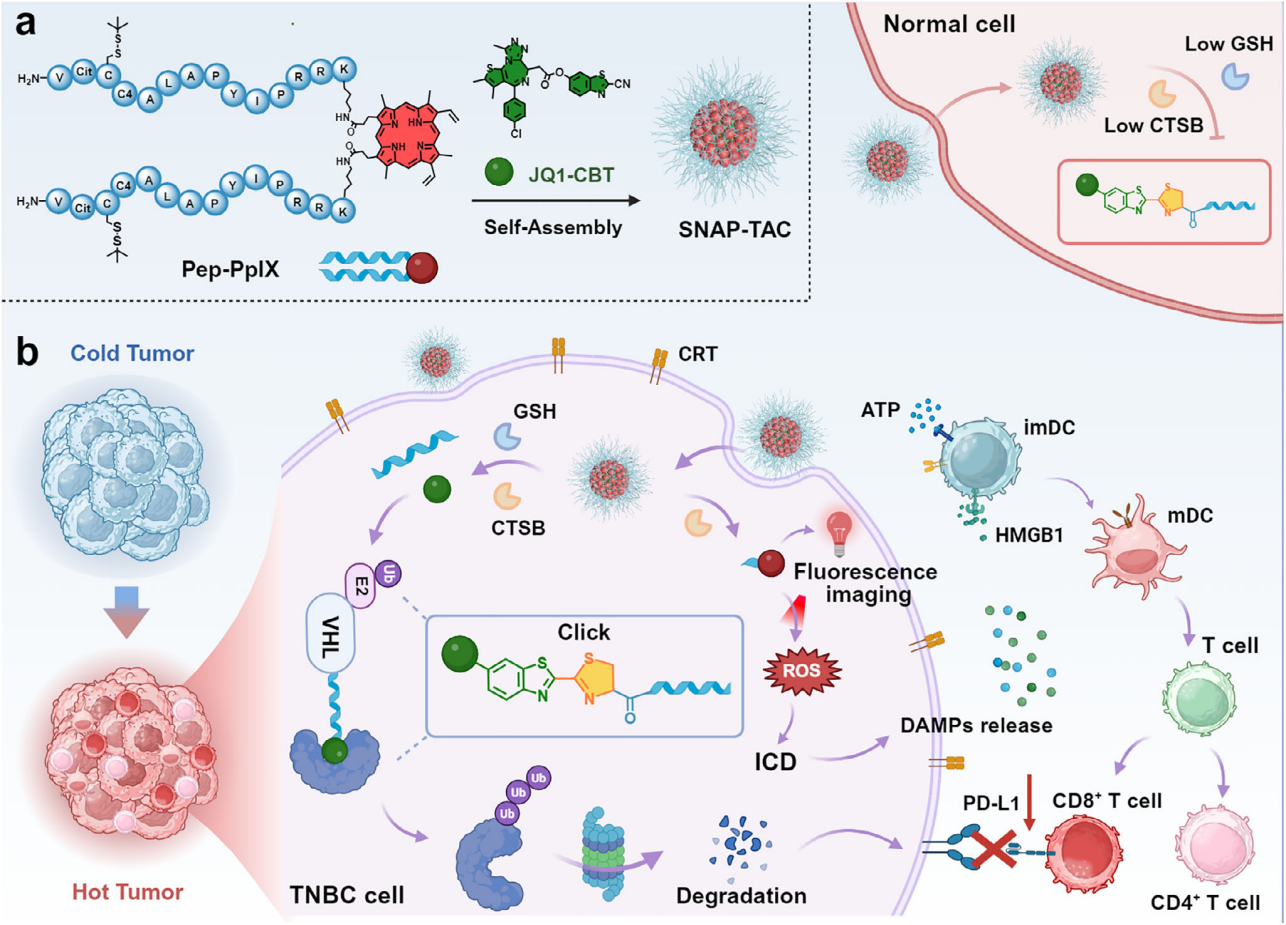
Schematic illustration of the bioactive self‐delivering SNAP‐TAC platform for synergistic photo‐immunotherapy. (a) Spontaneous co‐assembly process of JQ1‐CBT and Pep‐PpIX into SNAP‐TAC nanoparticles. (b) In vivo working mechanisms. In normal cells with basal levels of CTSB and GSH, SNAP‐TAC maintains relative stability, which restricts the generation of the active PROTAC and reduces potential off‐target toxicity. Conversely, in the tumor microenvironment characterized by elevated CTSB and GSH, SNAP‐TAC undergoes efficient dual‐responsive cleavage to trigger the in situ bioorthogonal synthesis of the BRD4‐PROTAC. Concurrently, the intrinsic fluorescence of the photosensitizer enables real‐time imaging to guide precise localized therapy. Under 660 nm irradiation, the in situ generated PROTAC mediates BRD4 degradation and subsequent PD‐L1 downregulation, which synergizes with PDT‐induced ICD, promoting the transition of immunosuppressive "cold" tumors into cytolytic "hot" tumors to activate systemic anti‐tumor immunity.

To validate the concept of bioactive self‐delivery coupled with in situ CBT‐Cys PROTAC synthesis, we developed the “Split Nano‐Assembly of Photosensitizers and Targeting Chimeras” (SNAP‐TAC) strategy (Scheme 1). In this rational design, a BRD4 small‐molecule inhibitor modified with a CBT motif (JQ1‐CBT) serves as the hydrophobic precursor. Concurrently, an engineered conjugate—[(VCitC(StBu)‐C_4_‐ALAPYIP‐RRK)_2_‐PpIX]—integrating a VHL‐binding peptide, a cathepsin B (CTSB)‐cleavable linker, and a photosensitizer (protoporphyrin IX, PpIX) acts as the multifunctional amphiphilic assembly unit. Driven by non‐covalent interactions, these components efficiently encapsulate the hydrophobic JQ1‐CBT within their core, forming the stable SNAP‐TAC nanoparticles. Validations confirmed that within the tumor microenvironment, SNAP‐TAC undergoes precise dual‐responsive cleavage to synthesize the active BRD4‐PROTAC in situ, whereas SNAP‐TAC‐induced BRD4 degradation was limited in normal NIH‐3T3 fibroblasts, which may help mitigate potential off‐target toxicity. Furthermore, evaluations in tumor‐bearing mice demonstrated that SNAP‐TAC effectively accumulates at the tumor region, where the intrinsic fluorescence of PpIX provides real‐time imaging to guide precise localized irradiation. Upon localized 660 nm irradiation, this system triggers a synergistic mechanism that inhibits tumor growth: the in situ‐generated PROTAC mediates BRD4 degradation to transcriptionally reprogram the TME and downregulate PD‐L1, thereby complementing photodynamic therapy (PDT)‐induced immunogenic cell death (ICD) [24, 25, 26, 27]. Consequently, this synergy remodels the immunosuppressive “cold” tumor into a cytolytic “hot” phenotype. This strategy addresses the selectivity and delivery limitations of conventional PROTACs through synergistic photo‐immunotherapy.

## 2 Results and Discussion

### 2.1 Synthesis, Preparation, and Characterization of SNAP‐TAC

To enable TME‐responsive synergistic photo‐immunotherapy, we first designed and synthesized the two core modules of the SNAP‐TAC system: the targeting precursor (JQ1‐CBT) and the stimuli‐responsive peptide‐photosensitizer conjugate (Pep‐PpIX). Specifically, JQ1‐CBT was synthesized from tBu‐JQ1 via TFA‐mediated deprotection followed by EDC/DMAP‐catalyzed esterification. The amphiphilic Pep‐PpIX was prepared using standard solid‐phase peptide synthesis (SPPS). The chemical structures and purities of all small‐molecule intermediates and the JQ1‐CBT were systematically confirmed by ^1^H‐NMR, ^13^C‐NMR, HRMS, and HPLC. Concurrently, the exact mass and purity of Pep‐PpIX were validated by HRMS and HPLC (Figures S1–S9).

**FIGURE 1:**
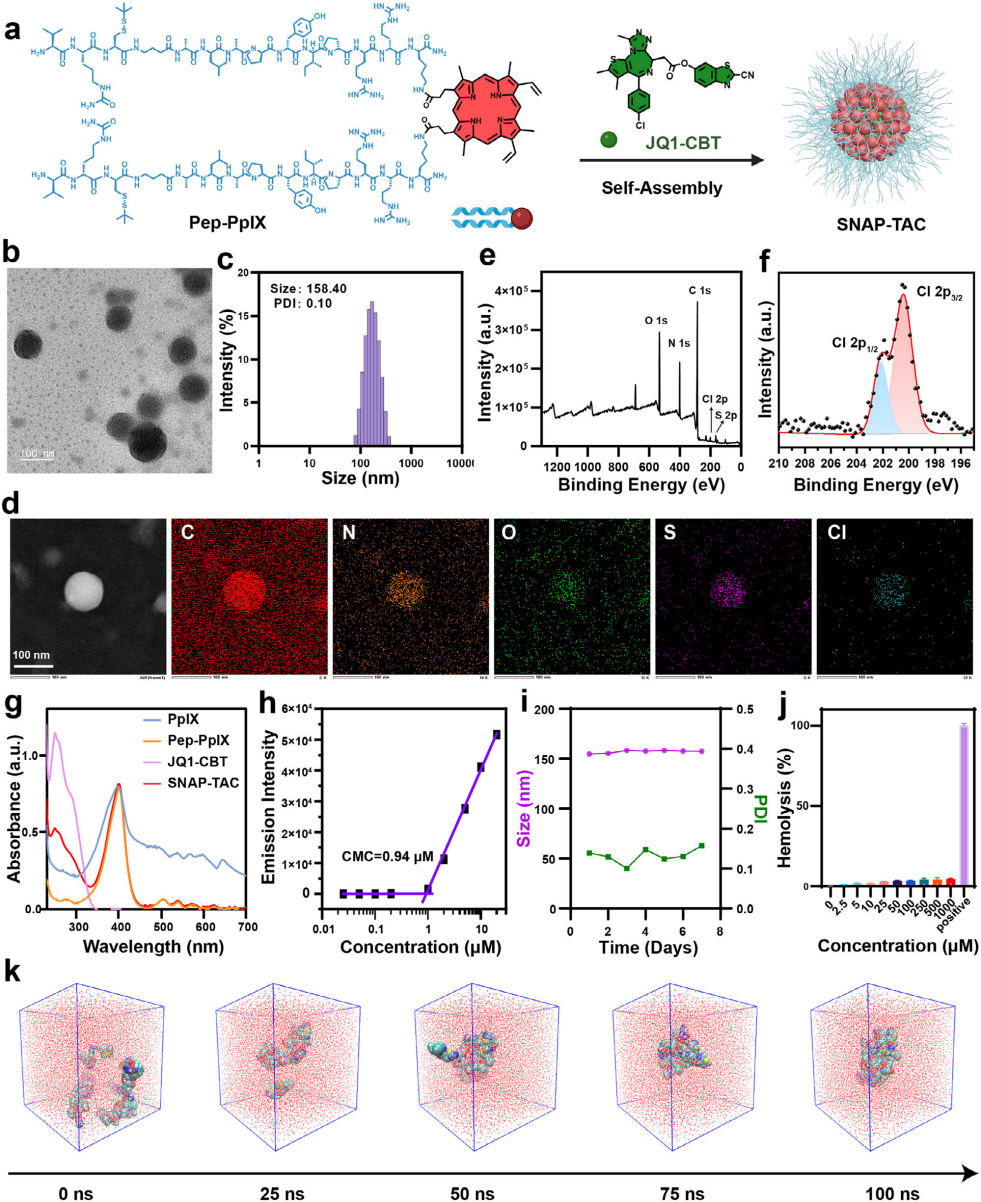
Preparation and physicochemical characterization of bioactive self‐delivering SNAP‐TAC nanoparticles. (a) Schematic illustration of the spontaneous co‐assembly process of Pep‐PpIX and JQ1‐CBT into SNAP‐TAC. (b) Representative transmission electron microscopy (TEM) image of SNAP‐TAC. Scale bar: 100 nm. (c) Hydrodynamic size distribution of SNAP‐TAC measured by dynamic light scattering (DLS). (d) HAADF‐STEM image and corresponding EDS elemental mapping (C, N, O, S, Cl) of an individual SNAP‐TAC nanoparticle. Scale bars = 100 nm. (e) X‐ray photoelectron spectroscopy (XPS) survey spectrum of SNAP‐TAC. (f) High‐resolution XPS spectrum of the Cl 2p peak. (g) UV–vis absorption spectra of free PpIX, Pep‐PpIX, JQ1‐CBT, and SNAP‐TAC. (h) Determination of the critical micelle concentration (CMC) of SNAP‐TAC. (i) Colloidal stability (hydrodynamic size and PDI) of SNAP‐TAC monitored over 7 days. (j) In vitro hemolysis assay of SNAP‐TAC at various concentrations. Data in (j) are presented as mean ± SD (*n* = 3). (k) Representative snapshots from all‐atom molecular dynamics (MD) simulations illustrating the spontaneous co‐assembly of Pep‐PpIX and JQ1‐CBT into a compact nanocluster over a 100 ns trajectory.

To reduce reliance on additional inert, non‐functional exogenous carrier matrices, we investigated the co‐assembly behavior of these two modules (Figure 1a). Upon mixing JQ1‐CBT and Pep‐PpIX at a molar ratio of 2:1 in aqueous solution, driven by hydrophobic interactions and π‐π stacking, the hydrophobic JQ1 motifs and porphyrin rings clustered to form a dense core, while the hydrophilic peptide sequences extended outward. This process spontaneously yielded the bioactive self‐delivering SNAP‐TAC nanoparticles. Macroscopic observation provided intuitive evidence for this successful co‐assembly. As shown in Figure S12, the formulated SNAP‐TAC exhibited excellent aqueous dispersibility, forming a clear and homogeneous solution. In stark contrast, both the physical mixture of the truncated peptide with the ligand (PC4+JQ1‐CBT) and the pre‐synthesized small‐molecule conjugate lacking the hydrophilic sequences (JPC4) displayed severe aggregation and massive precipitation in aqueous media. Transmission electron microscopy (TEM) revealed that SNAP‐TAC exhibited a uniform, well‐dispersed spherical morphology with diameters of approximately 100 nm (Figure 1b). Dynamic light scattering (DLS) indicated a hydrodynamic diameter of 158.40 nm with a low polydispersity index (PDI) of 0.10 (Figure 1c). Furthermore, energy‐dispersive x‐ray spectroscopy (EDS) elemental mapping (Figure 1d) demonstrated that the chlorine (Cl) signal from JQ1‐CBT colocalized precisely with the carbon (C), nitrogen (N), oxygen (O), and sulfur (S) signals of the peptide module, confirming their integration at the nanoscale. To further investigate the surface elemental composition, x‐ray photoelectron spectroscopy (XPS) analysis was performed. The XPS survey spectrum (Figure 1e) confirmed the presence of C, N, O, S, and Cl elements. Specifically, the high‐resolution spectrum of Cl 2p (Figure 1f) displayed the characteristic doublet (Cl 2p_3/2_ and Cl 2p_1/2_) of the JQ1 motif, providing compositional evidence for the co‐assembly of both components.

UV–vis spectroscopy (Figure 1g) further revealed the photophysical state of the photosensitizer. Compared to the aggregation‐induced spectral broadening of free PpIX, Pep‐PpIX exhibited a distinct, sharp Soret band, indicating that the sterically demanding peptide backbone effectively isolates the porphyrin rings, thereby suppressing π‐π quenching [28, 29]. Crucially, the co‐assembled SNAP‐TAC retained this sharp absorption profile. This observation demonstrates that despite the incorporation of hydrophobic JQ1‐CBT to form a dense core, the porphyrin molecules maintain an unquenched and photoactive state. Regarding the assembly thermodynamics, the system exhibited a low critical micelle concentration (CMC) of 0.94 µM (Figure 1h). This notably low CMC value suggests a strong thermodynamic driving force for the co‐assembly, which may favor the maintenance of nanoparticle integrity upon systemic administration [30]. This spontaneous co‐assembly in aqueous media significantly streamlines the formulation process [10].

The nanoparticles exhibited a positive surface charge (Zeta potential of approximately +45 mV) (Figure S10). This characteristic may facilitate cellular internalization via electrostatic interactions across the negatively charged cell membrane provide strong electrostatic repulsion to prevent inter‐particle aggregation [31, 32]. Over a 7‐day monitoring period, the nanoparticles maintained excellent colloidal stability in physiological saline with no significant fluctuations in size or PDI (Figure 1i). The physiological suitability of the formulation was further supported by its stability in media containing 10% fetal bovine serum (FBS) (Figure S11) and a low in vitro hemolysis rate (∼5%) even at a Pep‐PpIX equivalent concentration of 1000 µM (Figure 1j). Collectively, these results validate the successful construction of a stable, bioactive self‐delivery platform suited for subsequent photo‐immunotherapy.

To elucidate the co‐assembly mechanism of the bioactive self‐delivering SNAP‐TAC nanoparticles at the atomic level, all‐atom molecular dynamics (MD) simulations were performed. As shown in the simulation snapshots (Figure 1k), the precursor molecules (Pep‐PpIX and JQ1‐CBT) were initially dispersed in an explicit aqueous environment with a minimum intermolecular distance of 10.0 Å (0 ns) to ensure an unbiased starting state. Throughout the 100 ns trajectory, these molecules spontaneously aggregated and collapsed into a compact nanocluster (25–100 ns). This assembly process is driven by intermolecular non‐covalent interactions, including hydrophobic effects, hydrogen bonding, and π‐π stacking between the porphyrin cores and JQ1‐CBT molecules. The stable conformation obtained at 100 ns is consistent with the bioactive self‐delivery observed experimentally, theoretically supporting the self‐assembly behavior.

### 2.2 Microenvironment‐Triggered In Situ Activation and ROS Generation

**FIGURE 2:**
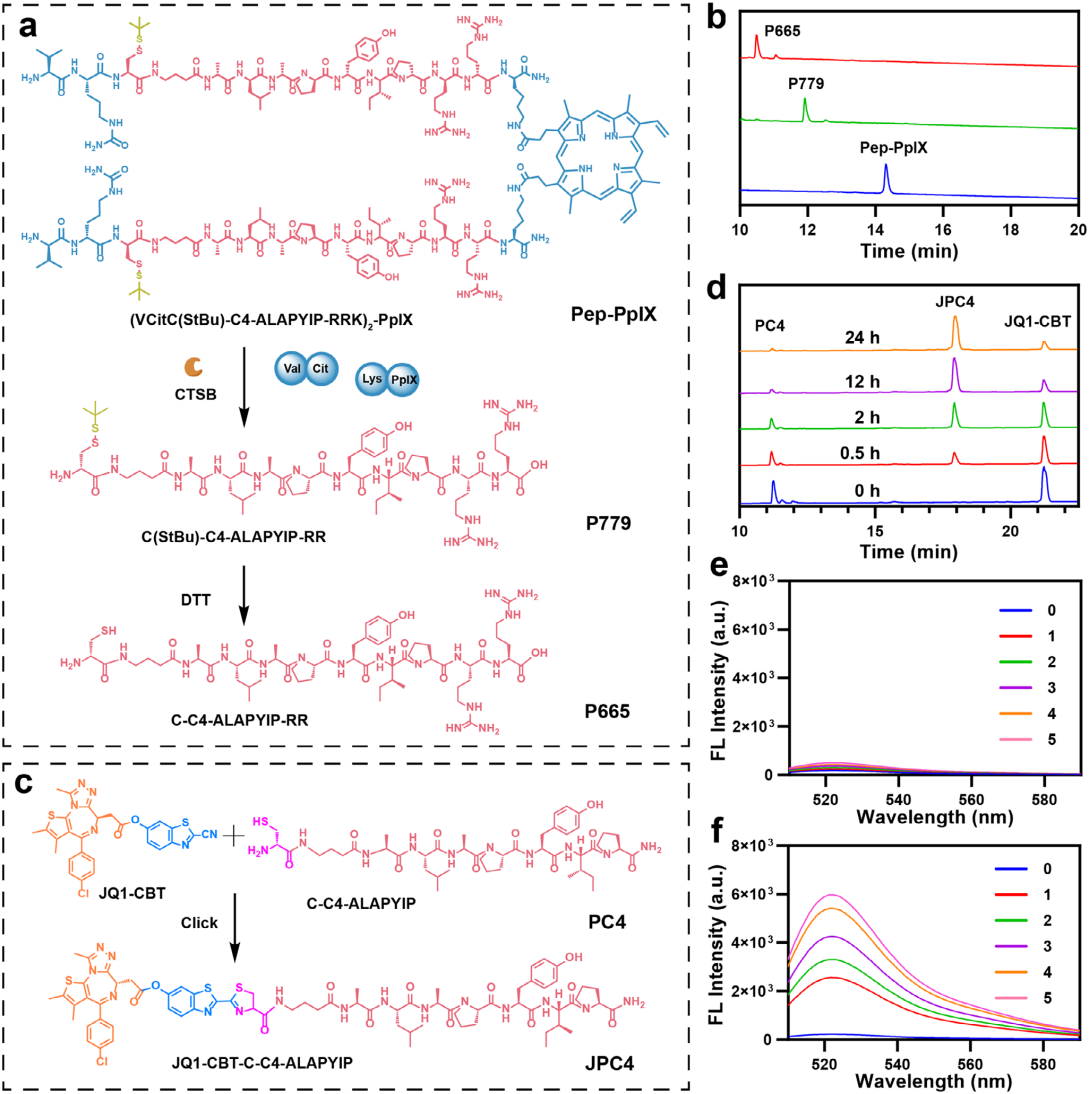
Microenvironment‐triggered in situ click assembly mechanism and evaluation of ROS generation. (a) Scheme of CTSB enzymatic cleavage and subsequent DTT reduction of Pep‐PpIX to expose the reactive 1,2‐aminothiol fragment (P665). (b) HPLC chromatograms of Pep‐PpIX before and after sequential incubation with CTSB and DTT. (c) Scheme of the metal‐free bioorthogonal click reaction between the targeting ligand precursor (JQ1‐CBT) and the model active intermediate (PC4) to form the intact degrader (JPC4). (d) Time‐dependent HPLC chromatograms monitoring the click reaction process over 24 h. (e, f) Time‐dependent fluorescence spectra of DCFH‐DA indicating reactive oxygen species (ROS) generation in (e) PBS and (f) SNAP‐TAC under 660 nm laser irradiation (+).

Having established the structural integrity and colloidal stability of the nanodelivery system, we further investigated the stimuli‐responsive activation mechanism of SNAP‐TAC in vitro. To validate the necessity of both specific stimuli, the intact precursor Pep‐PpIX was first subjected to single‐stimulus treatments. HPLC analyses revealed that treatment with DTT alone merely reduced the disulfide bond to yield intermediate P886 (Figures S17a and S18), whereas treatment with CTSB alone resulted in dual‐site enzymatic cleavage to generate intermediate P779 (Figures S17b and S13). Under model dual‐stimulus conditions, Pep‐PpIX was sequentially treated with CTSB and DTT, and HPLC analysis (Figure 2b) confirmed the complete consumption of the precursor and its clean conversion into the final active core intermediate, P665 (Figure S14). Structural analysis (Figure 2a) elucidated this dual‐responsive synergistic process: CTSB mediates specific cleavage by hydrolyzing both the N‐terminal Cit‐Cys and C‐terminal R─K amide bonds [18, 33], and DTT subsequently severs the disulfide bond to remove the *S*‐tert‐butyl (StBu) protection. This dual‐stimulus process releases the bulky photosensitizer fragment (K‐PpIX), reducing steric hindrance for both the bioorthogonal click reaction and the subsequent ternary complex formation, and exposing the reactive 1,2‐aminothiol motif on P665, providing the structural prerequisite for the downstream bioorthogonal reaction.

Subsequently, to accurately evaluate the kinetics of the metal‐free click condensation while avoiding chromatographic baseline interference from the strongly absorbing photosensitizer fragment, we designed and synthesized a simplified model intermediate (PC4, Figures 2c and S15). This model peptide omitted the photosensitizer module, retaining only the intact VHL‐recruiting ligand and the reactive cysteine motif. Following the mixing of PC4 with JQ1‐CBT, HPLC monitoring (Figure 2d) clearly elucidated this in situ bioorthogonal condensation, showing that with prolonged incubation, the precursor peaks gradually decreased, while the peak of the intact PROTAC product (JPC4, Figure S16) formed via covalent linkage steadily rose and emerged as the predominant species. These independent in vitro studies provide a molecular basis for the TME‐triggered in situ synthesis of the sterically unhindered BRD4 degrader.

Finally, having validated the dual‐responsive activation pathway, we evaluated the photodynamic potential of this platform by assessing its capacity to generate reactive oxygen species (ROS) using the DCFH‐DA probe. As shown in Figure 2f, under 660 nm laser irradiation (+), the co‐assembled SNAP‐TAC exhibited a time‐dependent ROS generation profile. In contrast to the negligible background signal observed in the PBS control group under identical irradiation (Figure 2e and Figure S20), this result confirms that the compact nano‐assembly backbone does not quench the photophysical activity of the integrated porphyrin molecules. This efficient ROS generation capacity serves not only as the primary mechanism for inducing oxidative stress in target cells but also as a crucial prerequisite for triggering ICD to subsequently remodel the immunosuppressive microenvironment [34].

### 2.3 Intracellular Targeted Degradation and Synergistic Photodynamic Cytotoxicity

Building upon the structural integrity and in vitro responsiveness of SNAP‐TAC, we investigated its intracellular behavior in 4T1 tumor cells. Confocal laser scanning microscopy (CLSM) revealed that, facilitated by its positive surface charge, SNAP‐TAC was efficiently internalized. Quantitative analysis of the intracellular photosensitizer fluorescence confirmed a time‐ and concentration‐dependent accumulation profile (Figure S21).

**FIGURE 3:**
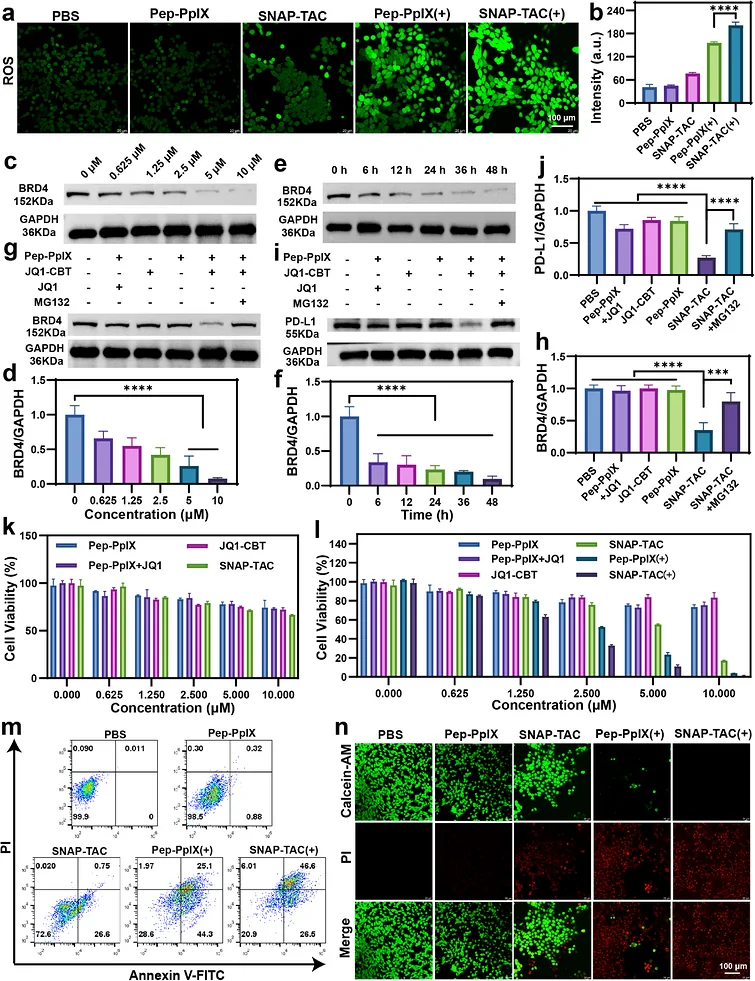
In vitro intracellular ROS generation, targeted degradation mechanisms, and synergistic therapeutic efficacy of SNAP‐TAC. (a) Representative CLSM images and (b) quantitative analysis of intracellular ROS generation detected by the DCFH‐DA probe under various treatments. Scale bars = 100 µm. Data are presented as mean ± SD (*n* = 3). One‐way ANOVA with Tukey's post hoc test, **** *P* < 0.0001. (c–f) Western blot analyses and the corresponding densitometry showing BRD4 degradation in 4T1 cells treated with SNAP‐TAC at (c, d) varying concentrations and (e, f) for different incubation periods. Data are presented as mean ± SD (*n* = 3). One‐way ANOVA with Tukey's post hoc test, **** *P* < 0.0001. Mechanistic validation of (g, h) BRD4 and (i, j) downstream PD‐L1 downregulation under different control treatments, confirming the ubiquitin‐proteasome dependency via rescue experiments with the proteasome inhibitor MG132. Data are presented as mean ± SD (*n* = 3). One‐way ANOVA with Tukey's post hoc test, *** *P* < 0.001, **** *P* < 0.0001. Relative cell viabilities of (k) normal 3T3 cells and (l) 4T1 tumor cells treated with various formulations for 48 h; (+) indicates 660 nm laser irradiation. Data are presented as mean ± SD (*n* = 3). (m) Flow cytometry analysis of apoptosis in 4T1 cells using Annexin V‐FITC/PI dual staining. (n) Live/dead cell staining of 4T1 cells (green: Calcein‐AM for live cells; red: PI for dead cells). Scale bars = 100 µm.

Following efficient cellular uptake, we evaluated the photodynamic ROS generation capacity within 4T1 cells using the DCFH‐DA probe. As depicted in Figure 3a,b, while the non‐irradiated groups exhibited negligible background signals, 660 nm laser irradiation (+) triggered intracellular ROS generation in the SNAP‐TAC(+) group, surpassing that of the monotherapy Pep‐PpIX(+) group. This amplified photodynamic efficacy is primarily attributed to the enhanced endocytotic accumulation mediated by the nano‐assembly, which prevents the premature clearance and self‐quenching often associated with free porphyrins, thereby inflicting severe subcellular oxidative stress upon photoactivation [35].

Concomitant with this photodynamic stress, the internalized SNAP‐TAC underwent intracellular dual‐responsive activation and in situ bioorthogonal condensation to synthesize the active BRD4 degrader. To verify this intracellular transformation, high‐resolution mass spectrometry (HRMS) analysis of 4T1 cell lysates was conducted. The characteristic isotopic peaks of the generated PROTAC were detected, providing chemical evidence for the in situ construction of the active chimera (Figure S19). To evaluate target‐specific degradation, systematic Western blot analyses were performed. SNAP‐TAC downregulated intracellular BRD4 protein levels in a concentration‐ and time‐dependent manner (Figure 3c–f and Figures S22 and S23). To determine whether this degradation is dependent on CTSB‐mediated enzymatic activation, the CTSB‐specific inhibitor CA‐074 ME was utilized. Pre‐treatment with CA‐074 ME effectively attenuated SNAP‐TAC‐induced BRD4 degradation (Figure S26), supporting the requisite role of CTSB cleavage in prodrug activation. Furthermore, this target depletion was rescued by pre‐treatment with the proteasome inhibitor MG132 (Figures 3g,h and S24) [36], indicating proteasome‐dependent degradation by the in situ‐synthesized chimera. The targeted degradation of the pivotal epigenetic regulator BRD4 initiated a downstream suppression of PD‐L1 protein expression. This PD‐L1 downregulation was also reversed by MG132 (Figure 3i,j and Figure S25), consistent with the direct regulatory axis and providing a molecular foundation for modulating the immunosuppressive tumor microenvironment.

To evaluate the cytotoxicity and selectivity of this dual‐mode therapeutic strategy, CCK‐8 assays were conducted. Both SNAP‐TAC and the free precursor JQ1‐CBT exhibited lower cytotoxicity toward normal NIH‐3T3 fibroblasts than the pre‐synthesized active PROTAC JPC4 over the tested concentration range (Figure 3k and Figure S28) [27]. This differential cytotoxicity was further examined by Western blot profiling (Figure S27): while JPC4 induced MG132‐reversible BRD4 degradation in normal fibroblasts, SNAP‐TAC treatment did not significantly alter BRD4 levels. This observation indicates that SNAP‐TAC activation and BRD4 degradation are limited in normal NIH‐3T3 fibroblasts, which may help minimize potential off‐target toxicity. In 4T1 cells, the pre‐synthesized active PROTAC JPC4 induced dose‐dependent cytotoxicity (Figure S29). Under dark conditions, SNAP‐TAC induced moderate cytotoxicity accompanied by BRD4 degradation. Upon 660 nm laser irradiation (+), 2.5 µM of SNAP‐TAC reduced tumor cell viability to approximately 30%, which was more effective than the respective monotherapies(Figure 3l).

This synergistic effect is further characterized by an altered cell death profile, as evaluated by flow cytometry (Figure 3m). Quantitative analysis revealed that SNAP‐TAC(+) induced a statistically significant increase in the total percentage of apoptotic cells (including both early and late apoptosis) compared to the Pep‐PpIX(+) monotherapy (Figure S30). Correspondingly, live/dead cell staining displayed enhanced red fluorescence in the irradiated groups, indicating cell membrane permeabilization and cell death (Figure 3n). Collectively, these results indicate enhanced cytotoxicity following the combination of in situ targeted protein degradation and photodynamic oxidative stress.

### 2.4 In Vitro ICD and Dendritic Cell Maturation

**FIGURE 4:**
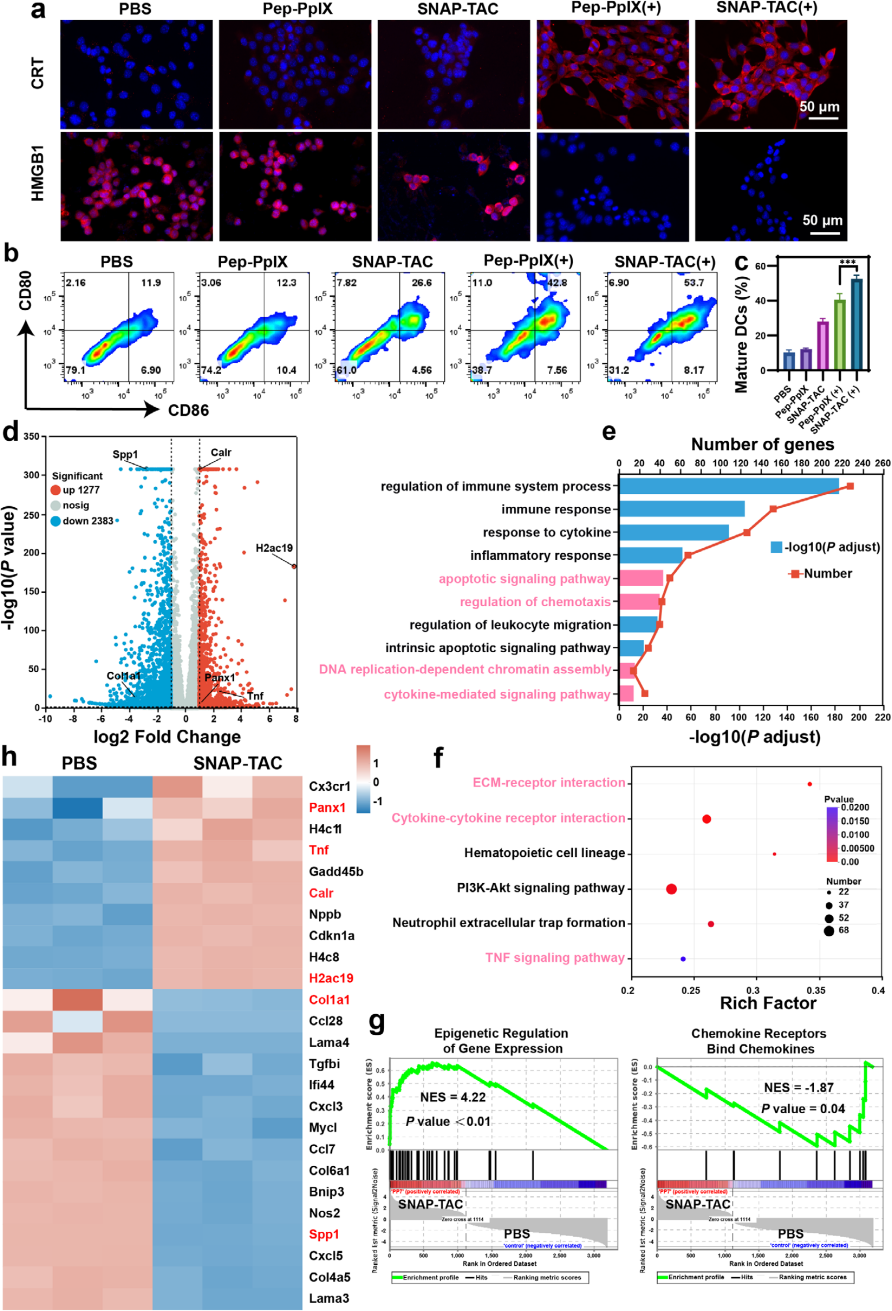
In vitro induction of ICD and corresponding transcriptomic profiling mediated by SNAP‐TAC. (a) Representative immunofluorescence images showing the cell‐surface translocation of calreticulin (CRT, red) and the nuclear depletion of HMGB1 (red) in 4T1 cells after various treatments. Nuclei were stained with DAPI (blue). Scale bars = 50 µm. (b) Representative flow cytometry scatter plots and (c) corresponding quantitative analysis showing the proportion of CD80^+^ CD86^+^ mature BMDCs following co‐incubation with the supernatants of pre‐treated 4T1 cells. Data are presented as mean ± SD (*n* = 3). One‐way ANOVA with Tukey's post hoc test, *** *P* < 0.001. (d) Volcano plot showing 3,660 DEGs between the control and SNAP‐TAC treated 4T1 cells (without irradiation). (e) GO enrichment and (f) KEGG pathway analysis of the DEGs. (g) GSEA plots showing positive enrichment in the "EPIGENETIC REGULATION OF GENE EXPRESSION" and negative enrichment in the "CHEMOKINE RECEPTORS BIND CHEMOKINES" pathway. (h) Hierarchical clustering heatmap of 25 representative DEGs, illustrating changes in epigenetic components (e.g., *H2ac19, H4c11*), ICD/immune‐related genes (e.g., *Calr, Panx1, Tnf*), and structural/oncogenic factors (e.g., *Col1a1, Lama4, Spp1, Mycl*).

Building upon the validated photodynamic ROS generation, we evaluated the capacity of the SNAP‐TAC platform to induce ICD in tumor cells. A hallmark of ICD is the release and exposure of damage‐associated molecular patterns (DAMPs), which serve as critical danger signals to prime the immune system. We first evaluated the cell‐surface translocation of calreticulin (CRT), a potent “eat‐me” signal for phagocytes. Immunofluorescence imaging (Figure 4a and Figure S31) revealed that while the PBS control exhibited negligible membrane‐associated CRT signal, SNAP‐TAC(+)‐treated 4T1 cells displayed distinct red fluorescence on the cellular membranes, indicating substantial CRT exposure.

In parallel, we assessed the subcellular localization of high mobility group box 1 (HMGB1), another DAMP. As observed in the confocal images (Figure 4a and Figure S31), the PBS control maintained a strong, localized nuclear HMGB1 fluorescence. In stark contrast, the SNAP‐TAC(+) treatment induced a prominent attenuation and near‐complete loss of this intranuclear signal. Together, the concurrent membrane exposure of CRT and the marked nuclear depletion of HMGB1 are consistent with the induction of an immunogenic cell‐death phenotype by SNAP‐TAC(+).

Extracellularly released DAMPs act as endogenous adjuvants that engage pattern recognition receptors on immature dendritic cells (DCs) to trigger maturation. To evaluate the functional consequence of this DAMP‐mediated priming process, bone marrow‐derived dendritic cells (BMDCs) were co‐incubated with the supernatants of pre‐treated 4T1 cells. Flow cytometry analysis (Figure 4b,c) revealed that the supernatant from the SNAP‐TAC(+) treatment significantly promoted DC activation, increasing the proportion of mature DCs (CD80^+^ CD86^+^) to 52.5%, which was substantially higher than that of the monotherapy or control groups. This efficient immune priming provides in vitro evidence linking photodynamic ICD to DC maturation and informs the subsequent evaluation of in vivo immune microenvironment remodeling.

### 2.5 Transcriptomic Analysis of Epigenetic Modulation and Immune Priming

To elucidate the global molecular alterations induced by SNAP‐TAC, whole‐transcriptome RNA‐seq was performed on 4T1 cells treated with SNAP‐TAC in the absence of laser irradiation. Differential expression analysis identified 3660 differentially expressed genes (DEGs) (1277 upregulated and 2383 downregulated) in the SNAP‐TAC group compared to the control (Figure 4d). Notably, while prior Western blot analysis confirmed the depletion of the BRD4 protein (Section 2.3), *Brd4* mRNA levels remained unchanged in the transcriptomic dataset. This observation is consistent with a post‐translational degradation mechanism via the ubiquitin‐proteasome system, confirming the PROTAC‐mediated targeted degradation mechanism [37].

Subsequent Gene Ontology (GO) and KEGG pathway analyses indicated that these DEGs were primarily enriched in pathways associated with the regulation of immune system processes, cytokine‐cytokine receptor interaction, and extracellular matrix (ECM)‐receptor interaction (Figure 4e,f). Gene Set Enrichment Analysis (GSEA) further revealed that a prominently enriched gene set was “epigenetic regulation of gene expression” (normalized enrichment score (NES) = 4.22) (Figure 4g), aligning with the biological function of BRD4 as an epigenetic regulator [38]. Conversely, pathways associated with immune‐and microenvironment‐related processes, including chemokine receptor interactions (Figure 4g) and TGF‐β signaling (Figure S32), exhibited significant negative enrichment [39], providing a molecular basis for the observed modulation of the immunosuppressive tumor microenvironment.

Specific gene expression signatures were further delineated using a hierarchical clustering heatmap (Figure 4h). SNAP‐TAC treatment was associated with the upregulation of core histone genes (e.g., *H2ac19*, *H4c8*, *H4c11*), consistent with the characteristic remodeling of the epigenetic landscape previously observed upon BRD4 depletion. Concurrently, a distinct transcriptional upregulation was observed for the ICD marker calreticulin (*Calr*), the ATP‐release channel pannexin‐1 (*Panx1*), and the pro‐inflammatory cytokine TNF‐α (*Tnf*) [40]. While the physical execution of ICD requires acute photodynamic stress, this distinct transcriptomic profile suggests that dark‐state SNAP‐TAC treatment intrinsically pre‐sensitizes tumor cells toward an immunogenic phenotype at the transcriptional level, even prior to ROS generation. Furthermore, key structural ECM components (e.g., *Col1a1*, *Lama4*, *Col4a5*) [41] and oncogenic factors (e.g., *Spp1*, *Mycl*) [42] were substantially downregulated. Collectively, these transcriptomic results highlight the functional contribution of introducing the PROTAC module: BRD4 degradation is associated with transcriptional changes consistent with epigenetic modulation, reduced immunosuppressive signaling, and increased expression of intrinsic danger signals (e.g., *Calr*, *Panx1*, *Tnf*). This transcriptional modulation establishes a tumor‐cell state that is potentially more responsive to subsequent photodynamic and immune responses, providing a coherent mechanistic rationale for our dual‐modality design.

### 2.6 In Vivo Biodistribution and Theranostic Potential of SNAP‐TAC

**FIGURE 5:**
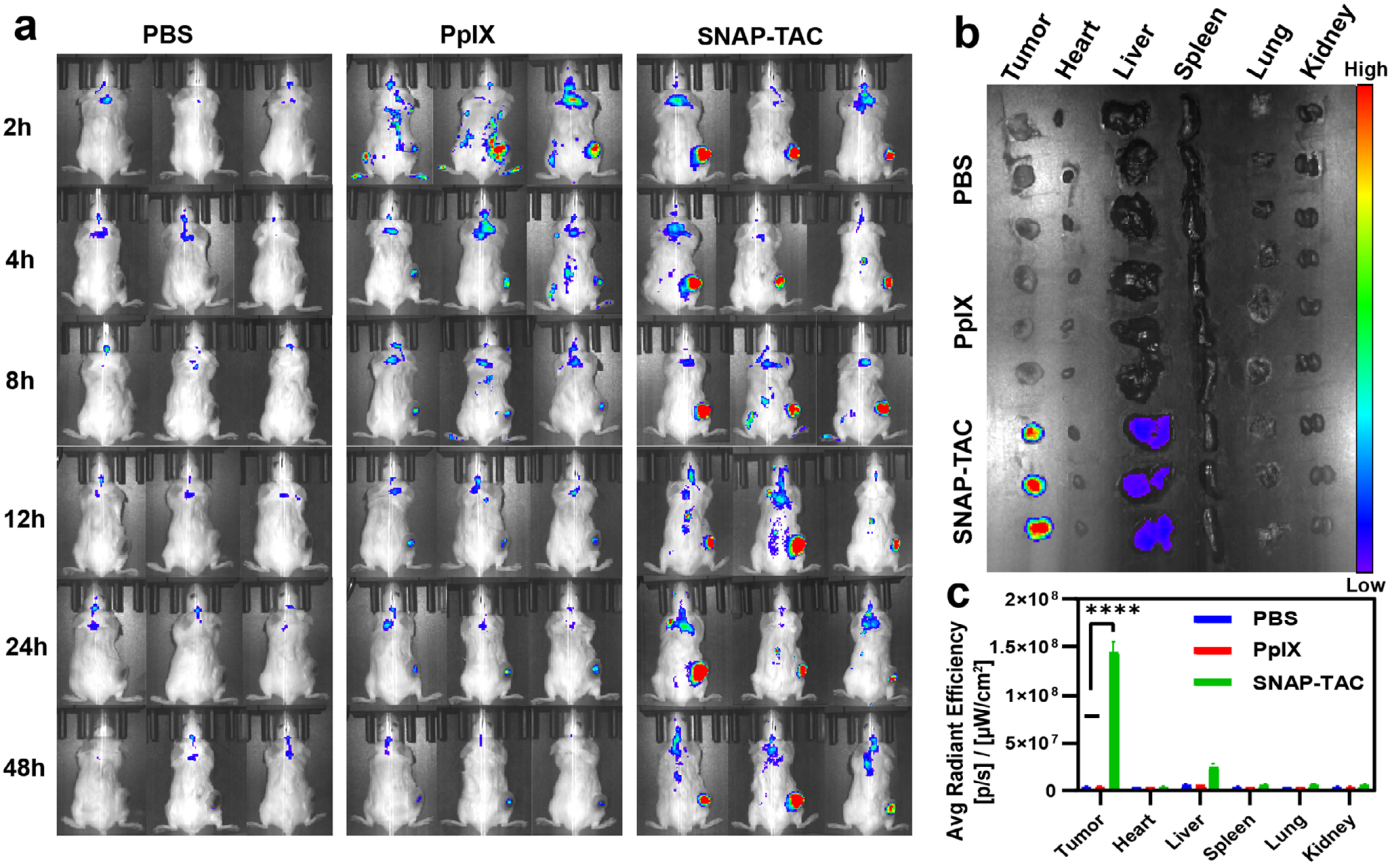
In vivo biodistribution and tumor‐targeting capability of SNAP‐TAC. (a) Time‐dependent in vivo fluorescence imaging of 4T1 tumor‐bearing mice at 2, 4, 8, 12, 24, and 48 h post‐intravenous injection of PBS, free PpIX, and SNAP‐TAC. (b) Ex vivo fluorescence imaging of the excised tumors and major organs (heart, liver, spleen, kidney, lung) collected at 48 h post‐injection. (c) Corresponding quantitative analysis of the fluorescence intensity in the excised organs and tumors. Data are presented as mean ± SD (*n* = 3). Two‐way ANOVA with Tukey's post hoc test, **** *P* < 0.0001.

To evaluate the spatiotemporal distribution and tumor‐targeting profile of the nano‐assemblies, serial in vivo fluorescence imaging was performed on 4T1 tumor‐bearing mice over a 48‐h period (Figure 5a). The intrinsic fluorescence of the PpIX moiety endows SNAP‐TAC with self‐imaging capabilities for theranostic applications. Following intravenous administration, the PBS control group maintained a low baseline autofluorescence [43]. In the initial phase (∼2 h post‐injection), both the free PpIX control and the SNAP‐TAC groups displayed observable fluorescence at the tumor site. However, their subsequent retention kinetics exhibited distinct divergence. The fluorescence signal of free PpIX diminished rapidly over time. In contrast, SNAP‐TAC, with its nanoscale dimensions and colloidal stability, exhibited sustained tumor‐associated fluorescence consistent with enhanced tumor accumulation and retention through the enhanced permeability and retention (EPR) effect [44, 45]. The target‐site fluorescence in the SNAP‐TAC group remained prominent for up to 48 h, effectively overcoming the rapid clearance limitation of the free photosensitizer.

At the 48‐h endpoint, major organs and tumors were excised for ex vivo imaging to validate the in vivo observations (Figure 5b). The ex vivo tumors from the SNAP‐TAC group displayed intense fluorescence, which was substantially higher than that of the PpIX control, as further corroborated by quantitative analysis (Figure 5c). Additionally, the prominent fluorescence observed in the liver is consistent with RES‐mediated hepatic uptake and hepatobiliary processing, which is commonly observed for positively charged nanoparticles [46, 47, 48]. Overall, these biodistribution profiles demonstrated pronounced tumor‐associated fluorescence and prolonged signal retention of SNAP‐TAC. Furthermore, this real‐time fluorescence tracking highlights the theranostic capability of the system, guiding the selection of 24 h post‐injection as the time point for subsequent localized irradiation.

### 2.7 In Vivo Synergistic Anti‐Tumor Efficacy of SNAP‐TAC

**FIGURE 6:**
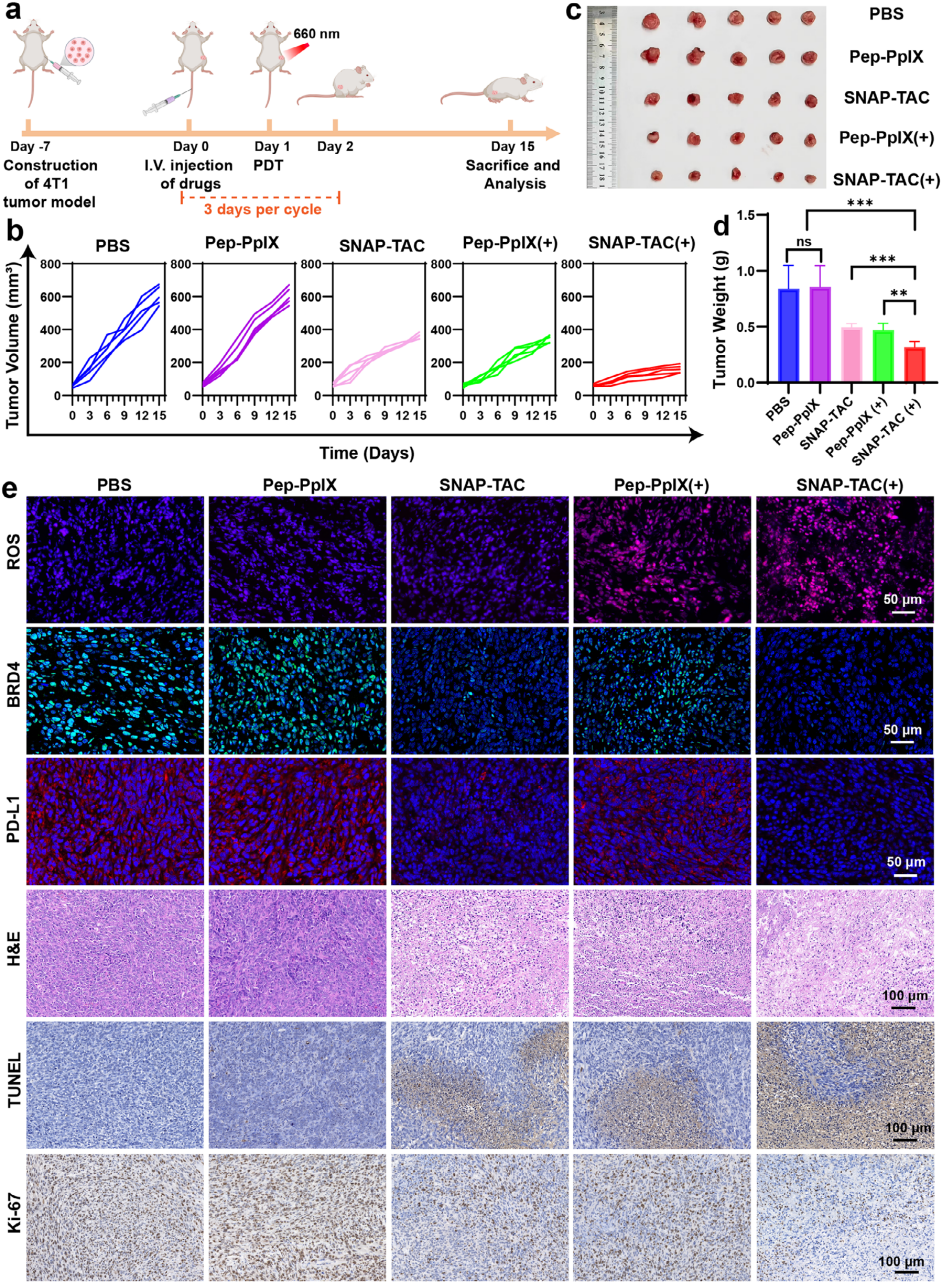
In vivo synergistic anti‐tumor efficacy of the SNAP‐TAC platform. (a) Schematic timeline of the in vivo 4T1 tumor model establishment and the corresponding treatment regimen. (b) Individual tumor growth kinetics of mice over 15 days following various treatments (*n* = 5). (c) Photograph of the excised tumors collected at the therapeutic endpoint. (d) Corresponding average tumor weights of the excised tumors. Data are presented as mean ± SD (*n* = 5). One‐way ANOVA with Tukey's post hoc test, ** *P* < 0.01, *** *P* < 0.001, ns: not significant. (e) Representative immunofluorescence (ROS, BRD4, PD‐L1) and histological staining (H&E, TUNEL, Ki‐67) images of tumor slices from different treatment groups. Scale bars: 50 µm (ROS, BRD4, PD‐L1) and 100 µm (H&E, TUNEL, Ki‐67).

To evaluate the in vivo therapeutic efficacy of the SNAP‐TAC platform, a 4T1 tumor‐bearing mouse model was established (Figure 6a). As depicted in the tumor growth kinetics (Figure 6b), the PBS and non‐irradiated Pep‐PpIX groups exhibited rapid tumor progression, indicating negligible therapeutic activity without light activation. Monotherapies relying solely on targeted degradation (SNAP‐TAC without irradiation) or photodynamic therapy (Pep‐PpIX(+)) provided only moderate tumor inhibition, highlighting the inherent limitations of single‐modality treatments against aggressive triple‐negative breast cancer (TNBC). In contrast, the SNAP‐TAC(+) treatment produced the most pronounced inhibition of tumor growth. This macroscopic efficacy was further corroborated by the photographic records and average weight of the excised tumors at the endpoint (Figure 6c,d), demonstrating in vivo combined anti‐tumor effects.

Immunofluorescence and histological analyses of the tumor sections revealed molecular and histological changes associated with the enhanced therapeutic effect (Figure 6e). Under 660 nm laser irradiation, SNAP‐TAC(+) generated abundant ROS in situ, mediating primary photodynamic damage. Crucially, the in situ synthesized chimera achieved efficient depletion of BRD4, which was accompanied by a concomitant reduction of the downstream immune checkpoint PD‐L1. This targeted intervention effectively circumvents a critical bottleneck of conventional photodynamic treatments, as recent studies have unmasked that ROS‐driven stress inherently reprograms tumor immune signaling to upregulate PD‐L1, thereby inducing adaptive immune resistance [49]. Driven by this integration of phototoxicity and targeted degradation, the SNAP‐TAC(+) group exhibited the most extensive tissue damage, characterized by extensive necrosis (H&E), widespread apoptosis (TUNEL), and suppressed proliferative activity (diminished Ki‐67 expression).

Notably, while our in vitro results indicated a more pronounced direct cytotoxic effect of PDT compared to BRD4 degradation alone, this efficacy gap was substantially narrowed in vivo. This discrepancy highlights the distinct mechanisms of action within the complex tumor microenvironment (TME). In vitro, PDT rapidly eliminates tumor cells via acute oxidative stress, whereas PROTACs generally exhibit slower cytostatic effects in monocultures. In vivo, however, the direct efficacy of PDT may be attenuated by solid tumor hypoxia and limited light penetration depth [50]. Conversely, the therapeutic impact of BRD4 degradation may have additional consequences in vivo through modulation of the immunosuppressive TME and engagement of host immune responses [51, 52]. This biological contrast further rationalizes the necessity of our SNAP‐TAC platform, which unifies PDT‐triggered ICD with PROTAC‐mediated epigenetic immune modulation to overcome their respective monotherapy limitations.

Furthermore, SNAP‐TAC demonstrated a favorable systemic biosafety profile. Throughout the treatment period, the body weights of the mice in all groups increased steadily without abnormal fluctuations (Figure S33). Comprehensive hematological analysis, serum biochemical profiling of hepatic (alanine aminotransferase, ALT; aspartate aminotransferase, AST; alkaline phosphatase, ALP) and renal (urea, creatinine) functions, and H&E evaluations of major organs revealed no noticeable overt pathological abnormalities (Figures S34 and S35). In addition, BRD4 immunofluorescence and quantitative analysis in the spleen, a representative mononuclear phagocyte system organ, showed no significant difference between the PBS and SNAP‐TAC groups (Figure S36), indicating no apparent reduction in splenic BRD4 expression under the tested conditions. Collectively, these findings indicate that the treatment was well tolerated under the tested conditions.

### 2.8 In Vivo Remodeling of the Tumor Immune Microenvironment

**FIGURE 7:**
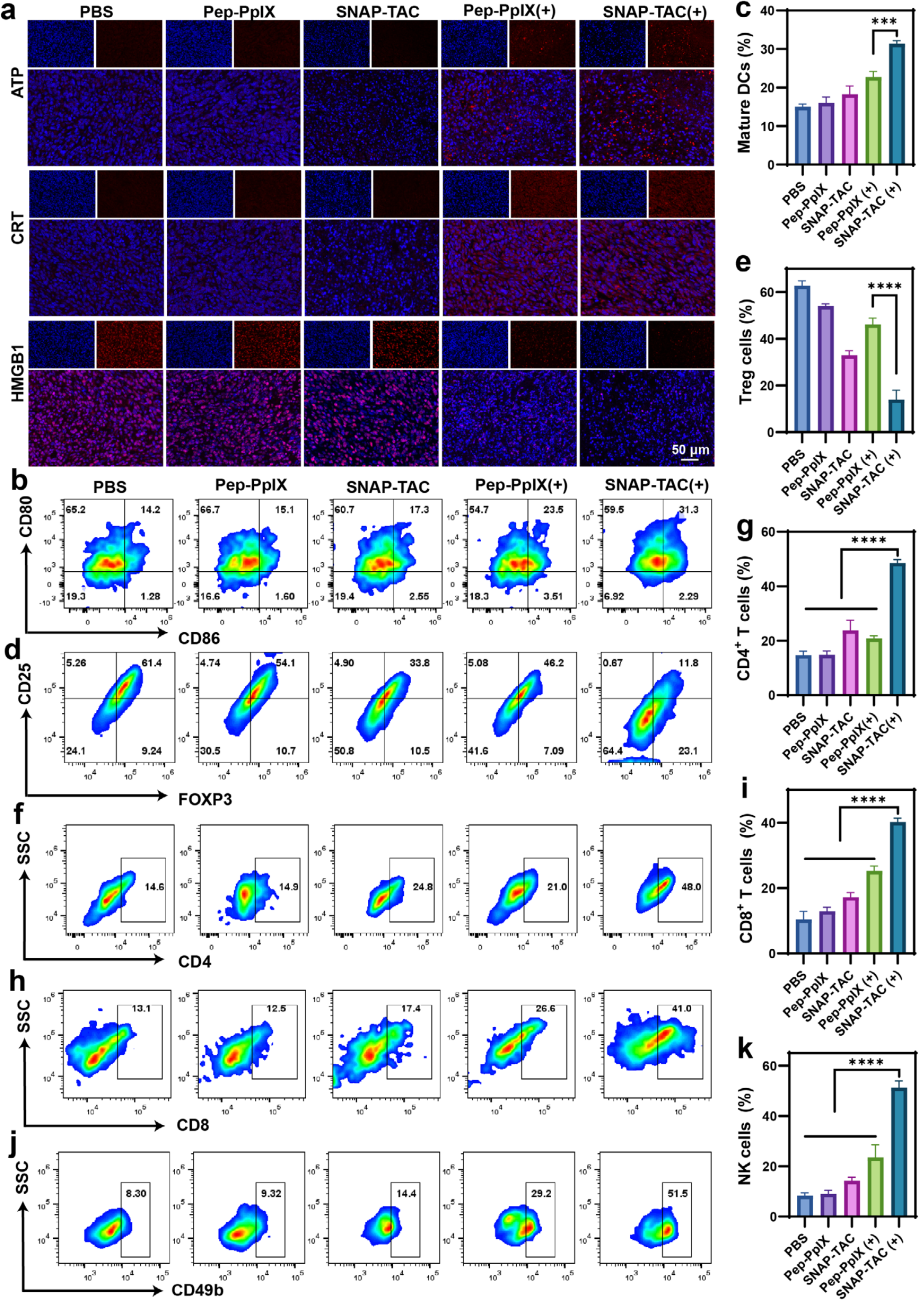
In vivo remodeling of the TME mediated by SNAP‐TAC. (a) Representative immunofluorescence images showing increased ATP‐associated signal, CRT exposure, and nuclear HMGB1 depletion in tumor sections from different treatment groups. Scale bars = 50 µm. Flow cytometry pseudocolor plots and the corresponding quantitative analysis of (b, c) mature dendritic cells (DCs, CD80^+^ CD86^+^) in tumor‐draining lymph nodes, as well as tumor‐infiltrating (d, e) regulatory T cells (Tregs, CD25^+^ FOXP3^+^), (f, g) helper T cells (CD3^+^ CD4^+^), (h, i) cytotoxic T cells (CD3^+^ CD8^+^), and (j, k) natural killer (NK) cells (CD3^−^ CD49b^+^). Data are presented as mean ± SD (*n* = 3). One‐way ANOVA with Tukey's post hoc test, *** *P* < 0.001, **** *P* < 0.0001.

To evaluate the capacity of the SNAP‐TAC platform to reverse the highly immunosuppressive microenvironment of TNBC, the in vivo induction of ICD was initially assessed via immunofluorescence (Figure 7a). The SNAP‐TAC(+) group exhibited the most pronounced ICD‐associated changes of damage‐associated molecular patterns (DAMPs), characterized by increased ATP‐associated signal, CRT surface exposure, and nuclear HMGB1 depletion consistent with its release. This DAMP‐mediated signaling indicated an immunogenic phenotype in the dying tumor cells.

Flow cytometry analysis of tumor‐draining lymph nodes confirmed that the SNAP‐TAC(+) intervention promoted antigen‐presenting cell activation, elevating the proportion of mature dendritic cells (DCs, CD80^+^ CD86^+^) to 31.2%, which was higher than that in monotherapy and PBS control groups (Figure 7b,c and Figure S37). Beyond initiating immune priming, TNBC typically features a dense immunosuppressive barrier that impedes effector cell infiltration. In addition to PD‐L1 downregulation, SNAP‐TAC(+) reduced the population of tumor‐infiltrating regulatory T cells (Tregs, CD25^+^FOXP3^+^), decreasing to 11.9% in the SNAP‐TAC(+) group (Figure 7d,e and Figure S38).

Concomitant with the reversal of the immunosuppressive barrier, an enhanced infiltration of cytolytic lymphocytes was achieved. In SNAP‐TAC(+)‐treated tumors, the populations of helper CD4^+^ T cells and cytotoxic CD8^+^ T cells increased to 52.1% and 40.9%, respectively (Figure 7f–i and Figures S39 and S40), representing a substantial increase compared to the PBS control. Furthermore, the recruitment of natural killer (NK) cells was similarly elevated to 53.6% (Figure 7j,k and Figure S41), establishing a comprehensive cytolytic network. Collectively, these immunological profiles demonstrate that SNAP‐TAC combines photodynamic ICD induction with PD‐L1 downregulation and Treg reduction, effectively remodeling the immunologically “cold” tumor microenvironment into a “hot” cytolytic phenotype to promote anti‐tumor immune activation.

## 3 Conclusion

In summary, we have developed a TME‐responsive, bioactive self‐delivering SNAP‐TAC theranostic platform that effectively addresses the in vivo delivery and off‐target limitations of conventional PROTACs. Triggered by elevated intratumoral CTSB and GSH, the system undergoes specific enzymatic cleavage and disulfide reduction to liberate the bulky photosensitizer, thereby alleviating steric hindrance and exposing the reactive 1,2‐aminothiol motif. This process drives a metal‐free CBT‐Cys bioorthogonal condensation, achieving the tumor‐selective in situ synthesis of an active BRD4 degrader. Crucially, the intrinsic fluorescence of the photosensitizer allows for real‐time tracking to guide precise localized irradiation. Mechanistically, the in situ‐generated BRD4‐degrading PROTAC exerts profound immunomodulatory effects via PD‐L1 downregulation and transcriptional modulation, which synergistically complement PDT‐induced ICD. This integration successfully remodels the immunosuppressive tumor microenvironment, promoting robust anti‐tumor immune activation. Furthermore, the modular nature of SNAP‐TAC highlights its potential versatility; substitution of the targeting ligand may enable its future application to other pathological proteins. Ultimately, this study provides a powerful chemical biology strategy for integrating tumor‐associated PROTAC activation with photodynamic immunotherapy, offering a robust paradigm for next‐generation precision nanomedicines.

## 4 Experimental Section

### 4.1 Ethics Statement

All animal experiments were performed in strict compliance with the ethical guidelines and protocols approved by the Ethics Committee of Shanghai University (Approval No. ECSHU 2025‐122).

### 4.2 Statistical Analysis

GraphPad Prism software was used for statistical analysis. Results are reported as the mean ± SD from independent experiments (*n* ≥ 3). The statistical disparities between two experimental groups were analyzed using an unpaired Student's t‐test. Variations among multiple groups were assessed via one‐way or two‐way analysis of variance (ANOVA), followed by a Tukey's multiple comparisons test, depending on the number of independent variables. Differences were considered statistically significant when *P* < 0.05, denoted as * *P* < 0.05, ** *P* < 0.01, *** *P* < 0.001, and **** *P* < 0.0001 (“ns” stands for not significant).

## Author Contributions

**Shiqin Jian**: Conceptualization, Methodology, Investigation, Validation, Visualization, Data curation, Writing – original draft, Writing – review and editing. **Jiasha Wu**: Methodology, Software, Supervision. **Fusheng Xu**: Formal analysis. **Yan Zuo**: Software. **Huaxing Shen**: Formal analysis. **Rui Ji**: Software. **Luyi Wang**: Software. **Na Li**: Project administration. **Yanting Sun**: Project administration. **Yongsheng Yu**: Project administration. **Yejiao Shi**: Project administration. **Honggang Hu**: Funding acquisition, Resources. **Feng Xu**: Funding acquisition, Resources. **Dan Huang**: Funding acquisition, Resources. **Xiaochun Hu**: Conceptualization, Investigation, Supervision, Writing – original draft, Writing – review and editing.

## Funding

This work was funded by the National Natural Science Foundation of China (Grant Nos. 22477075, 82572674, and U25A20647).

## Declaration of Generative AI and AI‐Assisted Technologies in the Writing Process

During the preparation of this work, the authors used Google Gemini for language editing, including grammar, style, and clarity improvement. The authors reviewed and edited the manuscript as needed and take full responsibility for its final content.

## Conflicts of Interest

The authors declare no conflicts of interest.

## Supporting information




**Supporting File**: advs77938‐sup‐0001‐SuppMat.pdf.

## Data Availability

The raw whole‐transcriptome RNA sequencing data generated in this study have been deposited in the NCBI BioProject database and are accessible under the accession number PRJNA1512639. All other relevant data supporting the findings of this study are available within the article and its Supporting Information, or from the corresponding authors upon reasonable request.
