# Supplementary material for: In Situ Bioorthogonal Synthesis of PROTACs via Dual‐Responsive Cleavage for Synergistic Photo‐Immunotherapy
Source: Adv Sci (Weinh). 2026 Sep 27:e77938. Online ahead of print. doi: 10.1002/advs.77938 (PMC13616413; doi:10.1002/advs.77938)
Supplement: Supplementary file 1 [file ADVS-9999-e77938-s001.pdf]

Supporting Information

In Situ Bioorthogonal Synthesis of PROTACs via Dual-Responsive Cleavage for Synergistic Photo-Immunotherapy

Shiqin Jian#, Jiasha Wu#, Fusheng Xu, Yan Zuo, Huaxing Shen, Rui Ji, Luyi Wang, Na Li, Yanting Sun, Yongsheng Yu, Yejiao Shi, Honggang Hu, Feng Xu, Dan Huang*, Xiaochun Hu**

S. Jian, J. Wu, F. Xu, Y. Zuo, H. Shen, R. Ji, L. Wang, N. Li, Y. Yu, X. Hu
School of Medicine, Department of Chemistry, College of Sciences, Shanghai University, Shanghai 200444, China
E-mail: huxiaochun@shu.edu.cn; xufusheng@shu.edu.cn

Y. Sun
Department of Medical Oncology, Cancer Center, Shanghai General Hospital, Shanghai Jiao Tong University School of Medicine, Shanghai 201620, China

Y. Shi
Institute of Translational Medicine, Shanghai University, Shanghai 200444, China

H. Hu
Institute of Translational Medicine, Shanghai Jiao Tong University, Shanghai 200240, China

H. Hu
School of Pharmacy, Chengdu Medical College, Chengdu 610500, China

F. Xu
Department of Neurosurgery, Huashan Hospital, Shanghai Medical College, Fudan University, Shanghai 200040, China

D. Huang
Department of Pathology, Fudan University Shanghai Cancer Center, Shanghai 200032,

China
E-mail: danhuang@shca.org.cn

X. Hu
Shanghai Integration and Innovation Center of Marine Medical Engineering, Shanghai University, Shanghai 200444, China

S. Jian and J. Wu contributed equally to this work.

Experimental Section

Materials: Protoporphyrin IX (PpIX), (S)-tert-butyl 2-(4-(4-chlorophenyl)-2,3,9-trimethyl-6H-thieno[3,2-f][1,2,4]triazolo[4,3-a][1,4]diazepin-6-yl)acetate ((+)-JQ1), tris(2-carboxyethyl)phosphine (TCEP), 2-(1H-benzotriazol-1-yl)-1,1,3,3-tetramethyluronium hexafluorophosphate (HBTU), 1-hydroxybenzotriazole (HOBt), 2-cyanobenzothiazole (CBT), N,N-dimethylaminopyridine (DMAP), 1-ethyl-3-(3-dimethylaminopropyl)carbodiimide hydrochloride (EDC·HCl), and ethyl 2-cyano-2-(hydroxyimino)acetate (Oxyma) were purchased from Bide Pharmatech Ltd. (Shanghai, China). Trifluoroacetic acid (TFA), N,N-diisopropylethylamine (DIEA), and N,N'-diisopropylcarbodiimide (DIC) were obtained from J&K Scientific Ltd. (Beijing, China). 1,2-ethanedithiol (EDT), triisopropylsilane (TIS), and organic solvents including dichloromethane (DCM), dimethyl sulfoxide (DMSO), and acetonitrile were acquired from Sinopharm Chemical Reagent Co., Ltd. (Shanghai, China). Rink Amide-MBHA resin and Fmoc-protected amino acids were purchased from GL Biochem (Shanghai, China). 2',7'-dichlorodihydrofluorescein diacetate (DCFH-DA, Cat# S0033S), Calcein-AM/PI Double Stain Kit (Cat# C2015M), Annexin V-FITC Apoptosis Detection Kit (Cat# C1062S), CCK-8 Cell Proliferation Assay Kit (Cat# C0037), RIPA lysis buffer (Cat# P0013B), and BCA protein assay kit (Cat# P0012) were sourced from Beyotime Biotechnology (Shanghai, China). High-glucose DMEM medium (Cat# 11965092), fetal bovine serum (FBS, Cat# 10099141), and penicillin-streptomycin (Cat# 15140122) were procured from Gibco (Carlsbad, CA, USA). Collagenase Type IV (Cat# S10053), DNase I (Cat# 10607ES15), and red blood cell lysis buffer (Cat# R20172) were purchased from Yeasen Biotechnology (Shanghai, China). Primary antibodies against BRD4 (Abcam, UK, Cat# ab128874) and PD-L1 (Cat# 17952-1-AP), HMGB1 (Cat# 10829-1-AP), Calreticulin (Cat# 10292-1-AP), GAPDH (Cat# 60004-1-Ig) (Proteintech, China) were utilized for western blot and immunofluorescence. Fluorophore-conjugated anti-mouse antibodies for flow cytometry, including APC-anti-CD45 (Cat# 103111), PE-anti-CD3 (Cat# 100307), FITC-anti-CD4 (Cat# 100477), PE-Cy7-anti-CD8 (Cat# 100722), PE-anti-CD25 (Cat# 102015), BV421-anti-FOXP3 (Cat# 126404), FITC-anti-CD11c (Cat# 117363), PE-anti-CD80 (Cat# 104708), BV421-anti-CD86 (Cat# 105031), and PerCP-Cy5.5-anti-CD49b (Cat# 103519), along with TruStain FcX receptor blocking solution (Cat# 101320), were purchased from BioLegend (San Diego, CA, USA). Murine breast cancer 4T1 cells were obtained from the American Type Culture Collection (ATCC, Cat# CRL-2539, RRID: CVCL_0125).

Instruments: High-resolution mass spectrometry (HRMS) was performed on an LTQ Orbitrap Elite (Thermo Scientific, USA). Preparative and analytical HPLC were carried out on a

Prominence LC-20A system (Shimadzu, Japan). UV-Vis absorption spectra and fluorescence emission spectra were recorded on a MAPADA UV-1800 spectrophotometer (China) and a fluorescence spectrophotometer (Hitachi, Japan), respectively. Hydrodynamic size and zeta potential were measured via dynamic light scattering (DLS) on a Zetasizer Nano ZS (Malvern, UK). The morphology of nanoparticles was observed using a JEM-F200 transmission electron microscope (JEOL, Japan). Confocal fluorescence imaging was performed on a STELLARIS 8 fluorescence lifetime and super-resolution microscope (Leica Microsystems, Germany). Quantitative flow cytometric data were acquired on a flow cytometer (Beckman Coulter, USA) and processed with FlowJo software (Tree Star, USA). In vivo and ex vivo fluorescence imaging was conducted using an IVIS Spectrum system (PerkinElmer, USA).

High-Resolution Mass Spectrometry (HRMS) Analysis: High-resolution mass spectra were acquired using a Thermo Scientific mass spectrometer. The instrument was equipped with an electrospray ionization (ESI) source operating in positive-ion mode. Data acquisition was performed continuously over a full-scan mass range of m/z 200 to 2000. Prior to analysis, samples were dissolved in appropriate MS-grade solvents. The acquired exact mass data were subsequently utilized to confirm the molecular weight and composition of the target compounds.

Synthesis of Pep-PpIX: The peptide-porphyrin conjugate Pep-PpIX, with the sequence [(VCitC(StBu)-C4-ALAPYIP-RRK)$_2$-PpIX], was synthesized on Rink Amide-MBHA resin using an orthogonal Fmoc SPPS strategy. Chain elongation was performed with DIC/Oxyma as coupling agents and 20% piperidine/DMF for Fmoc deprotection. To ensure site-specific conjugation of Protoporphyrin IX (PpIX), Lys(Dde) was employed as the starting residue and the N-terminal Val was Boc-protected. After assembly, the Dde group was selectively removed with 2% hydrazine hydrate, followed by the coupling of PpIX onto the exposed Lys side chains using HBTU/HOBt/DIEA.The conjugate was cleaved from the resin using a TFA/TIS/H_2O/EDT cocktail (95:2:2:1, v/v), which concurrently removed the Boc group and other acid-labile protections while cleanly preserving the Cys(StBu) modification. The crude product was precipitated with cold isopropanol, purified by preparative HPLC, and characterized by HRMS. HRMS (ESI) *m/z*: Calcd. For $C_{188}H_{298}N_{52}O_{34}S_4$ (most abundant isotope) $[M+4H]^{4+}$ 990.5607, $[M+5H]^{5+}$ 792.6500, $[M+6H]^{6+}$ 660.7096, $[M+7H]^{7+}$ 566.4664, found: $[M+4H]^{4+}$ 990.5578, $[M+5H]^{5+}$ 792.6485, $[M+6H]^{6+}$ 660.7083, $[M+7H]^{7+}$ 566.4650.

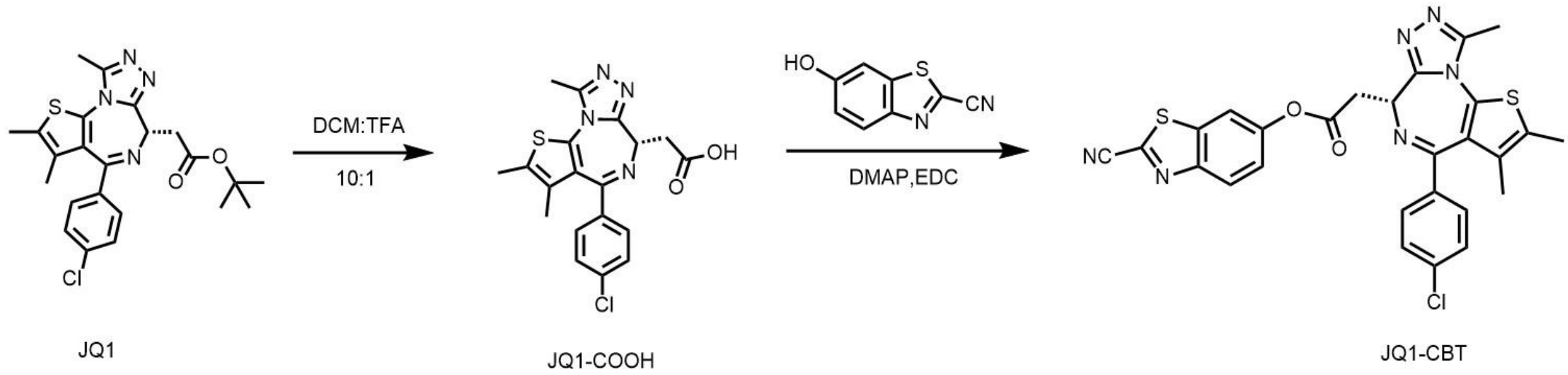

Synthesis of JQ1-COOH: (+)-JQ1 (50 mg, 0.109 mmol) was dissolved in a mixture of anhydrous DCM (2 mL) and TFA (0.2 mL). The reaction mixture was stirred at room temperature overnight. Upon completion, the solvent and excess TFA were removed under reduced pressure. The residue was co-evaporated with DCM three times to afford JQ1-COOH as a pale yellow solid, which was used in the next step without further purification. ^{1}H NMR (600 MHz, CD_3OD) δ 7.46 (d, J = 8.5 Hz, 2H), 7.41 (d, J = 8.8 Hz, 2H), 4.58 (t, J = 7.1 Hz, 1H), 3.50 (d, J = 7.1 Hz, 2H), 2.69 (s, 3H), 2.45 (s, 3H), 1.70 (s, 3H). ^{13}C NMR (151 MHz, CD_3OD) δ 173.09, 164.84, 155.48, 150.76, 136.68, 136.61, 132.15, 131.87, 130.62, 130.58, 129.93, 128.40, 53.54, 35.76, 12.96, 11.52, 10.18. HRMS (ESI) *m/z*: $[M+H]^+$ calcd for $C_{19}H_{17}ClN_4O_2S$, 401.0834; found: 401.0821.

Synthesis of JQ1-CBT: JQ1-COOH (18 mg, 0.045 mmol), CBT (15.8 mg, 0.090 mmol), and DMAP (2.7 mg, 0.0225 mmol) were dissolved in anhydrous DCM (2 mL). After stirring at room temperature for 30 minutes, the mixture was cooled to 0 °C in an ice bath. EDC·HCl (10.4 mg, 0.054 mmol) was then added to the solution. The reaction mixture was stirred at 0 °C for 15 minutes, allowed to warm to room temperature, and stirred for an additional 4 hours. The resulting mixture was concentrated and purified by preparative RP-HPLC. The target fractions were collected and lyophilized to yield JQ1-CBT as a pale yellow solid. ^{1}H NMR (600 MHz, CD_3OD) δ 8.24 (d, J = 9.0 Hz, 1H), 8.04 (d, J = 2.3 Hz, 1H), 7.53 (dd, J = 8.9, 2.3 Hz, 1H), 7.50 (d, J = 8.3 Hz, 2H), 7.44 (d, J = 8.3 Hz, 2H), 4.77 (t, J = 7.2 Hz, 1H), 3.88 (dd, J = 7.2, 1.4 Hz, 2H), 2.75 (s, 3H), 2.45 (s, 3H), 1.70 (s, 3H). ^{13}C NMR (151 MHz, CD_3OD) δ 169.46, 165.30, 150.83, 150.05, 137.43, 136.87, 136.45, 136.33, 132.40, 131.94, 130.72, 130.00, 128.51, 125.18, 122.82, 115.03, 112.42, 53.29, 36.02, 13.00, 11.55, 10.17. HRMS (ESI) *m/z*: $[M+H]^+$ calcd for $C_{27}H_{19}ClN_6O_2S_2$, 559.0772; found: 559.0763.

Preparation and characterization of SNAP-TAC: The SNAP-TAC nanomedicine was prepared via a nanoprecipitation method. Pep-PpIX (4 mg, ~1 μmol) and JQ1-CBT (1.2 mg, ~2 μmol) were co-dissolved in 20 μL of DMSO. The mixture was then rapidly dispersed into 1 mL of deionized water under continuous sonication for 10 min. The resulting dispersion was left to stand for 2 h to facilitate stable self-assembly. The final SNAP-TAC nanoparticle suspension (1

mM based on Pep-PpIX) was used directly for subsequent experiments without further purification.The hydrodynamic size, ζ-potential, and 7-day colloidal stability of SNAP-TAC were evaluated by dynamic light scattering (DLS), while its morphology was observed via transmission electron microscopy (TEM). The optical properties and characteristic absorption spectra of free PpIX, Pep-PpIX, JQ1-CBT, and the SNAP-TAC formulation were recorded using UV-Vis spectroscopy.

Determination of Critical Micelle Concentration (CMC): The CMC of SNAP-TAC was determined by a fluorescence-based assay utilizing the intrinsic fluorescence of the PpIX moiety. Mechanistically, the spontaneous micellization significantly alters the local microenvironment (e.g., polarity and spatial restriction) of the porphyrin rings, leading to a sharp transition in emission intensity. Briefly, SNAP-TAC dispersions with varying Pep-PpIX concentrations (0.025 to 20 μM) and a fixed Pep-PpIX/JQ1-CBT molar ratio of 1:2 were prepared. The fluorescence intensities were recorded using a microplate reader (Ex = 410 nm, Em = 630 nm). The CMC value was defined as the inflection point in the plot of emission intensity versus the logarithm of Pep-PpIX concentration.

Hemolysis Assay: 500 μL of SNAP-TAC solutions (0-1000 μM) were mixed with an equal volume of 4% rabbit red blood cell suspension. PBS and 1% Triton X-100 served as negative and positive controls, respectively. After incubation at 37 °C in the dark for 2 h, samples were centrifuged (3000 rpm, 15 min, 4 °C). The absorbance of the supernatant at 540 nm was measured by a microplate reader to calculate the hemolysis ratio.

Extracellular ROS Detection: The generation of reactive oxygen species (ROS) was evaluated using the DCFH-DA probe. First, DCFH-DA was chemically hydrolyzed to DCFH by mixing 0.5 mL of DCFH-DA (1 mM) with 2 mL of NaOH (0.01 M) in the dark for 30 min at room temperature. The hydrolysis was terminated by adding 10 mL of Tris buffer (25 mM, pH 7.2). For ROS measurement, SNAP-TAC nanoparticles were mixed with 20 μL of the DCFH solution in 1 mL of PBS. A control group lacking SNAP-TAC was prepared in parallel. The mixtures were irradiated with a 660 nm laser (196 mW/cm^2) for 0, 1, 2, 3, 4, and 5 min. The fluorescence emission spectra of the oxidized product (DCF) were immediately recorded using a fluorescence spectrophotometer (Ex = 488 nm).

Sequential Cleavage and Single-Stimulus Control Assays: To evaluate the dual-responsive activation mechanism, Pep-PpIX was evaluated under both sequential dual-stimulus and independent single-stimulus conditions. For the sequential dual-stimulus assay, Pep-PpIX (2 mg) was dissolved in 1 mL of CTSB-activation buffer (0.01 M acetate buffer, pH 5.5, 2 mM cysteine, 1 mM EDTA). CTSB (10 μL, 20 U/mL) was added, and the mixture was incubated at

37 °C for 2 h to generate the CTSB-cleaved intermediate (P779). Subsequently, the model reductant DTT (0.5 mg) was added to the mixture. Following further incubation to cleave the disulfide bond, the fully unmasked active intermediate (P665) was generated. To confirm the necessity of the dual stimuli, independent single-stimulus control assays were performed in parallel. Pep-PpIX was incubated separately with either CTSB alone or DTT alone under identical conditions. Treatment with CTSB alone yielded the intermediate P779, whereas treatment with DTT alone generated the disulfide-cleaved but peptide-intact intermediate (P886). All reactions were quenched with acetonitrile and analyzed by HPLC and high-resolution mass spectrometry (HRMS). P779 (CTSB cleavage): HRMS (ESI) *m/z*: Calcd. for $C_{60}H_{101}N_{17}O_{13}S_2$ $[M+H]^+$ 1332.7279, $[M+2H]^{2+}$ 666.8676, found: $[M+H]^+$ 1332.7247, $[M+2H]^{2+}$ 666.8657. P665 (Sequential CTSB and DTT): HRMS (ESI) *m/z*: Calcd. for $C_{56}H_{93}N_{17}O_{13}S$ $[M+H]^+$ 1244.6932, $[M+2H]^{2+}$ 622.8502, found: $[M+H]^+$ 1244.6897, $[M+2H]^{2+}$ 622.8487. P886 (DTT alone): HRMS (ESI) *m/z*: Calcd. for $C_{180}H_{282}N_{52}O_{34}S_2$ (most abundant isotope) $[M+3H]^{3+}$ 1261.7221, $[M+4H]^{4+}$ 946.5434, $[M+5H]^{5+}$ 757.4362, found: $[M+3H]^{3+}$ 1261.7218, $[M+4H]^{4+}$ 946.5428, $[M+5H]^{5+}$ 757.4359.

Kinetics of CBT-Cys Click Condensation: To evaluate the bioorthogonal click reaction kinetics, a simplified model peptide PC4 was utilized. Briefly, PC4 (10 μL, 100 μM), JQ1-CBT (10 μL, 100 μM), and TCEP (10 μL, 100 μM) were uniformly mixed in 300 μL of 50% acetonitrile solution (v/v). The mixture was continuously incubated on a shaker at 37 °C. Aliquots were collected at predetermined time points (0, 0.5, 2, 12, and 24 h) and directly analyzed by HPLC and MS to monitor the consumption of precursors and the formation of the click product JPC4. PC4: HRMS (ESI) *m/z*: calcd. for $C_{44}H_{70}N_{10}O_{10}S$ $[M+H]^+$ 931.5070, $[M+Na]^+$ 953.4895, found: $[M+H]^+$ 931.5042, $[M+Na]^+$ 953.4856. JPC4: HRMS (ESI) *m/z*: Calcd. For $C_{71}H_{86}ClN_{15}O_{12}S_3$ $[M+H]^+$ 1472.5504, $[M+2H]^{2+}$ 736.7788, found: $[M+H]^+$ 1472.5465, $[M+2H]^{2+}$ 736.7777.

Cellular Uptake Assay: The intracellular uptake of SNAP-TAC was evaluated using confocal laser scanning microscopy (CLSM). 4T1 cells were seeded in confocal dishes at a density of 1.5×10^5 cells/dish and incubated for 24 h. For the time-dependent assay, cells were treated with SNAP-TAC (10 μM) for 0, 1, 2, and 3 h. For the concentration-dependent assay, cells were incubated with varying concentrations of SNAP-TAC (0, 2.5, 5, and 10 μM) for 3 h. Following incubation, the cells were washed with PBS to remove free nanoparticles and imaged directly by CLSM utilizing the intrinsic fluorescence of the SNAP-TAC. The intracellular fluorescence intensities were quantified using ImageJ software.

***In Vitro* Cytotoxicity Assay:** Cell viabilities of 4T1 tumor cells and normal 3T3 fibroblasts

were evaluated using the CCK-8 assay. Cells were seeded in 96-well plates (3,000 cells/well) and cultured for 24 h. Subsequently, cells were treated with varying concentrations (0, 0.625, 1.25, 2.5, 5, and 10 μM) of Pep-PpIX, Pep-PpIX+JQ1, JQ1-CBT, or SNAP-TAC. For biosafety evaluation, 3T3 cells were continuously incubated in the dark for 48 h. For synergistic efficacy evaluation, 4T1 cells were incubated for 24 h; the designated photo-irradiation groups (Pep-PpIX(+) and SNAP-TAC(+)) were then exposed to a 660 nm laser (196 mW/cm^2) for 2 min, followed by an additional 24 h of incubation. Finally, a mixture of CCK-8 reagent (10 μL) and DMEM (100 μL) was added to each well. After 1 h of incubation at 37 °C, the absorbance at 450 nm was measured using a microplate reader.

Intracellular ROS Detection: 4T1 cells (8 × 10^4/dish) were pre-incubated for 24 h, divided into five groups (PBS, Pep-PpIX, SNAP-TAC, Pep-PpIX(+), and SNAP-TAC(+)), and treated respectively for 24 h. Cells were then stained with 1 mL of DCFH-DA (10 μM, serum-free) for 30 min at 37 °C in the dark. After PBS washing, the (+) groups were irradiated as described above. DCF fluorescence was immediately captured by CLSM and quantified via ImageJ.

Live/Dead Cell Staining: 4T1 cells (8 × 10^4/dish) were pre-incubated for 24 h, divided into five groups (PBS, Pep-PpIX, SNAP-TAC, Pep-PpIX(+), and SNAP-TAC(+)), and treated respectively for 24 h. The (+) groups were irradiated as described above, and all groups were cultured for an additional 24 h. Cells were co-stained with Calcein-AM and PI for 30 min at 37 °C in the dark, washed with PBS, and directly imaged via CLSM.

Apoptosis Analysis by Flow Cytometry: 4T1 cells (2 × 10^5/well) were pre-incubated in 6-well plates for 24 h, divided into five groups (PBS, Pep-PpIX, SNAP-TAC, Pep-PpIX(+), and SNAP-TAC(+)), and treated respectively for 24 h. The (+) groups were irradiated as described above, and all groups were cultured for an additional 24 h. All cells were then harvested, co-stained with Annexin V-FITC and PI for 15 min at room temperature in the dark, and immediately analyzed via flow cytometry.

Western Blot Analysis: 4T1 cells (2 × 10^5/well) pre-incubated in 6-well plates for 24 h were subjected to SNAP-TAC treatments at varying concentrations (0-10 μM) or times (0-48 h), or assigned to designated group treatments (PBS, Pep-PpIX, Pep-PpIX+JQ1, JQ1-CBT, SNAP-TAC+MG132, and SNAP-TAC). Total proteins were extracted using RIPA buffer, quantified via a BCA kit, and denatured at 100 °C. Proteins were separated by SDS-PAGE, transferred to PVDF membranes, and blocked with 5% skim milk. Membranes were then incubated with primary antibodies (anti-BRD4, anti-PD-L1, or anti-GAPDH) at 4 °C overnight, followed by appropriate secondary antibodies for 1 h at room temperature. Protein bands were visualized using an ECL substrate.

Immunogenic Cell Death (ICD) Evaluation: Cell seeding, treatments, and laser irradiation protocols were identical to those described for the apoptosis analysis. For immunofluorescence, cells were immunostained with specific primary antibodies (anti-CRT or anti-HMGB1) and fluorophore-conjugated secondary antibodies, then imaged via CLSM. For quantitative analysis, the intracellular levels of ATP and HMGB1 in cell lysates were determined using ELISA kits according to the manufacturers' protocols.

***In Vitro* Dendritic Cell (DC) Maturation Assay:** The 4T1 cell treatment and laser irradiation procedures were identical to those described in the apoptosis analysis. After 24 h of post-irradiation culture, the supernatants from each group were collected. Immature bone marrow-derived dendritic cells (BMDCs) were seeded in 6-well plates and co-incubated with the collected supernatants for 24 h. Subsequently, the BMDCs were harvested, stained with fluorophore-conjugated anti-CD80 and anti-CD86 antibodies at 4 °C in the dark for 30 min. After PBS washing, the maturation of DCs was analyzed via flow cytometry.

RNA Sequencing and Transcriptomic Analysis: RNA Extraction and Library Preparation. Total RNA was extracted from cultured 4T1 cells using TRIzol® Reagent according to the manufacturer's instructions. RNA quality was determined by a 5300 Bioanalyser (Agilent) and quantified using the ND-2000 (NanoDrop Technologies). Only high-quality RNA samples were used to construct the sequencing library. The RNA-seq transcriptome library was prepared following the Illumina® Stranded mRNA Prep Ligation protocol. Briefly, messenger RNA was isolated according to the polyA selection method by oligo(dT) beads and then fragmented by fragmentation buffer. Double-stranded cDNA was synthesized, subjected to end-repair, phosphorylation, and 'A' base addition, and then PCR amplified. After quantification by Qubit 4.0, the paired-end RNA-seq sequencing library was sequenced with the NovaSeq X plus sequencer (2 × 150bp read length). Data Processing and Differential Expression Analysis: The raw paired-end reads were trimmed and quality controlled by fastp with default parameters. Clean reads were then separately aligned to the reference genome using HISAT2 software. The mapped reads of each sample were assembled by StringTie. To quantify gene abundances, the expression level of each transcript was calculated according to the transcripts per million reads (TPM) method using RSEM. Differential expression analysis was performed using DESeq2. DEGs with $|\log2FC| \geqq 1$ and $FDR < 0.05$ were considered to be significantly differentially expressed genes. GO functional enrichment and KEGG pathway analysis were carried out by Goatools and Python scipy software, respectively, on the online platform of Majorbio Cloud Platform.

Animals and Tumor Model Establishment: Female BALB/c mice (6-7 weeks old, 16-18 g,

Huachuang Xinnuo Pharmaceutical Technology Co., Ltd., Jiangsu, China) were utilized in compliance with the protocols approved by the Ethics Committee of Shanghai University (Approval No. ECSHU 2025-122). The animals were housed under specific pathogen-free (SPF) conditions with a 12-hour light/dark cycle, controlled temperature and humidity, and ad libitum access to standard rodent chow and water. To establish the subcutaneous tumor model, 1×10^6 4T1 cells were injected into the right hind limb of each mouse. Seven days post-inoculation, the mice were randomly assigned to designated experimental groups (n = 5) for subsequent treatments. During the study, strict humane endpoints were established; mice would be euthanized if the tumor volume exceeded 1500 mm^3, or if they experienced severe body weight loss (> 20%).

In Vivo Biodistribution and Imaging: 4T1 tumor-bearing mice were randomly divided into three groups and intravenously injected with PBS, free PpIX, or SNAP-TAC (equivalent PpIX dose: 3 mg/kg). Real-time fluorescence signals were monitored at 2, 4, 8, 12, 24, and 48 h post-injection using an IVIS Spectrum imaging system (PerkinElmer, USA). To collect the long-wavelength emission of PpIX while minimizing tissue autofluorescence, the Cy5.5 channel was utilized for imaging. At 48 h post-injection, the mice were euthanized; major organs (heart, liver, spleen, lung, and kidneys) and tumors were harvested for *ex vivo* fluorescence imaging and quantitative analysis. For image visualization and comparison, fluorescence images were processed using Living Image software with Rainbow pseudocolor mapping. A standardized manual scale was applied (minimum threshold: 2.50×10^7; maximum threshold: 5.50×10^7 [p/s/cm²/sr] / [μW/cm²]) to filter out low-level background noise.

***In Vivo* Antitumor Efficacy and Biosafety Evaluation:** 4T1 tumor-bearing mice were randomly assigned to five groups (PBS, Pep-PpIX, SNAP-TAC, Pep-PpIX(+), and SNAP-TAC(+)) and intravenously injected with respective formulations (equivalent PpIX dose: 2.76 mg/kg) every 3 days for 5 cycles. At 24 h post-injection, tumors in the (+) groups were irradiated (660 nm, 196 mW/cm^2, 5 min). Body weights and tumor volumes ($V = L \times W^2/2$, where L and W are the long and short diameters) were monitored every 3 days. On day 15, mice were euthanized to collect blood, major organs (heart, liver, spleen, lungs, and kidneys), and tumors. Excised tumors were weighed and processed for histological (H&E, TUNEL, Ki-67) and immunofluorescence (ROS, BRD4, PD-L1) analyses. Major organs were H&E-stained for histopathological evaluation, while blood samples underwent complete blood count (CBC) and serum biochemical (ALT, AST, ALP, Urea, Crea) analyses to comprehensively assess systemic biosafety.

Flow Cytometry Analysis of the Tumor Immune Microenvironment: Upon completion of

the treatment regimen, excised tumors and tumor-draining lymph nodes (dLNs) were processed to yield single-cell suspensions. Specifically, solid tumor tissues underwent a 2-hour enzymatic dissociation at 37 °C using a DMEM mixture supplemented with collagenase and DNase, whereas dLNs were disrupted mechanically. Following erythrocyte depletion via lysis buffer, the samples were subjected to Fc receptor blocking and viability staining to exclude dead cells. Cells were subsequently labeled with specific fluorochrome-conjugated extracellular antibodies (4 °C, 30 min, in the dark). For Treg profiling, intracellular FOXP3 staining was performed post-fixation and permeabilization. Flow cytometric acquisition was evaluated via FlowJo software.

Statistical Analysis: GraphPad Prism software was used for statistical analysis. Results are reported as the mean ± SD from independent experiments ($n \geq 3$). The statistical disparities between two experimental groups were analyzed using an unpaired Student's t-test. Variations among multiple groups were assessed via one-way or two-way analysis of variance (ANOVA), followed by a Tukey's multiple comparisons test, depending on the number of independent variables. Differences were considered statistically significant when $P < 0.05$, denoted as * $P < 0.05$, ** $P < 0.01$, *** $P < 0.001$, and **** $P < 0.0001$ ("ns" stands for not significant).

Supporting Figures

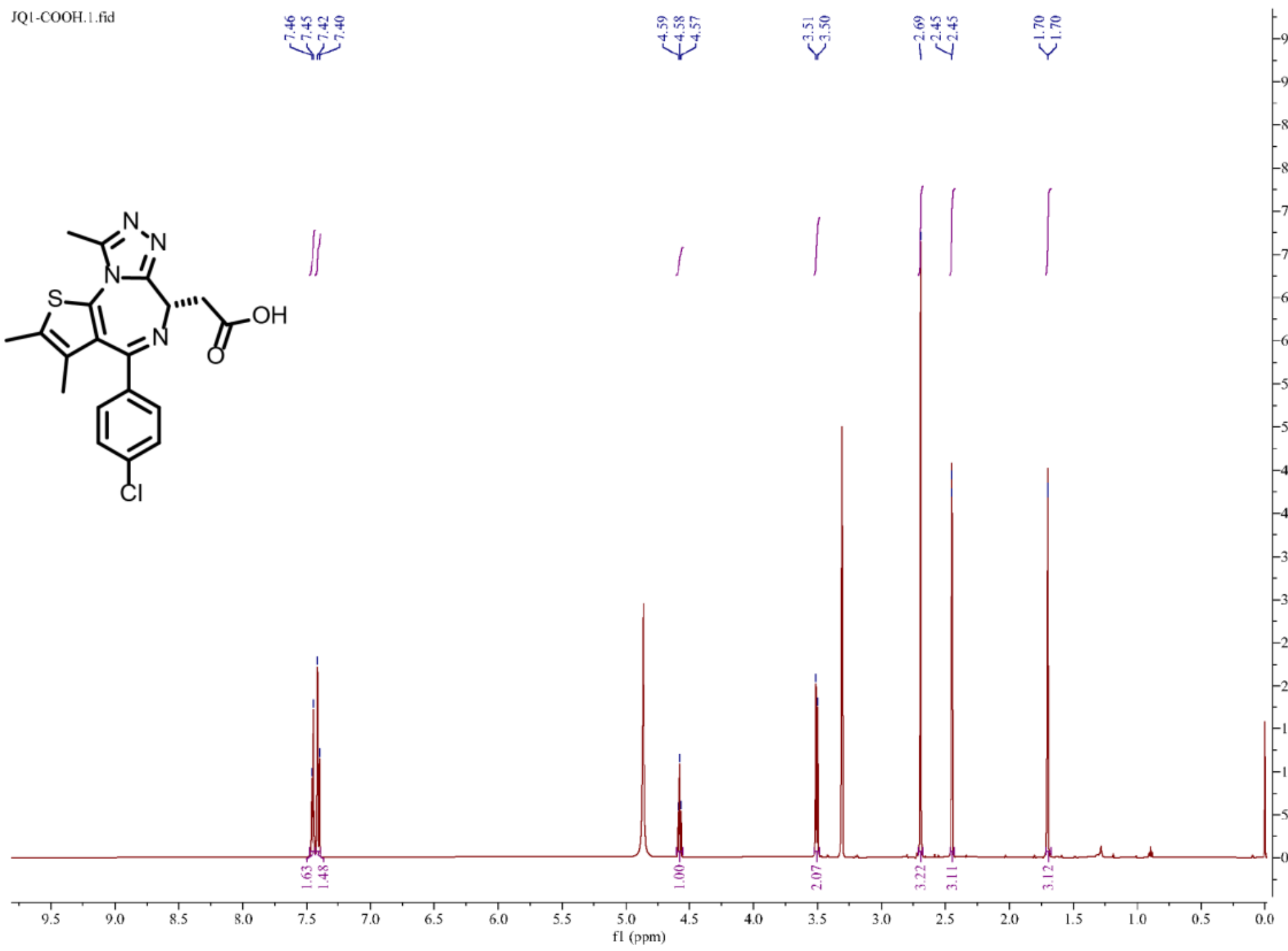

Figure S1. [1] H NMR spectrum of compound JQ1-COOH in CD_3OD (600 MHz).

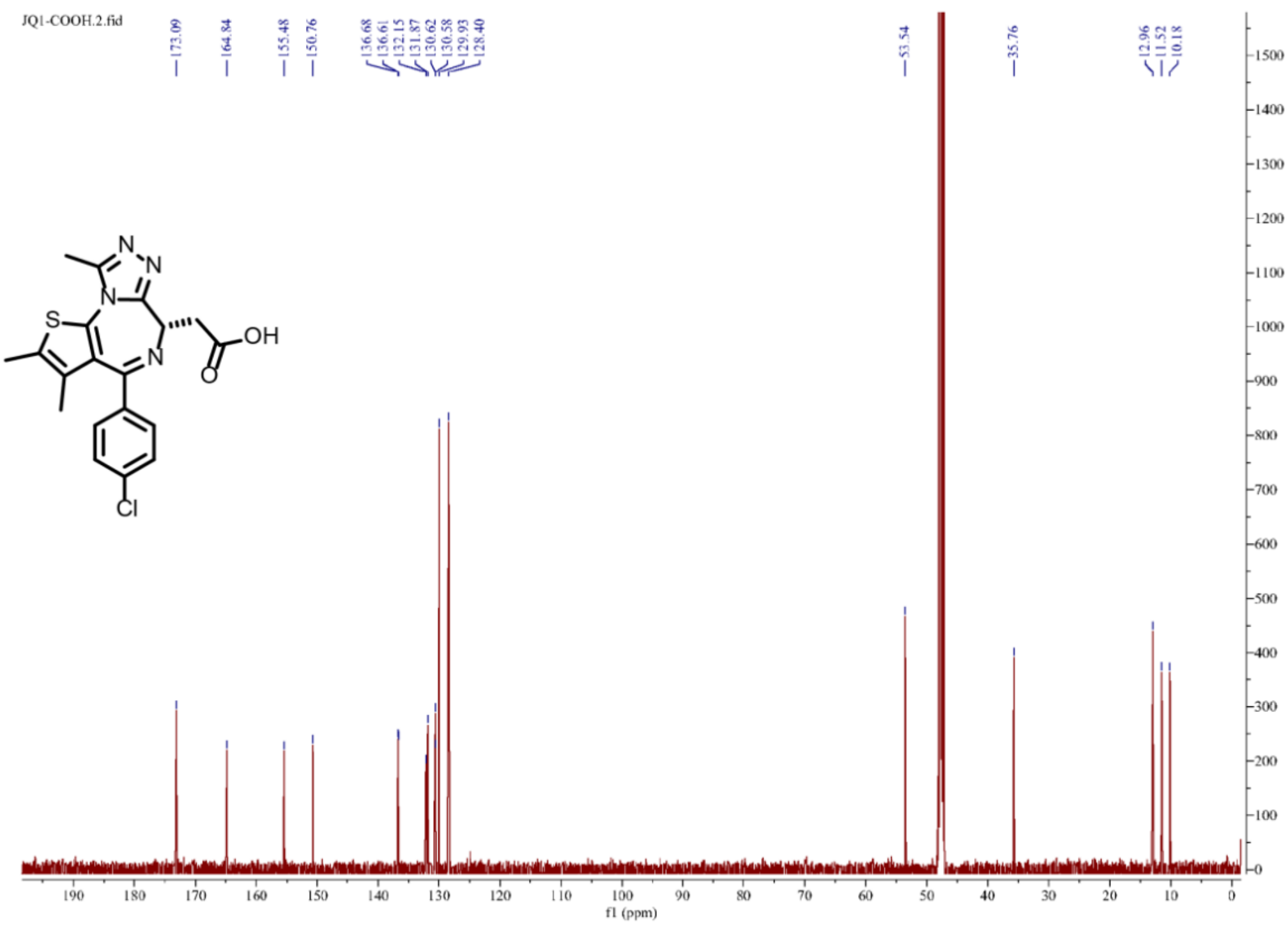

Figure S2. ^{13}C NMR spectrum of compound JQ1-COOH in CD_3OD (151 MHz).

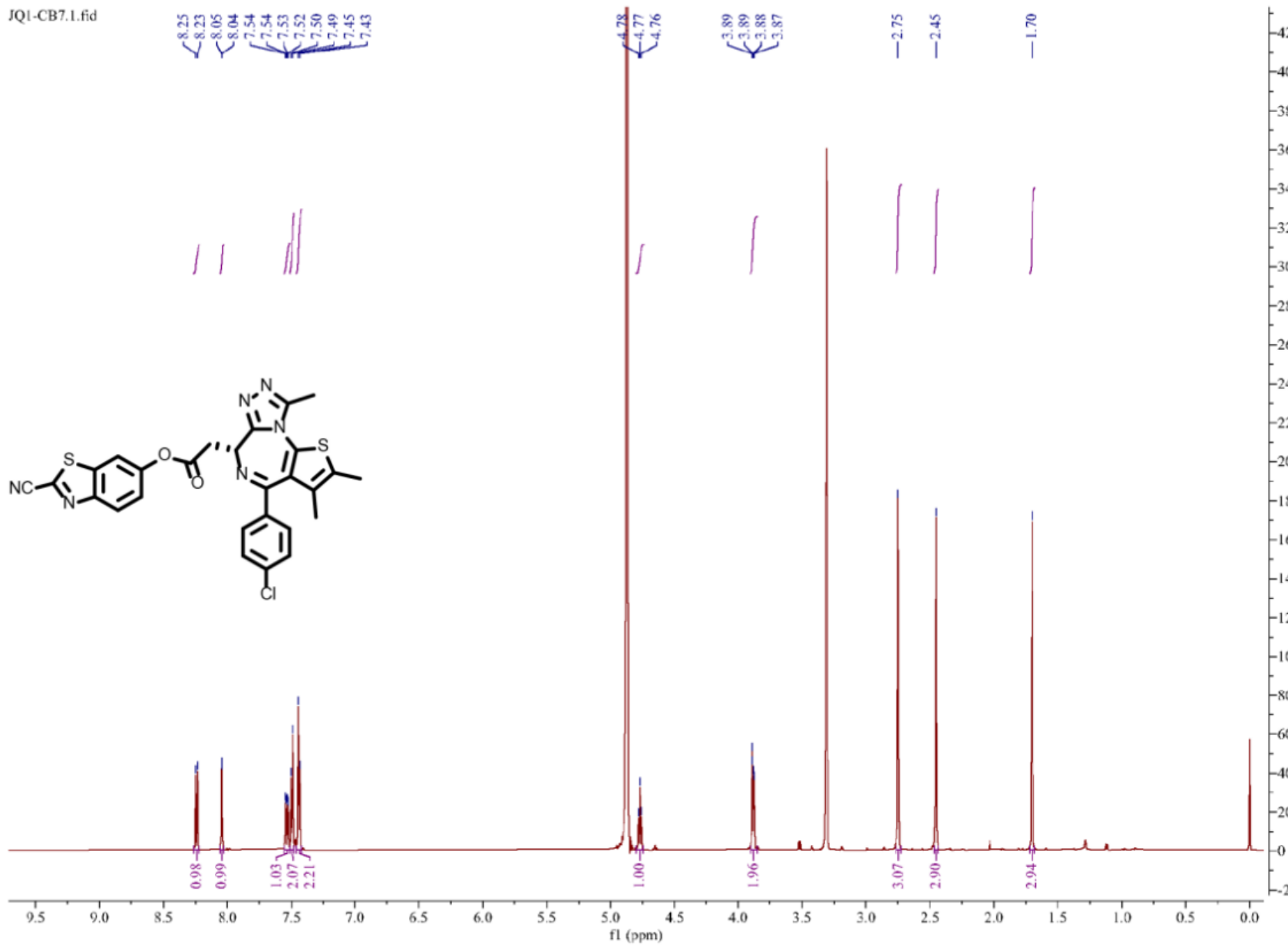

Figure S3. ^{1}H NMR spectrum of compound JQ1-CBT in CD_3OD (600 MHz).

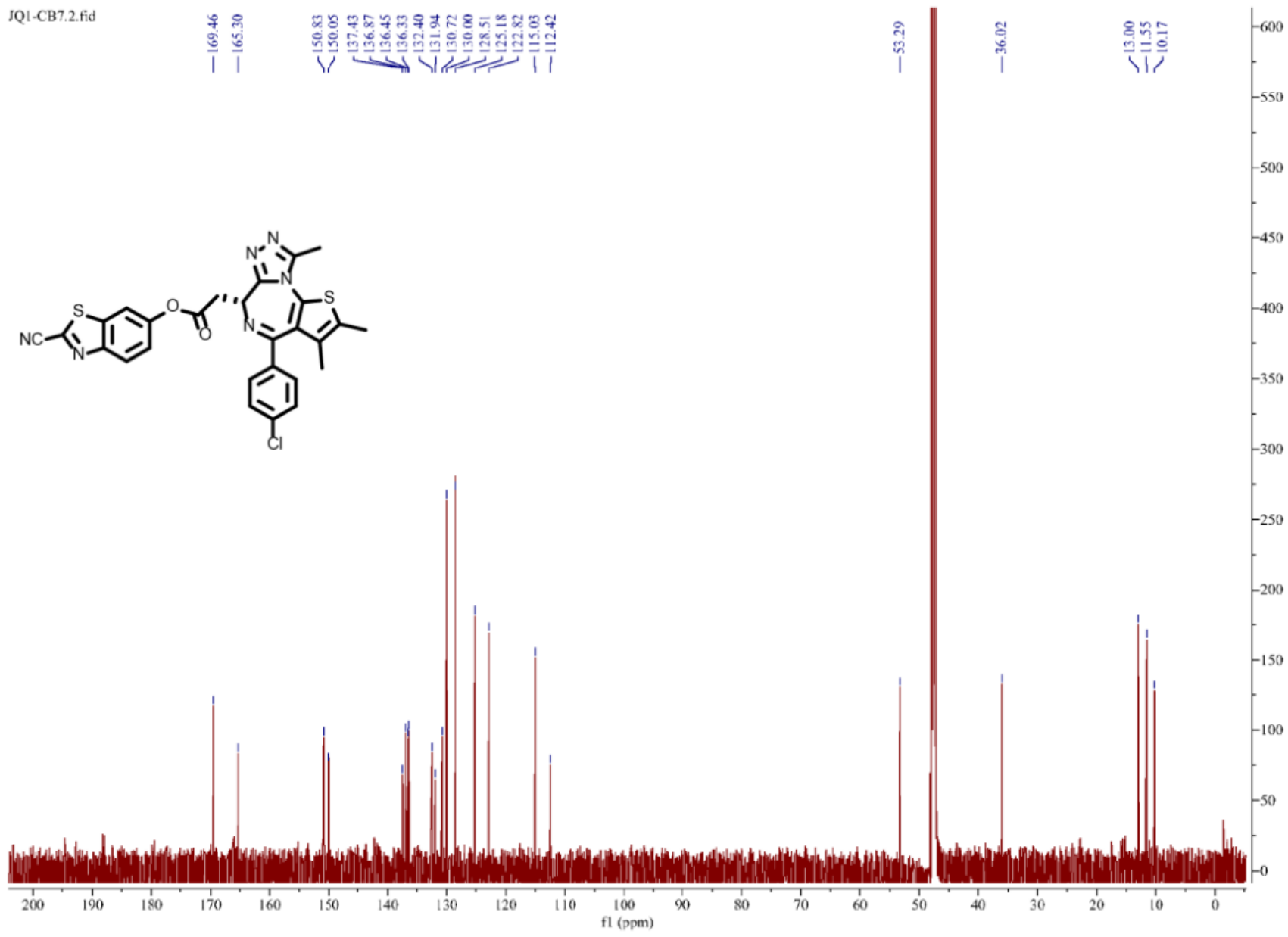

Figure S4. ^{13}C NMR spectrum of compound JQ1-CBT in CD_3OD (151 MHz).

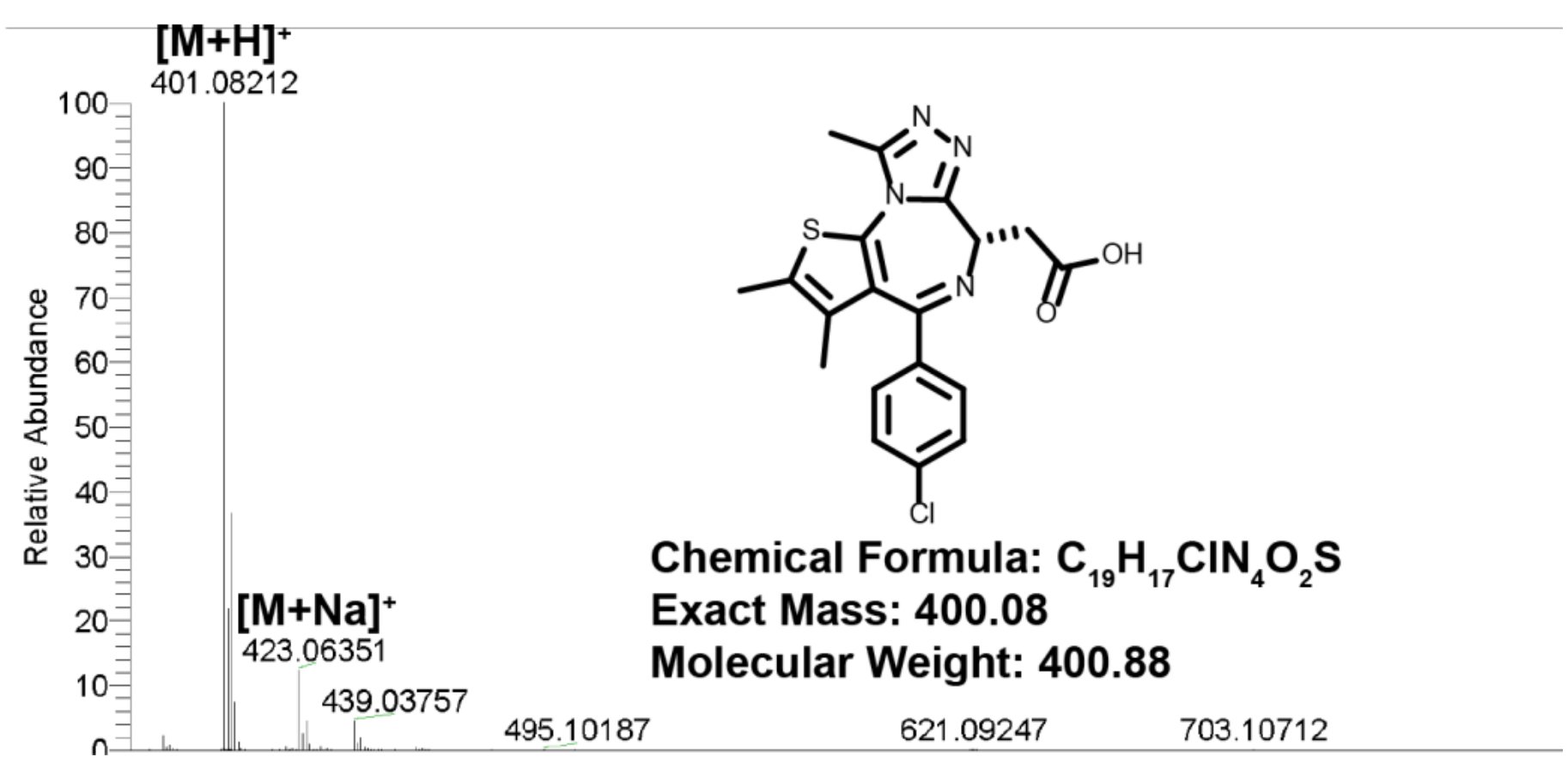

Figure S5. The mass spectrometry of JQ1-COOH.

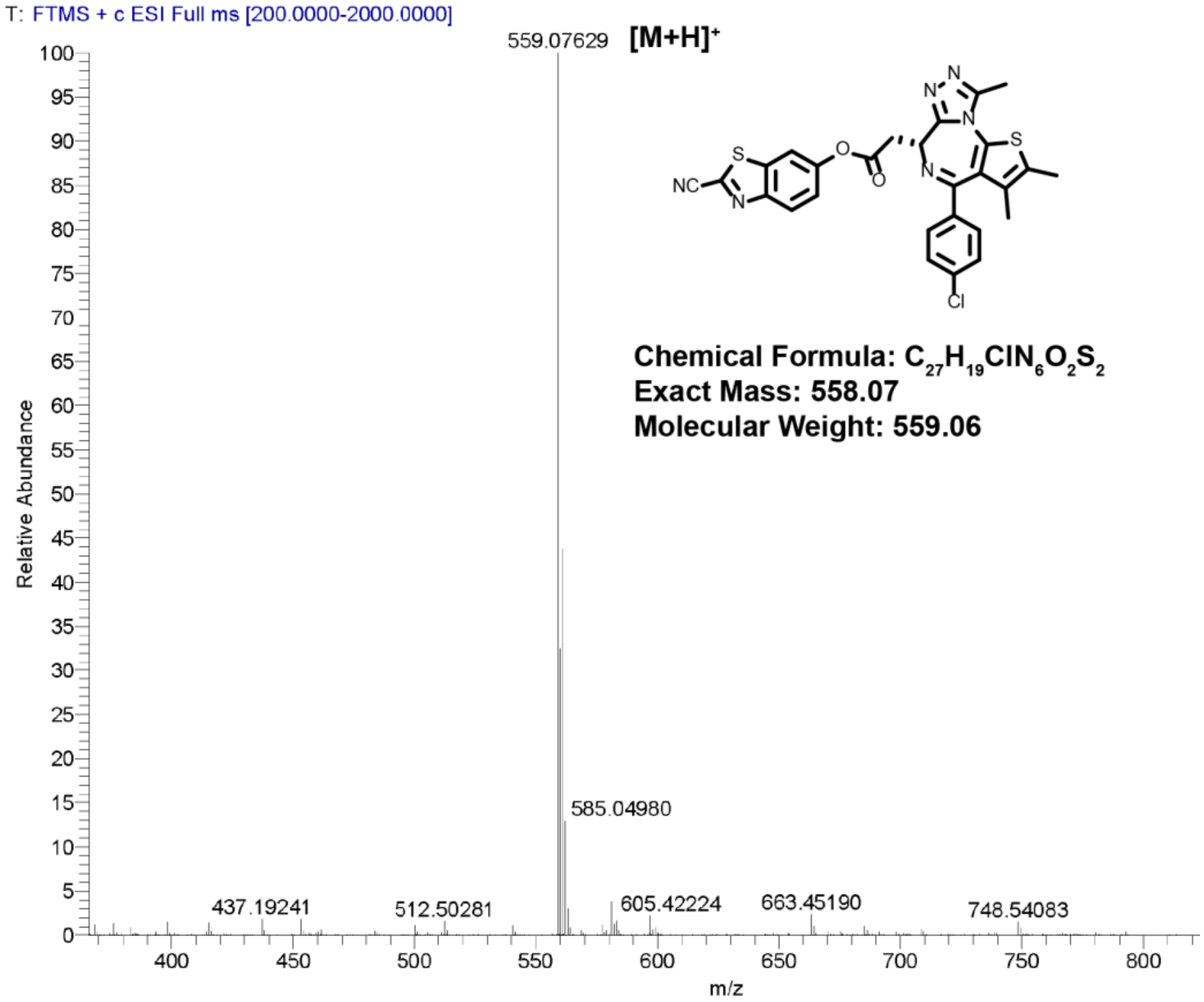

Figure S6. The mass spectrometry of JQ1-CBT.

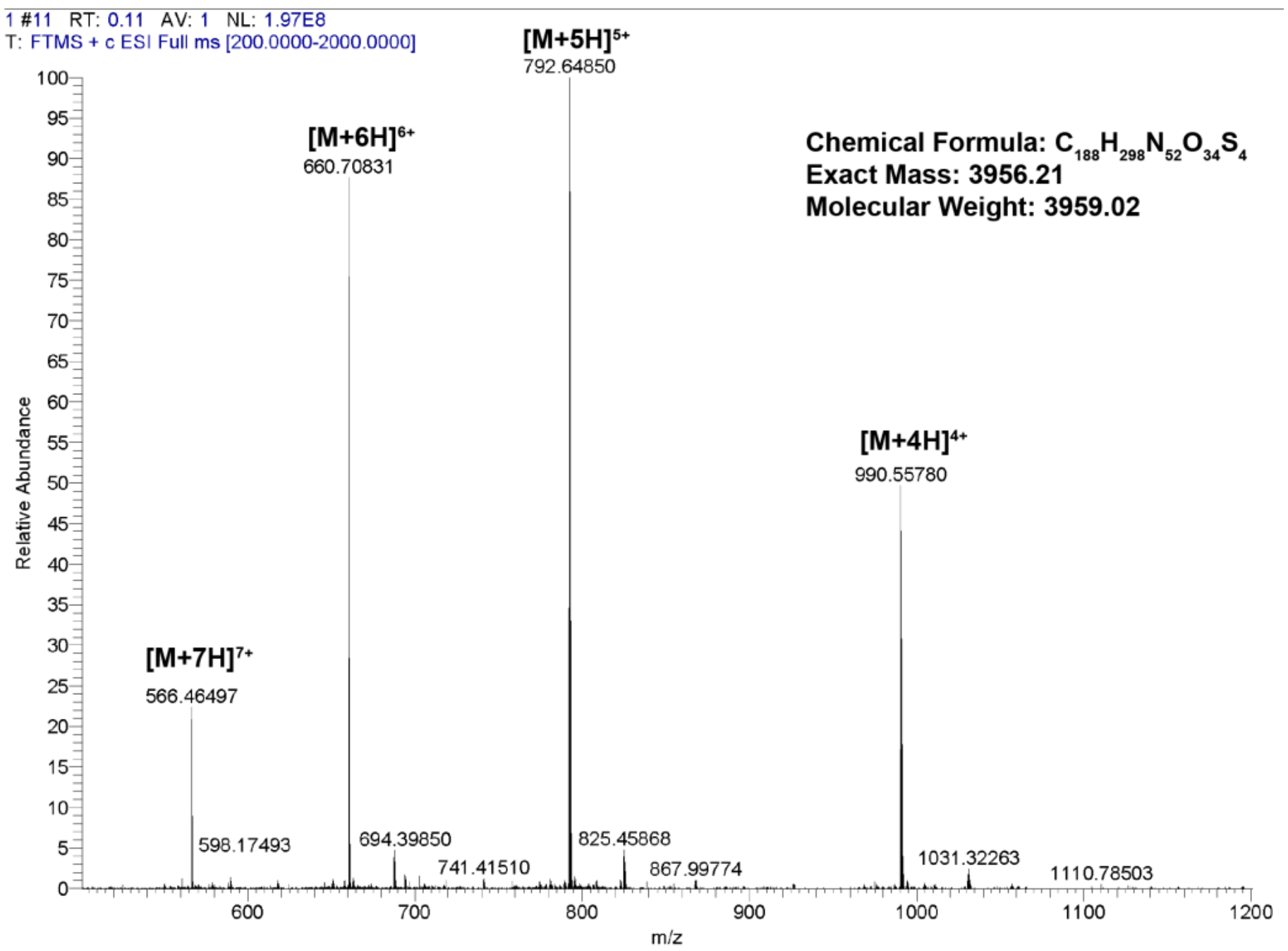

Figure S7. The mass spectrometry of Pep-PpIX.

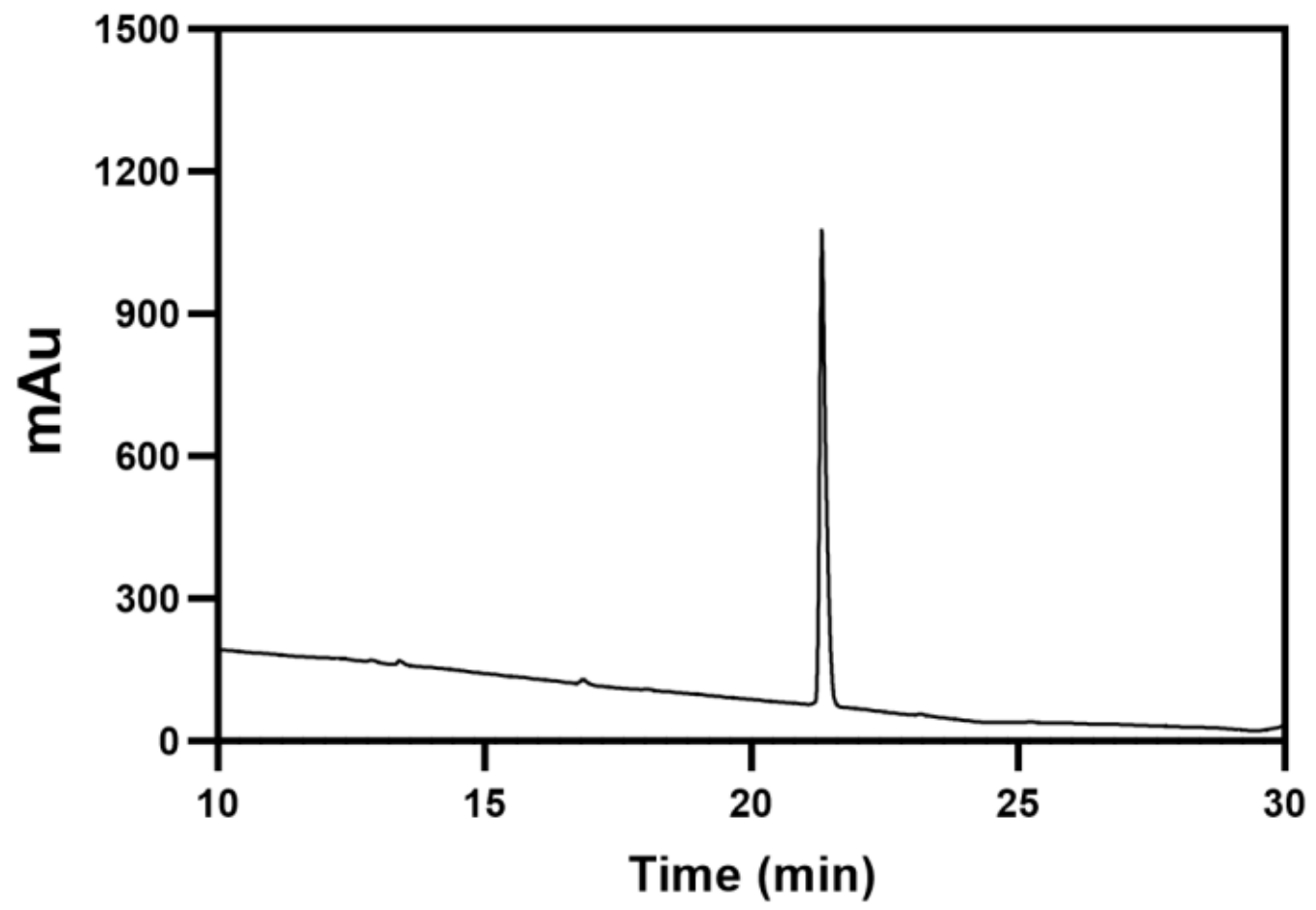

Figure S8. Analytical HPLC chromatogram of JQ1-CBT. The purity is >95% (retention time = 21.32 min). Detection wavelength: 214 nm.

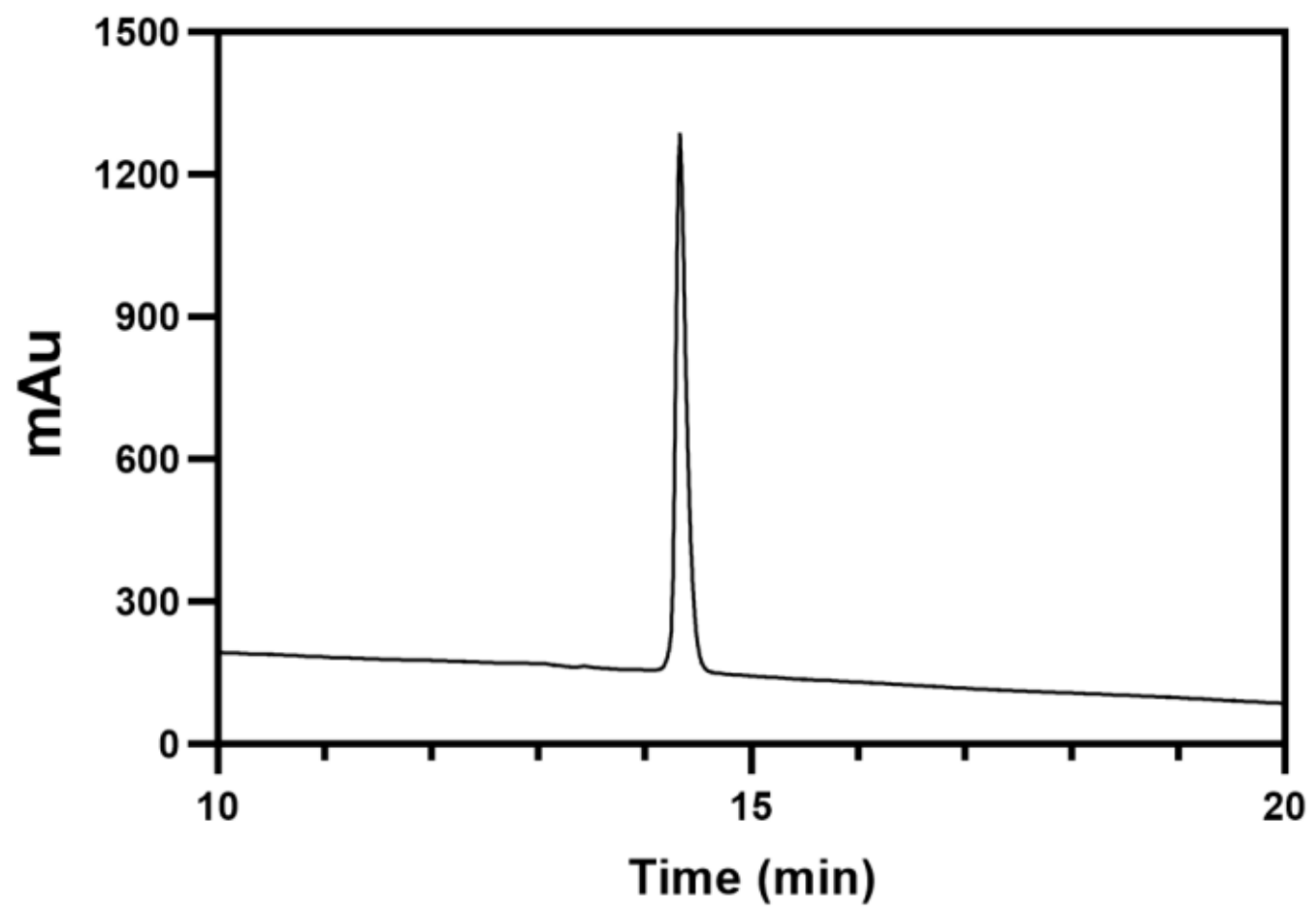

Figure S9. Analytical HPLC chromatogram of Pep-PpIX. The purity is >95% (retention time = 14.35 min). Detection wavelength: 214 nm.

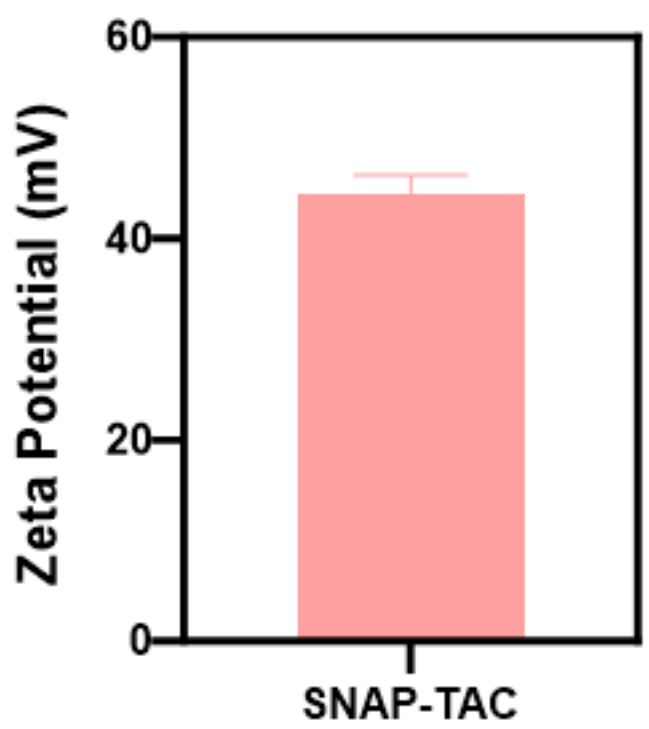

Figure S10. The Zeta Potential of SNAP-TAC. Data are presented as mean ± SD (*n* = 3).

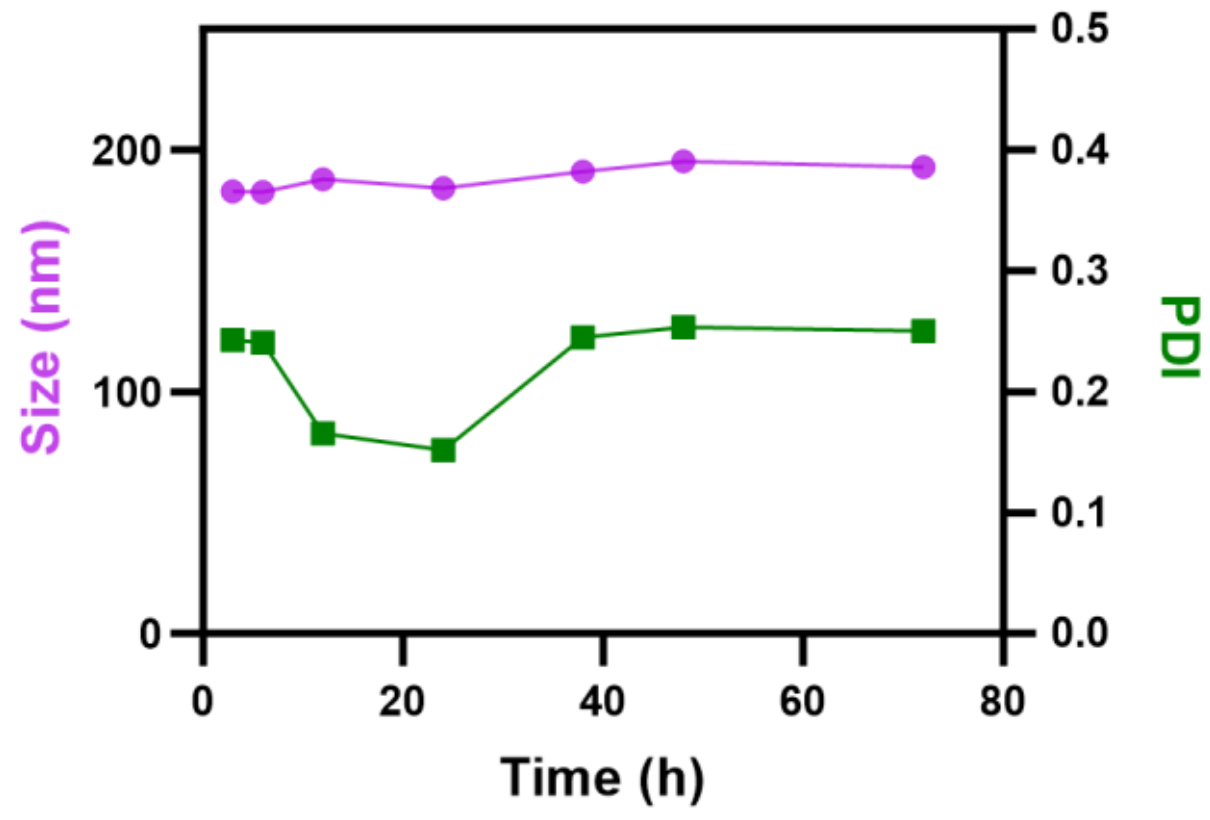

Figure S11. Colloidal stability of SNAP-TAC. Time-dependent changes in hydrodynamic size (purple) and polydispersity index (PDI, green) of SNAP-TAC during incubation in PBS containing 10% FBS at 37 °C for 72 h.

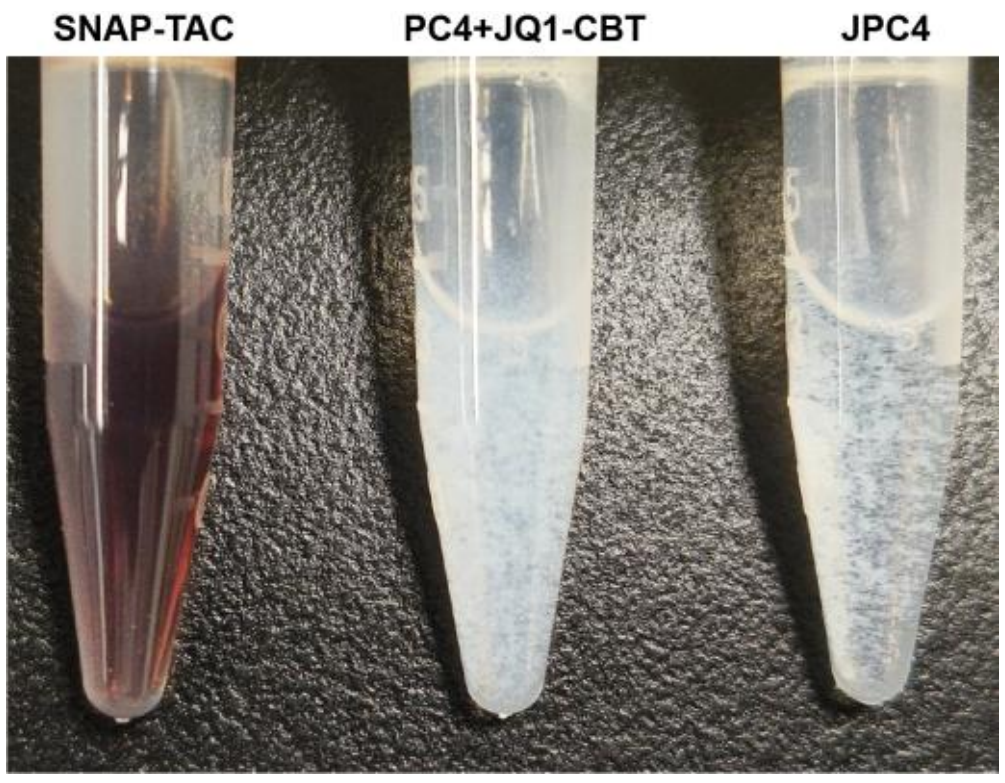

Figure S12. Aqueous solubility and dispersibility comparison.

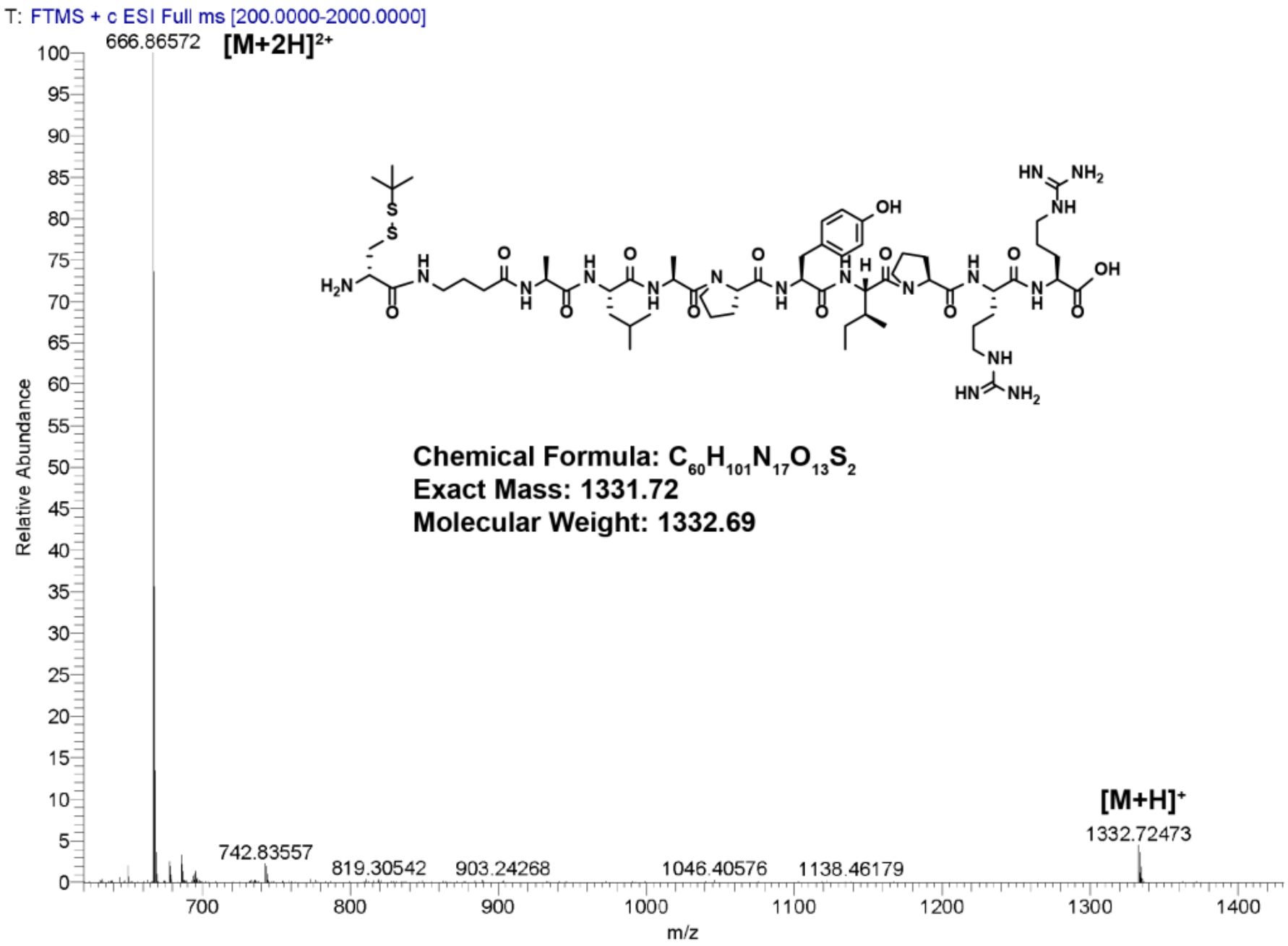

Figure S13. The mass spectrometry of P779.

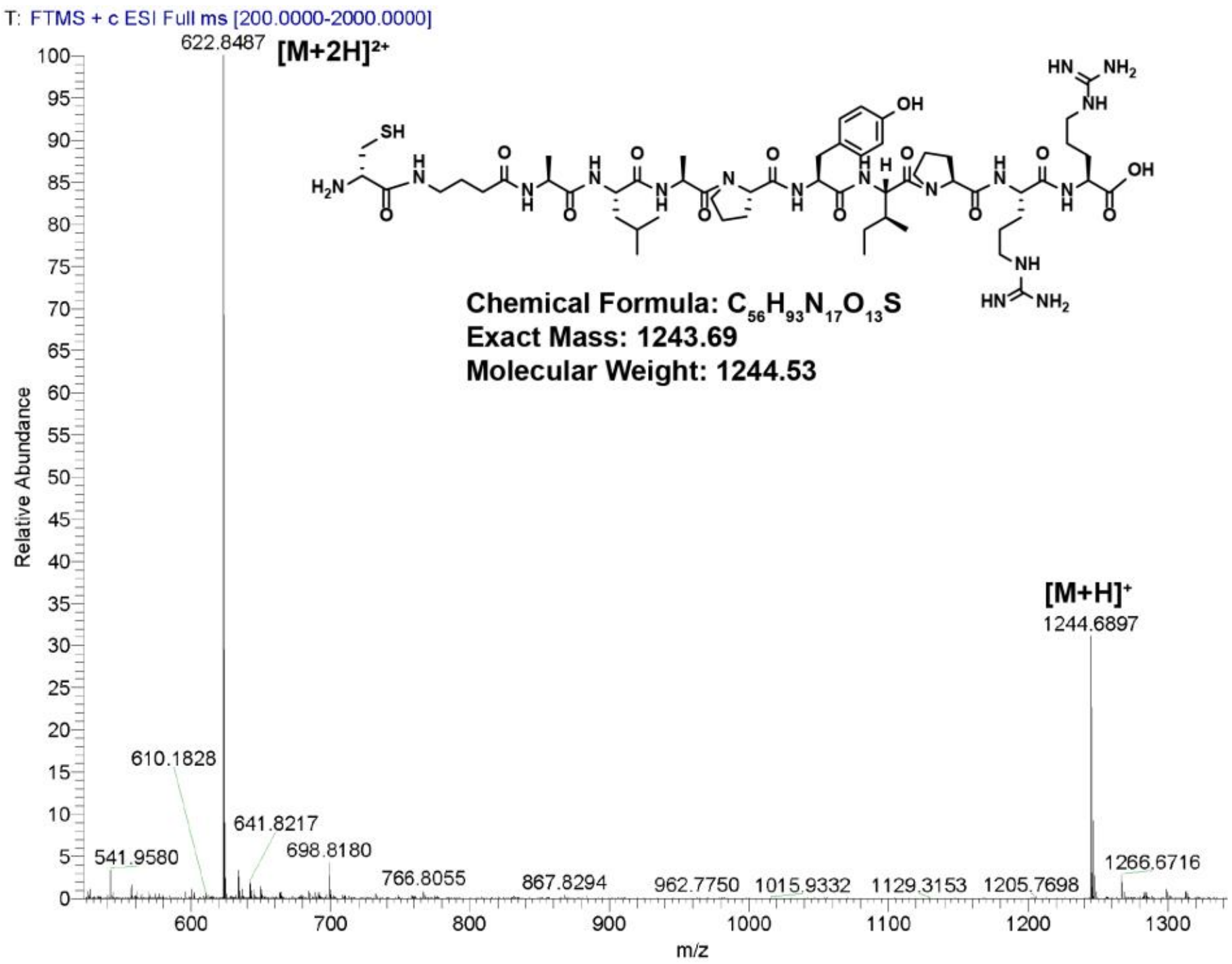

Figure S14. The mass spectrometry of P665.

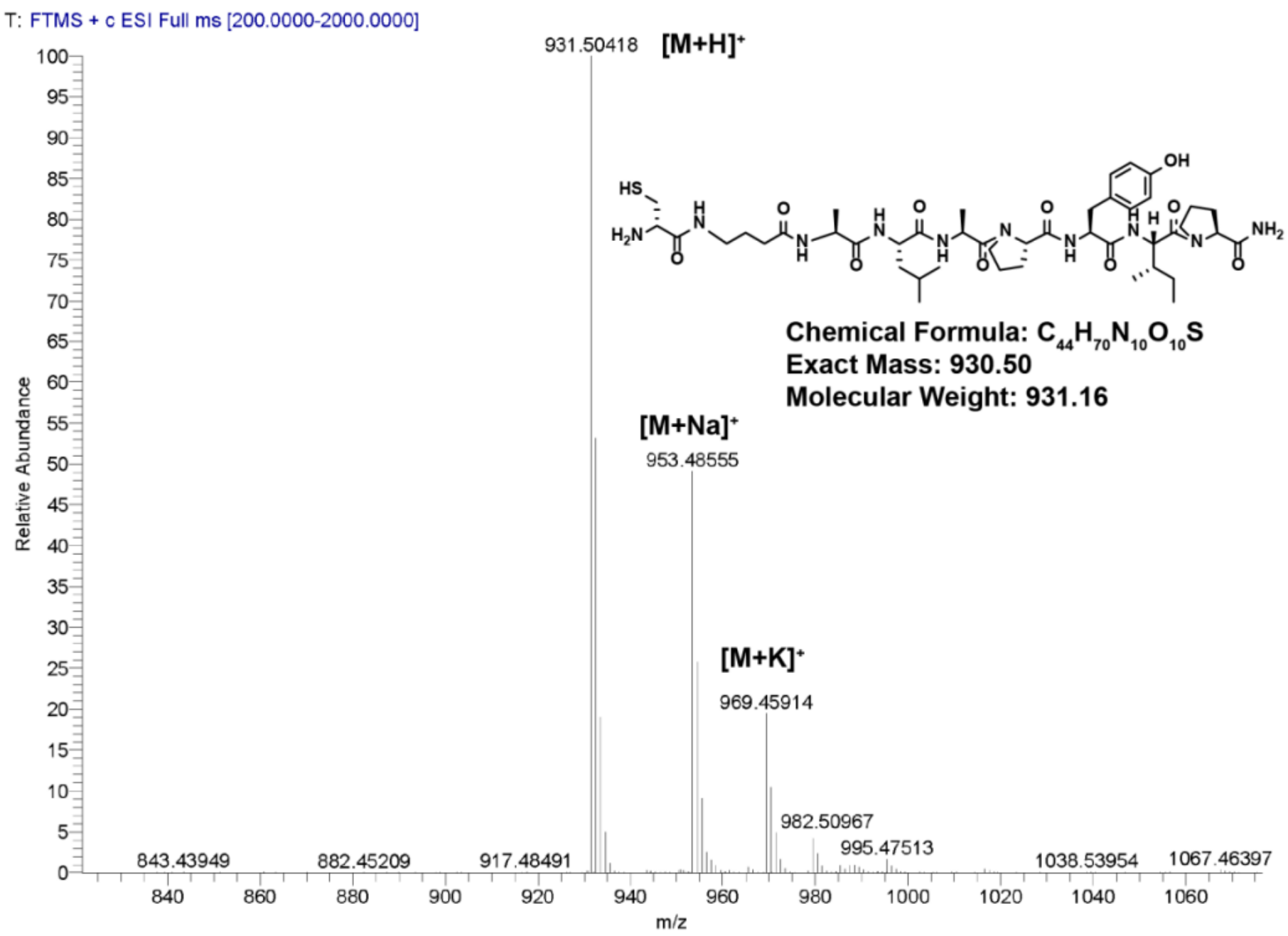

Figure S15. The mass spectrometry of PC4.

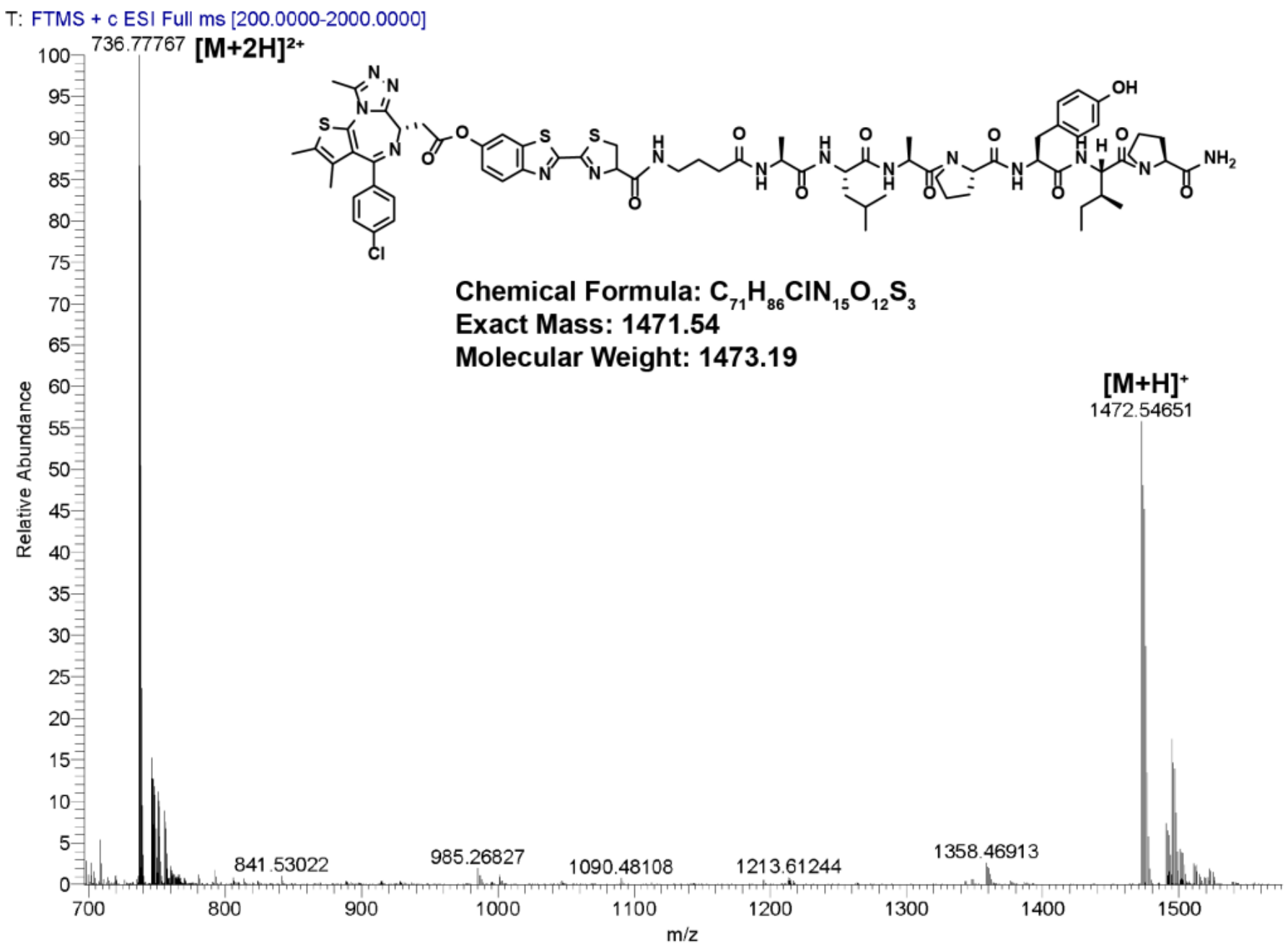

Figure S16. The mass spectrometry of JPC4.

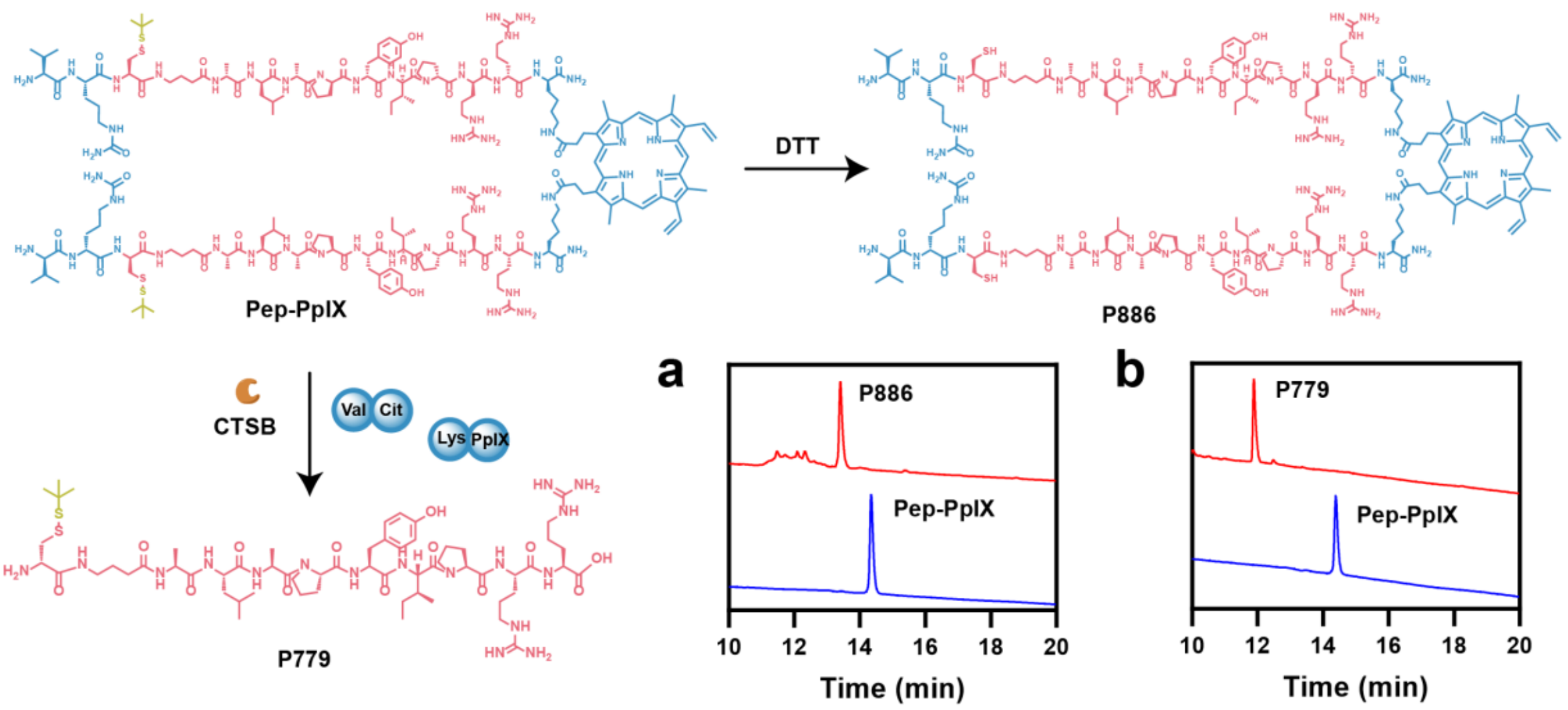

Figure S17. Single-stimulus control HPLC analyses of Pep-PpIX. (a) HPLC chromatograms of Pep-PpIX before and after treatment with DTT alone (yielding P886). (b) HPLC chromatograms of Pep-PpIX before and after treatment with CTSB alone (yielding P779).

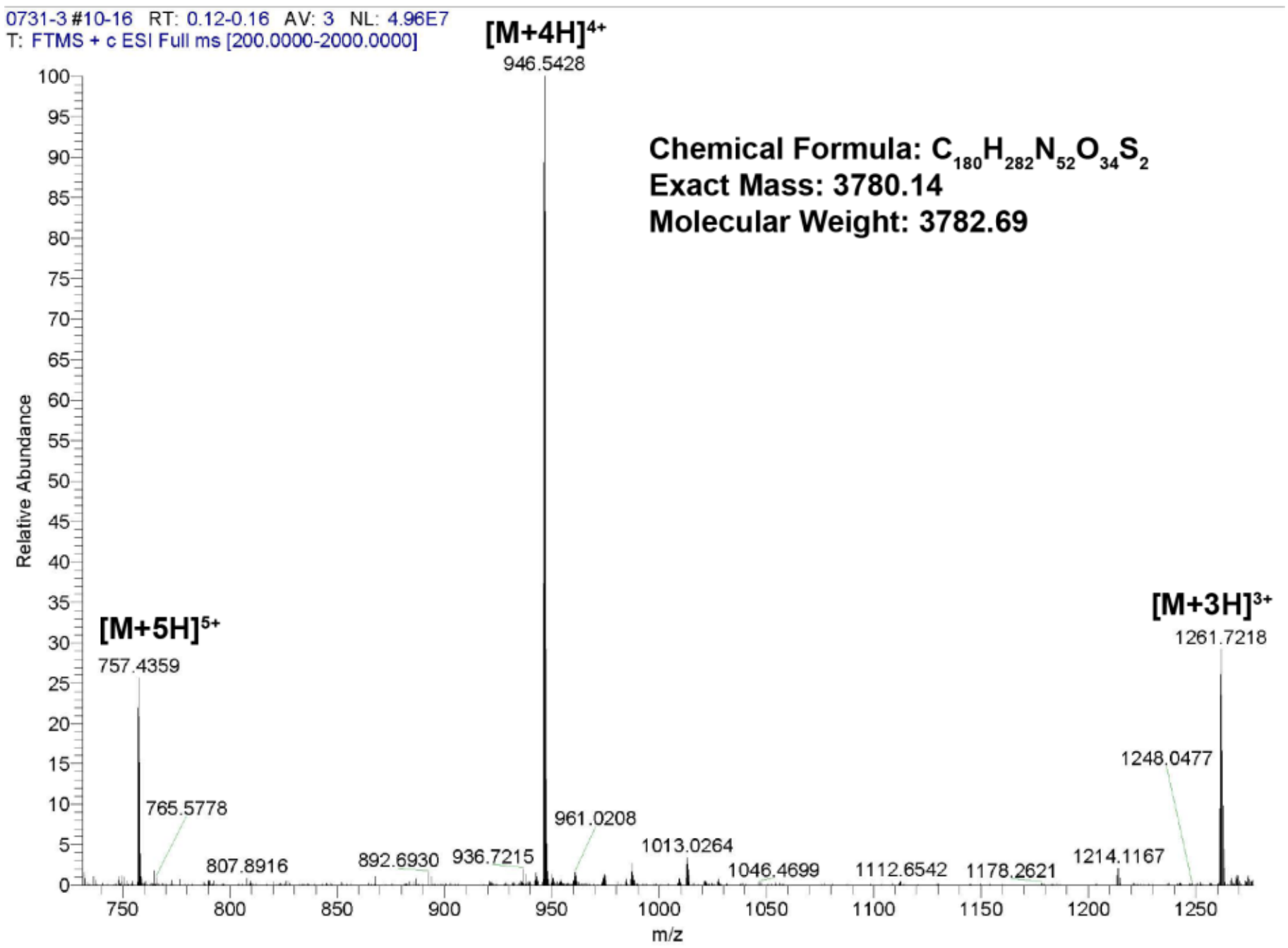

Figure S18. The mass spectrometry of P886.

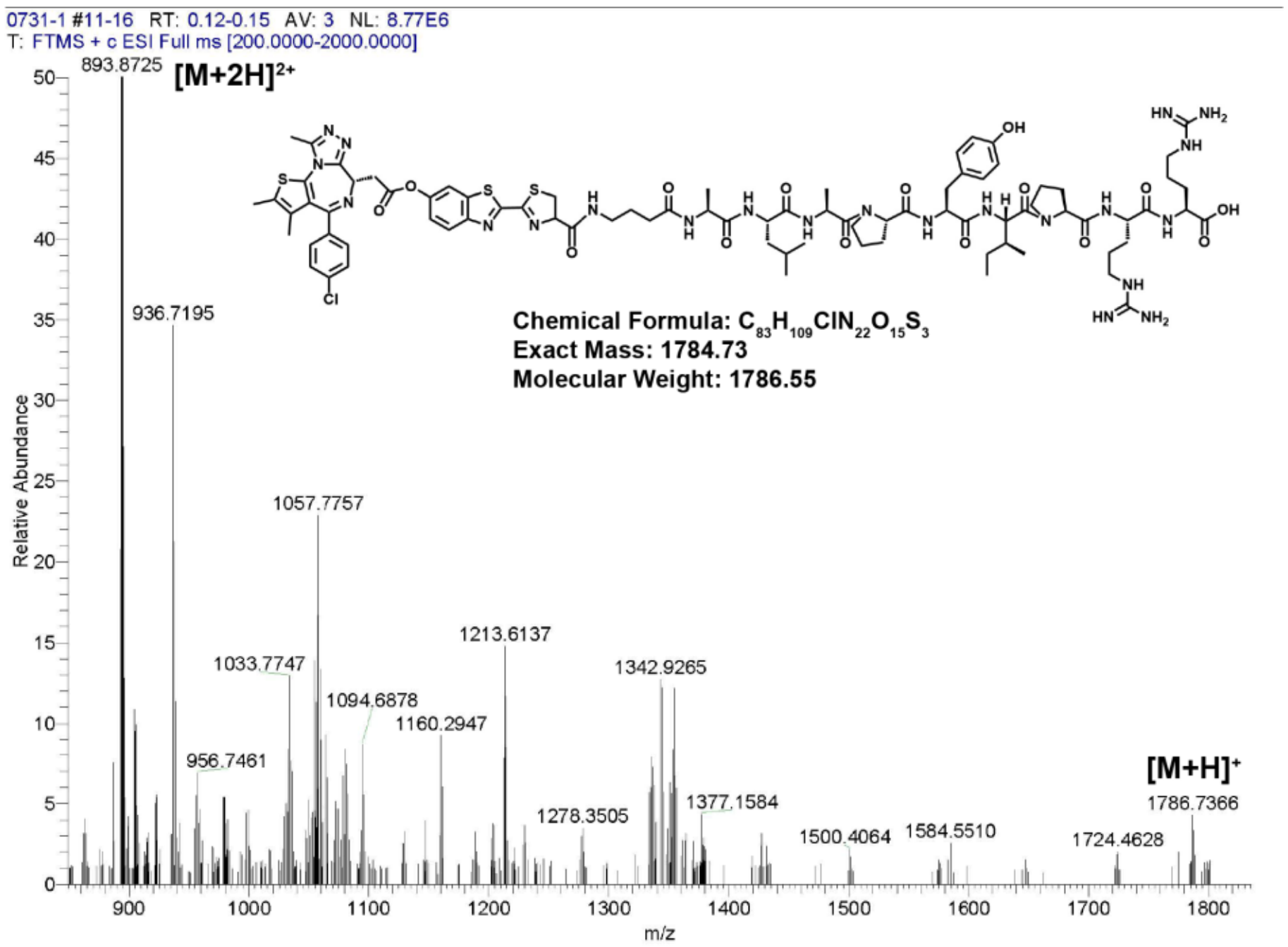

Figure S19. HRMS analysis of intracellular PROTAC formation in 4T1 cells. 4T1 cells were incubated with SNAP-TAC (30 µM) for 12 h at 37 °C. The cells were harvested, lysed, and extracted with an 80% (v/v) aqueous acetonitrile solution to precipitate cellular proteins and enrich intracellular small molecules. The resulting supernatant was analyzed by HRMS. HRMS (ESI) m/z: Calcd. for the M+1 isotopic peaks of $C_{83}H_{109}ClN_{22}O_{15}S_3$ $[M+H]^+$ 1786.7397 and $[M+2H]^{2+}$ 893.8735; found 1786.7366 $[M+H]^+$ and 893.8725 $[M+2H]^{2+}$, corresponding to the *in situ* assembled PROTAC.

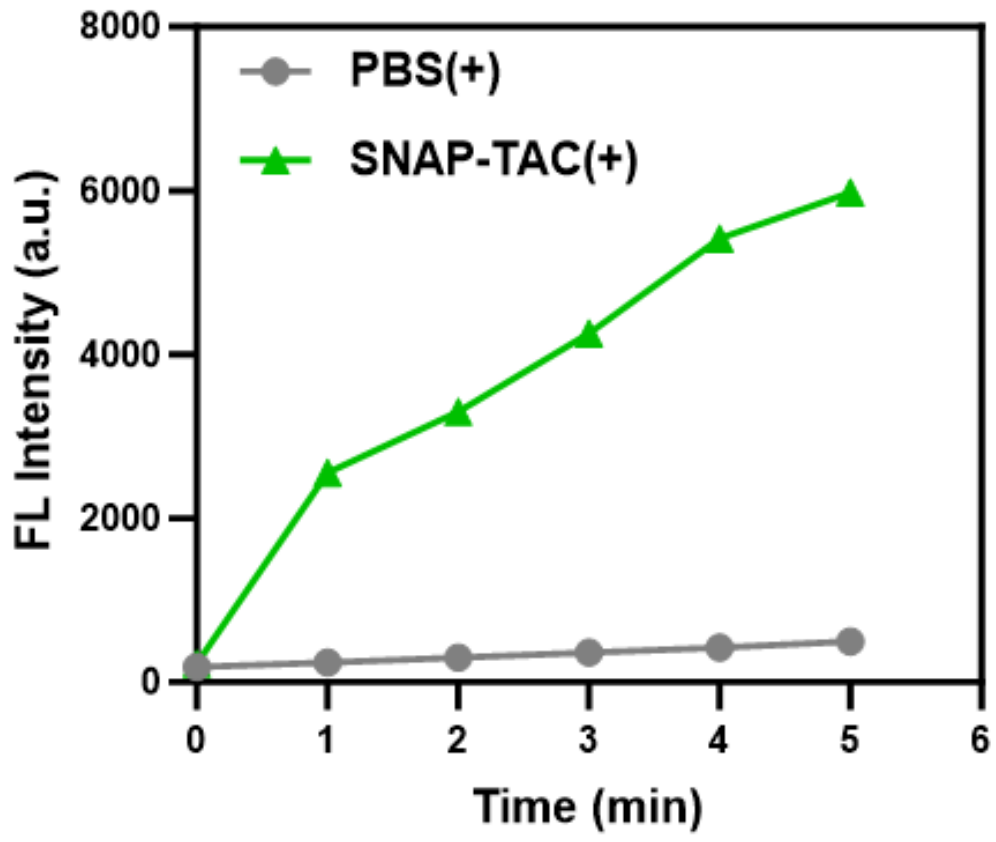

Figure S20. Time-dependent curves of reactive oxygen species (ROS) generation by PBS and SNAP-TAC under light irradiation (+), monitored via DCFH-DA fluorescence.

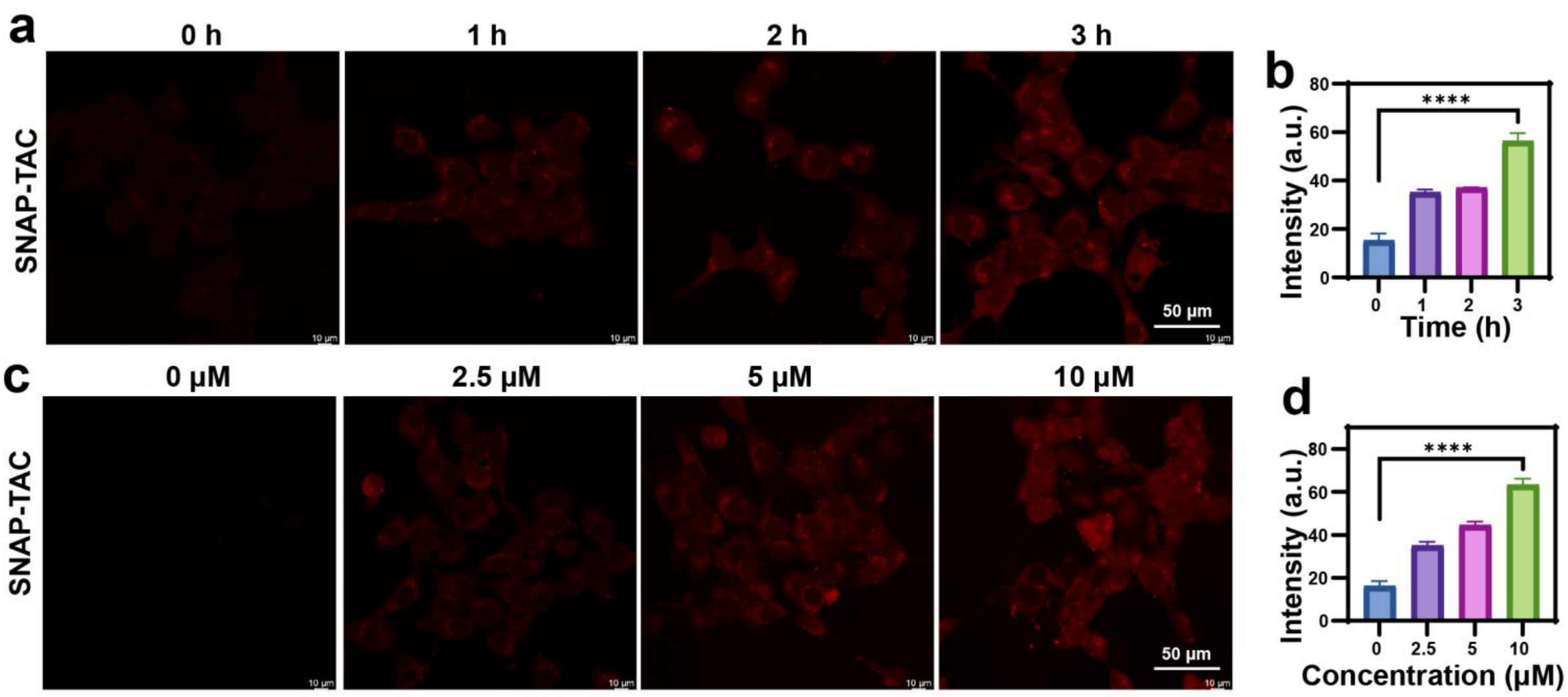

Figure S21. Cellular uptake of SNAP-TAC in 4T1 cells. Time-dependent (a) CLSM images and (b) fluorescence quantification (SNAP-TAC: 10 μM). Concentration-dependent (c) CLSM images and (d) fluorescence quantification (Incubation time: 3 h). Scale bars = 50 μm. Data are mean ± SD (n = 3). P values were determined by One-way ANOVA with Tukey's post hoc test. ****$P < 0.0001$.

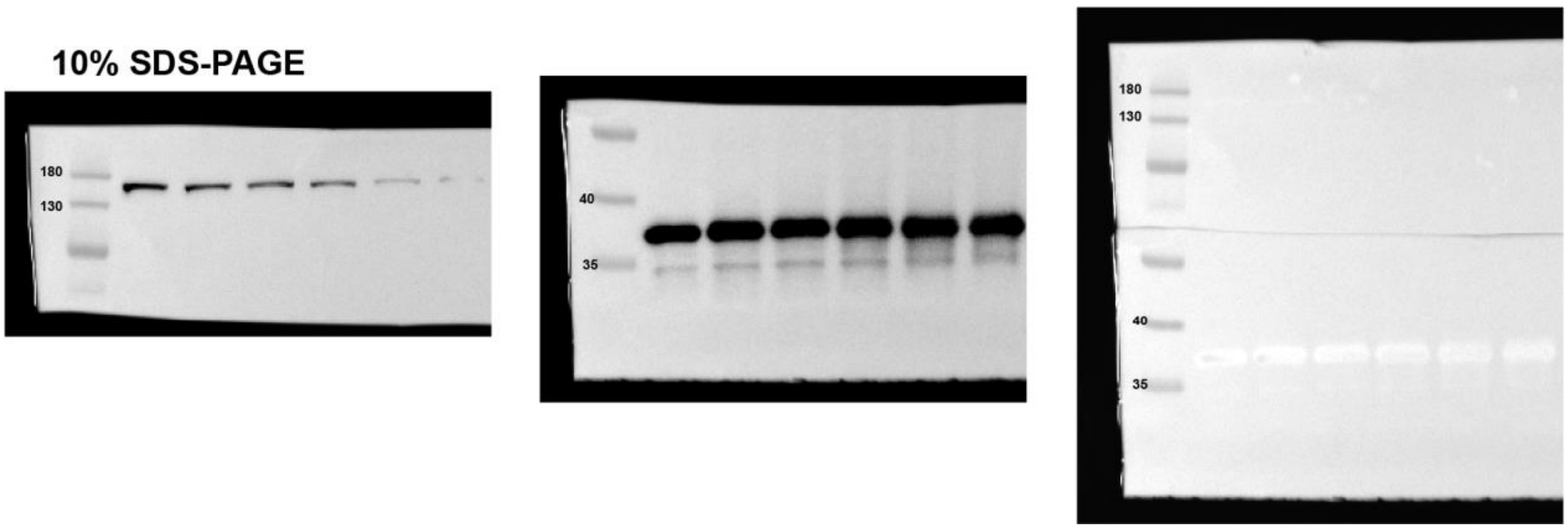

Figure S22. Concentration-dependent targeted degradation of BRD4 by SNAP-TAC in 4T1 cells. Western blot analysis of BRD4 and GAPDH protein levels after incubation with various concentrations of SNAP-TAC (0, 0.625, 1.25, 2.5, 5, 10 μM) for 48 h. GAPDH was used as the loading control. The corresponding overlay images and their white light photographs are provided.

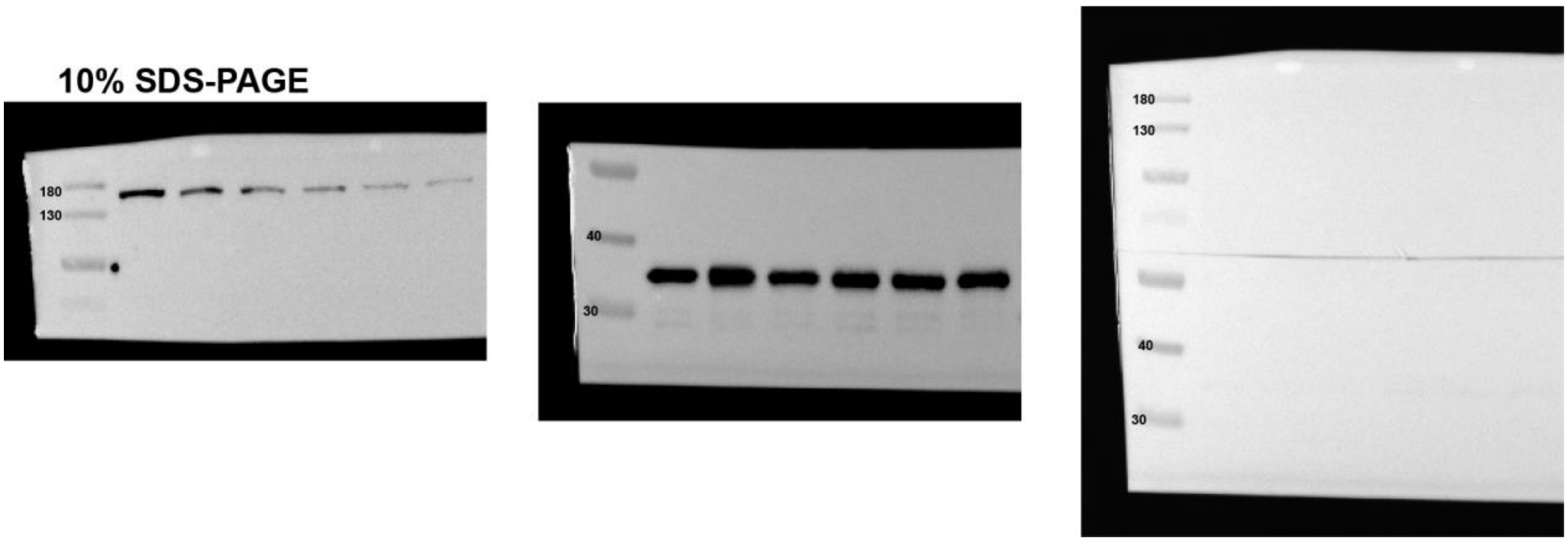

Figure S23. Time-dependent targeted degradation of BRD4 by SNAP-TAC in 4T1 cells. Western blot analysis of BRD4 and GAPDH protein levels after incubation with SNAP-TAC (5 μM) for various times (0, 6, 12, 24, 36, 48 h). GAPDH was used as the loading control. The corresponding overlay images and their white light photographs are provided.

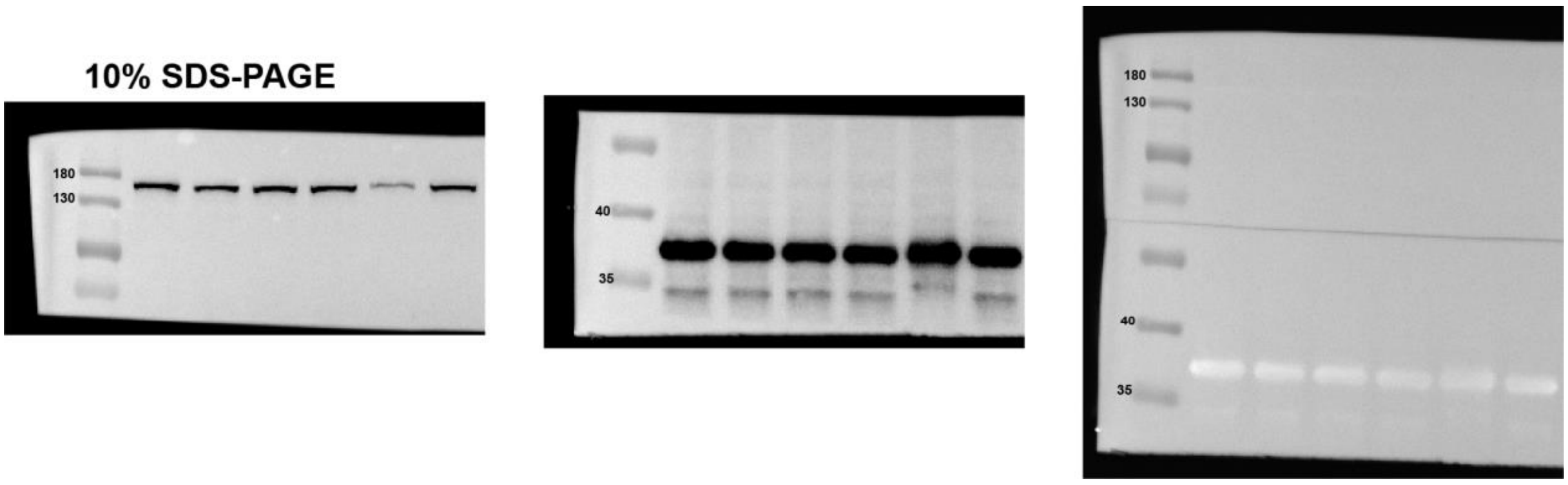

Figure S24. Mechanistic investigation of BRD4 degradation by SNAP-TAC in 4T1 cells. Western blot analysis of BRD4 and GAPDH protein levels after various control treatments (PBS, Pep-PpIX+JQ1, JQ1-CBT, Pep-PpIX, SNAP-TAC, SNAP-TAC + MG132) for 48 h. GAPDH was used as the loading control. The corresponding overlay images and their white light photographs are provided.

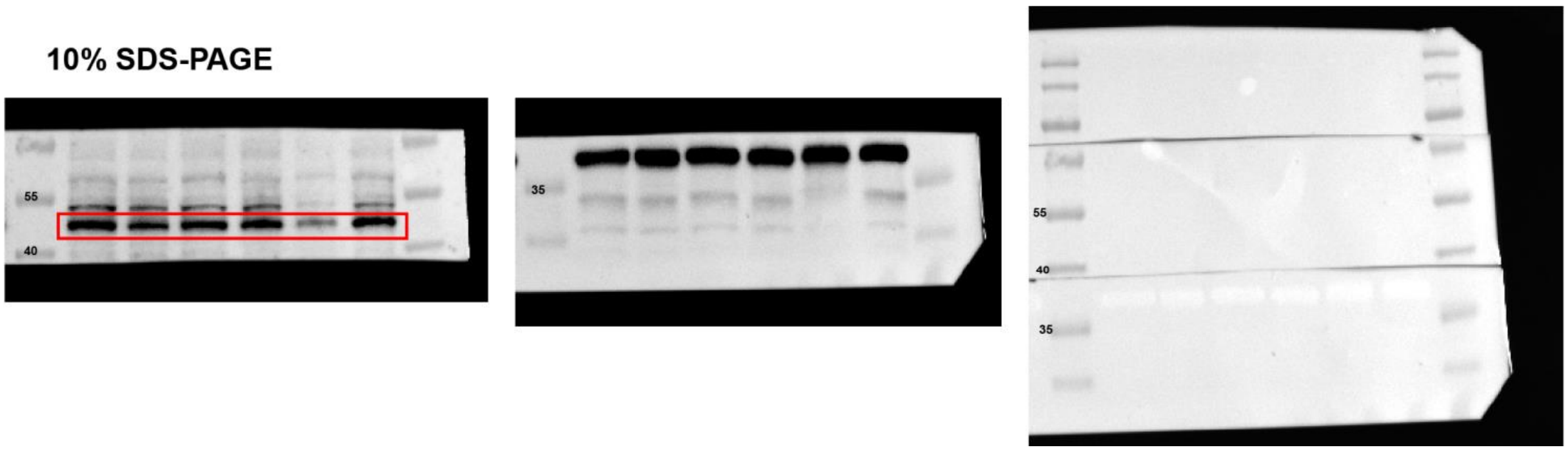

Figure S25. Downregulation of PD-L1 expression by SNAP-TAC in 4T1 cells. Western blot analysis of PD-L1 and GAPDH protein levels after various treatments (PBS, Pep-PpIX+JQ1, JQ1-CBT, Pep-PpIX, SNAP-TAC, SNAP-TAC + MG132) for 48 h. GAPDH was used as the loading control. The corresponding overlay images and their white light photographs are provided.

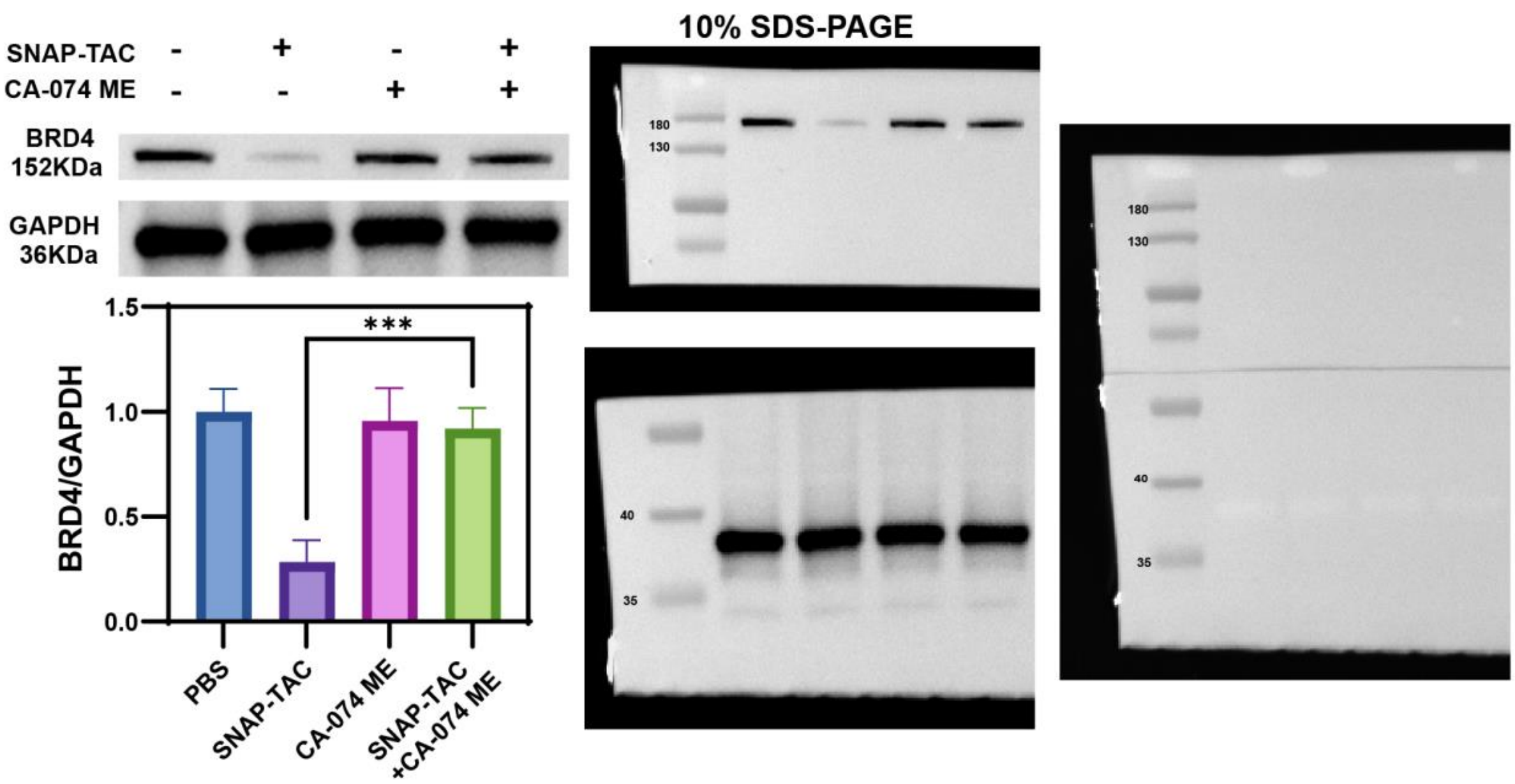

Figure S26. Rescue of SNAP-TAC-induced BRD4 degradation by the CTSB inhibitor CA-074 ME. Western blot analysis and corresponding quantification of BRD4 and GAPDH protein levels in 4T1 cells treated with PBS, SNAP-TAC alone, CA-074 ME alone, or their combination. GAPDH was used as the loading control. The corresponding overlay images and their white light photographs are provided on the right. Data are presented as mean ± SD (n = 3). P values were determined by One-way ANOVA with Tukey's post hoc test. ***P < 0.001.

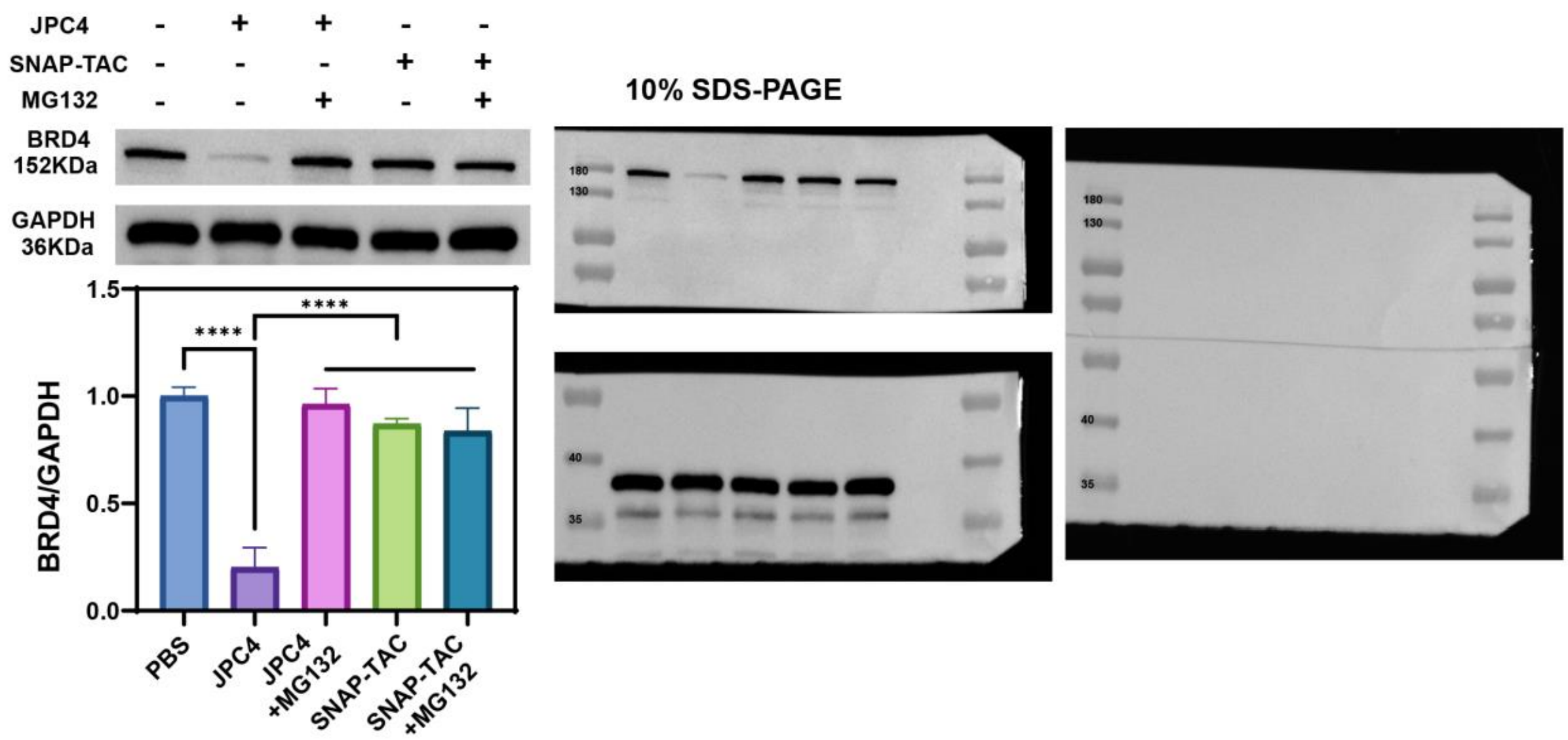

Figure S27. Evaluation of SNAP-TAC activation and BRD4 degradation in normal NIH-3T3 cells. Western blot analysis and corresponding quantification of BRD4 and GAPDH protein levels in normal NIH-3T3 fibroblasts treated with PBS, JPC4 (a pre-synthesized active PROTAC), JPC4 + MG132, SNAP-TAC, or SNAP-TAC + MG132. GAPDH was used as the loading control. The corresponding overlay images and their white light photographs are provided on the right. Data are presented as mean ± SD (*n* = 3). *P* values were determined by one-way ANOVA with Tukey's post-hoc test. *****P* < 0.0001.

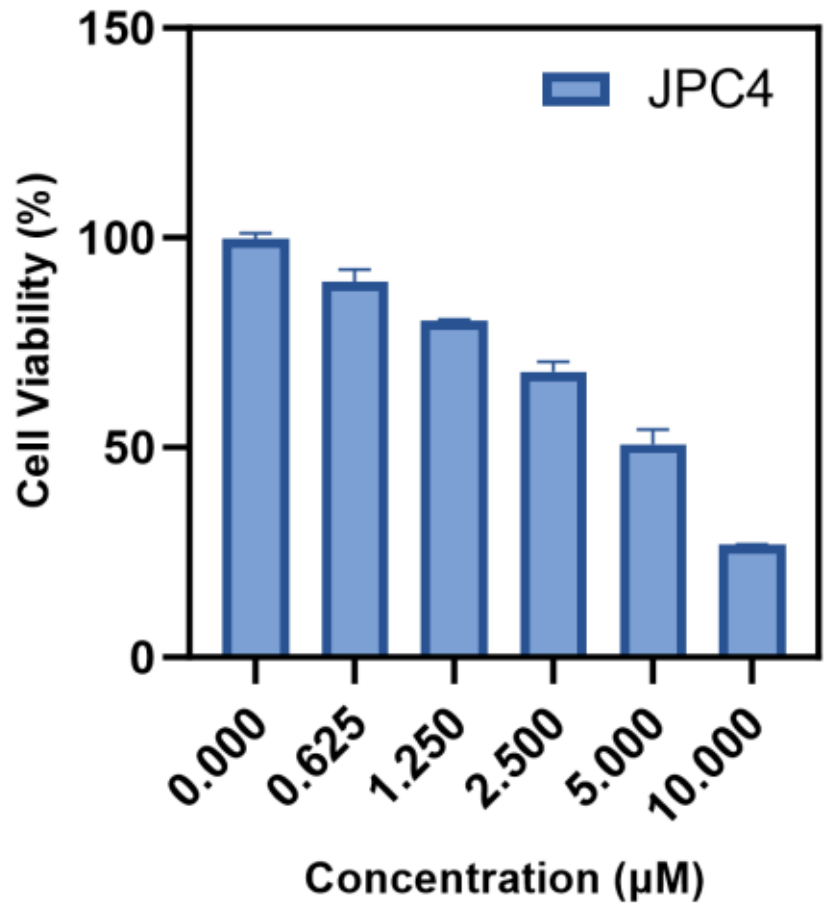

Figure S28. Cell viability of NIH-3T3 normal fibroblasts treated with indicated concentrations of intact JPC4 for 48 h. Data are presented as mean ± SD (*n* = 3).

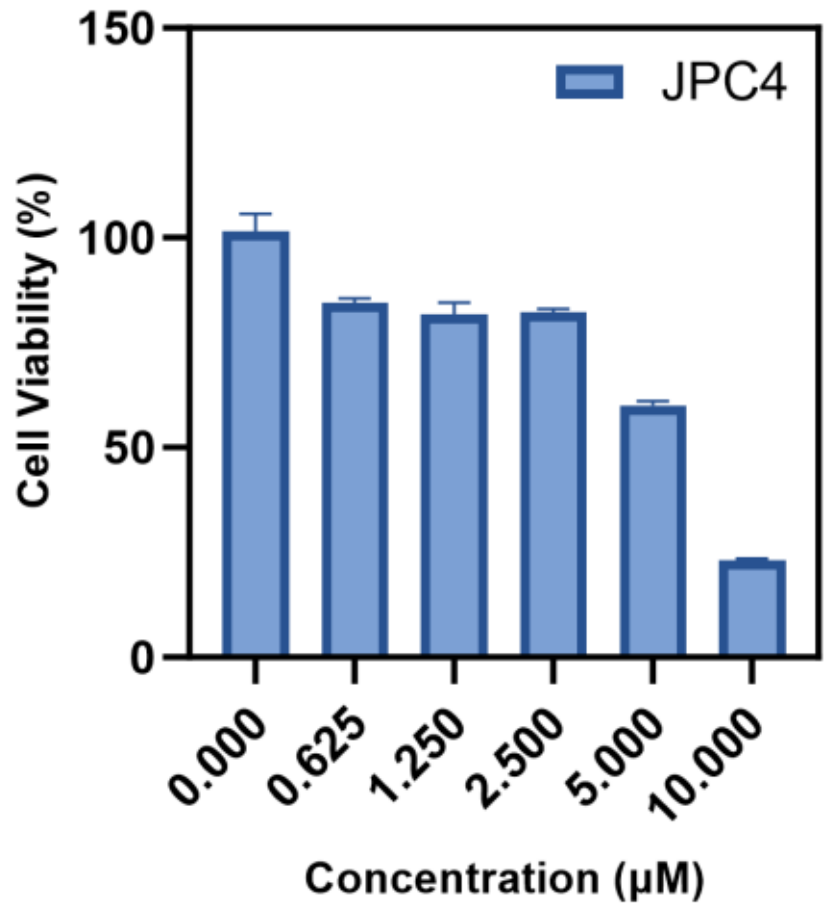

Figure S29. Cell viability of 4T1 breast cancer cells after treatment with indicated concentrations of intact JPC4 for 48 h. Data are presented as mean ± SD (n = 3).

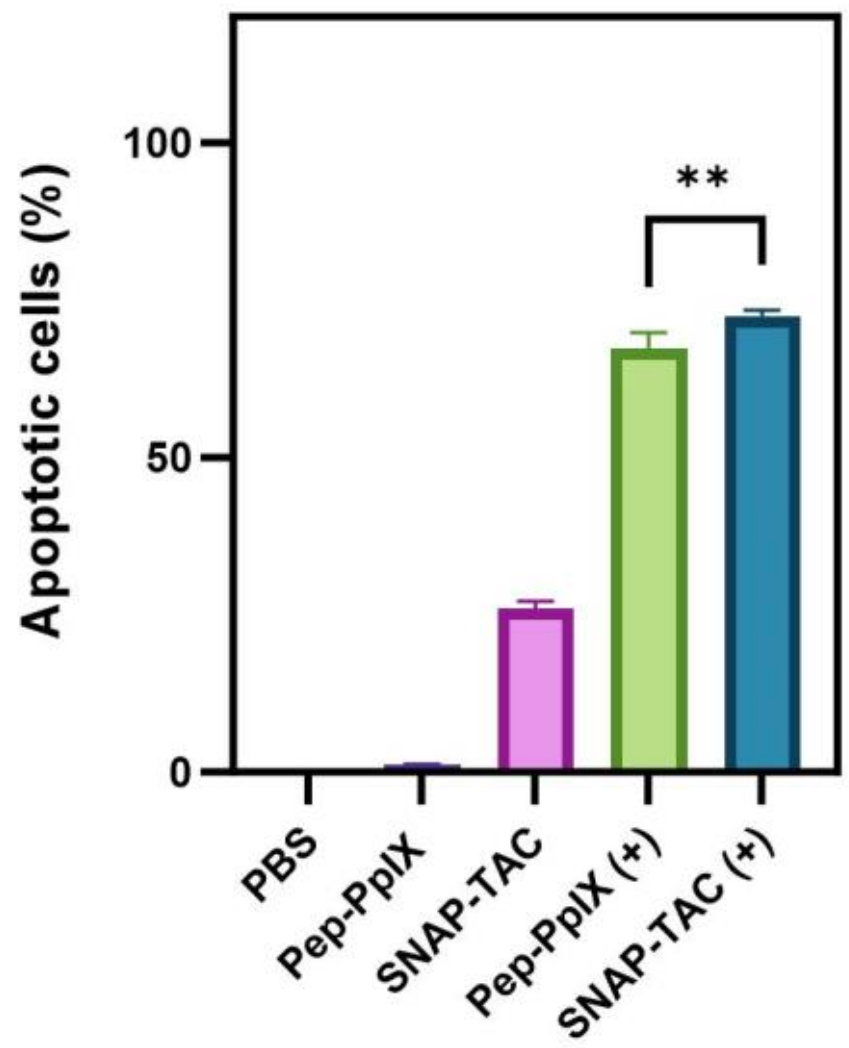

Figure S30. Quantitative analysis of apoptotic cell percentages (including early and late apoptosis) in 4T1 cells treated with different formulations for 48 h. Data are presented as mean ± SD (n = 3). P values were determined by one-way ANOVA with Tukey's post-hoc test. **P < 0.01.

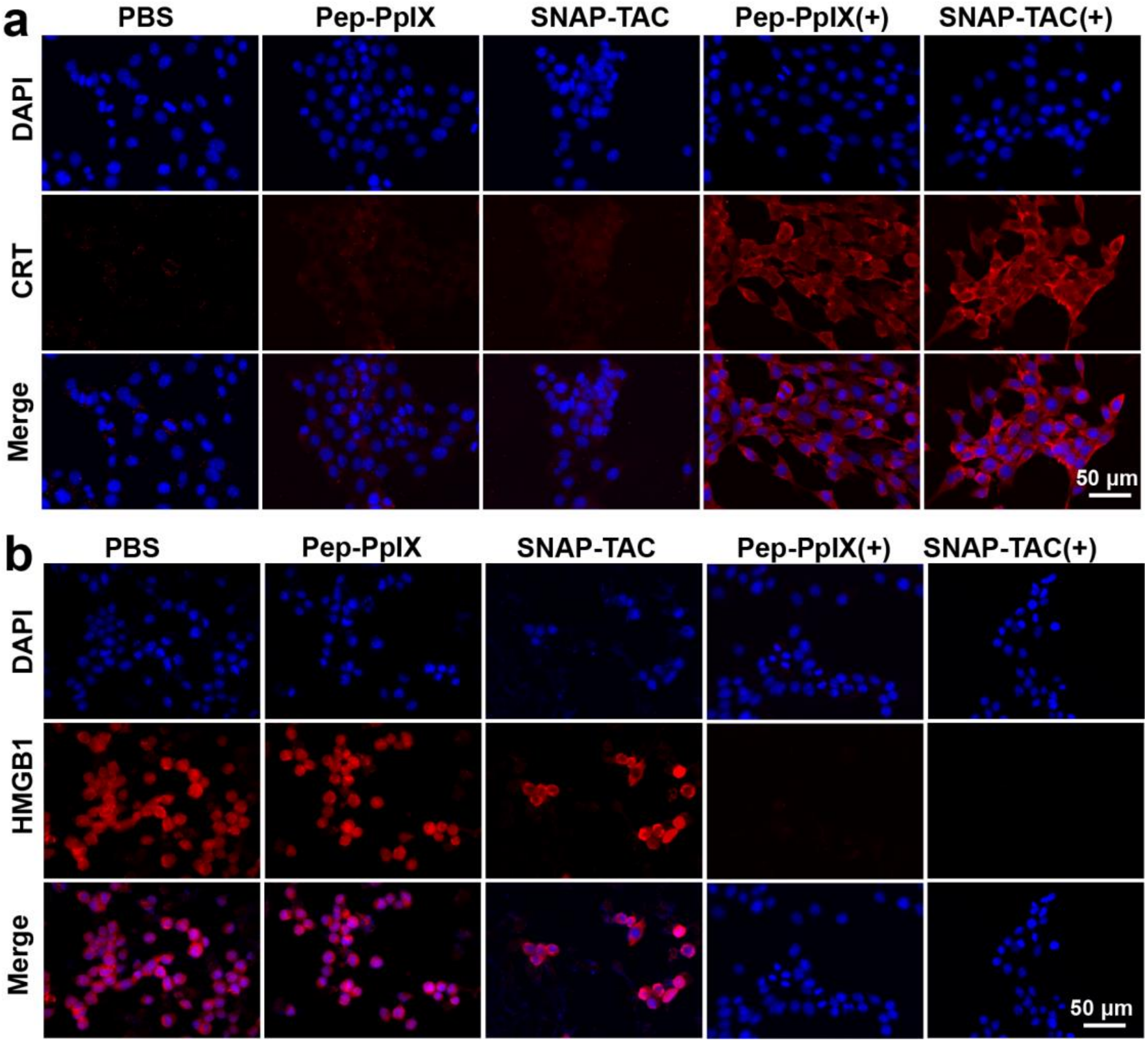

Figure S31. Immunofluorescence imaging of ICD markers in 4T1 cells. Individual channels and merged images of (a) CRT and (b) HMGB1 expression after various treatments. Scale bars = 50 μm.

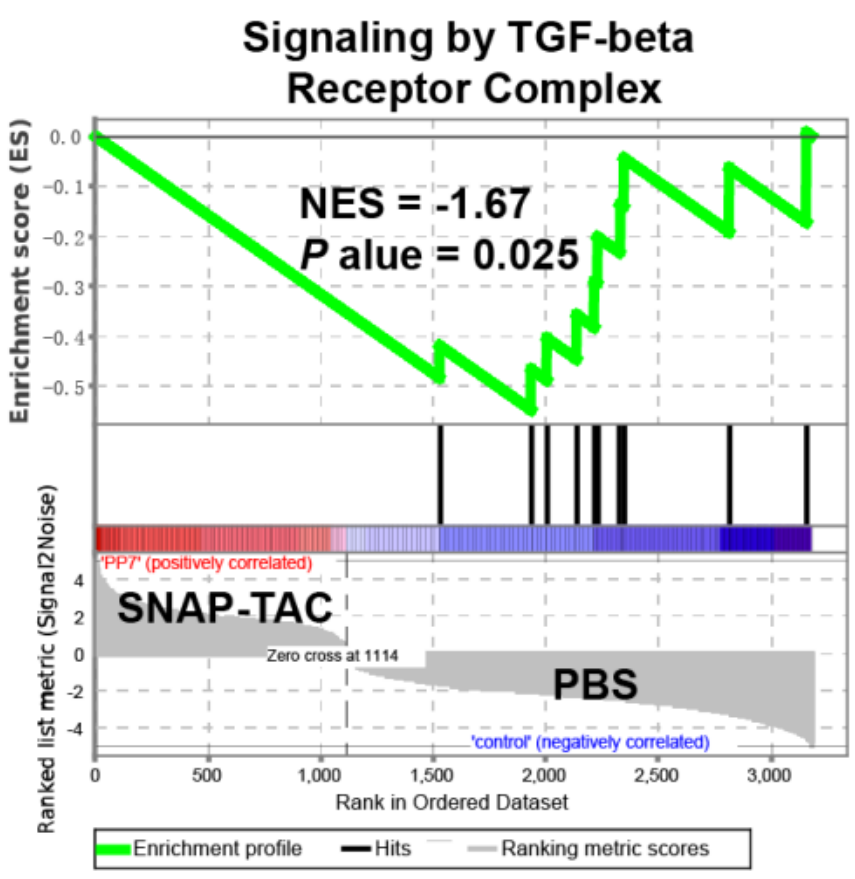

Figure S32. Downregulation of TGF- signaling by SNAP-TAC. Gene Set Enrichment Analysis (GSEA) plot showing the significant negative enrichment of the signaling by TGF-β receptor complex pathway in 4T1 cells with SNAP-TAC compared to the PBS group.

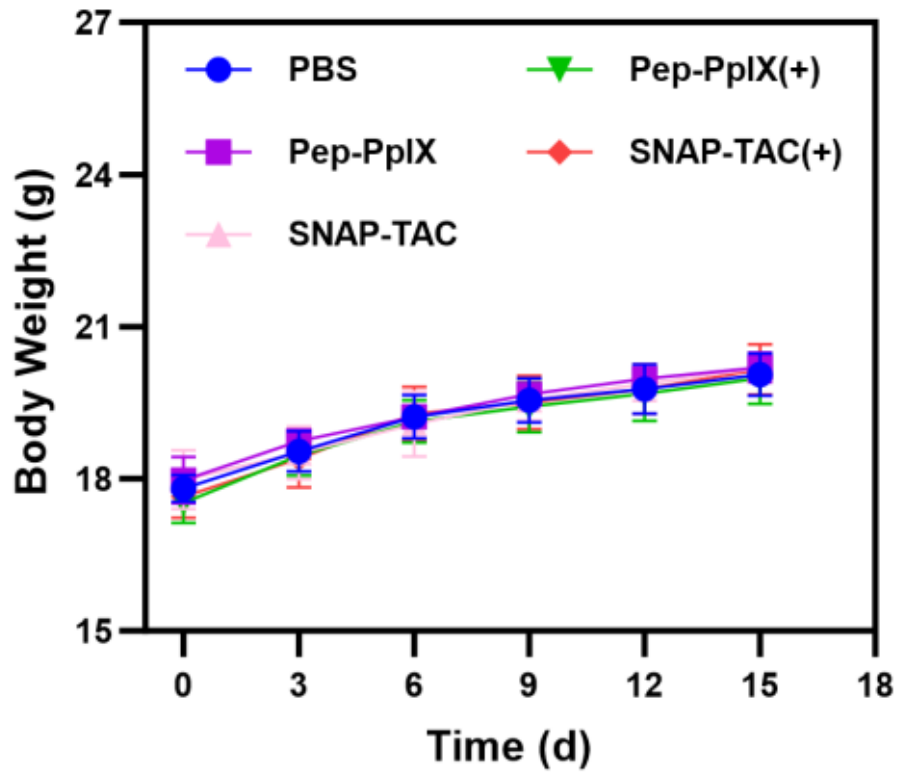

Figure S33. Body weight changes of 4T1 tumor-bearing mice in different treatment groups during the 15-day therapeutic period. Data are presented as mean ± SD (n = 5).

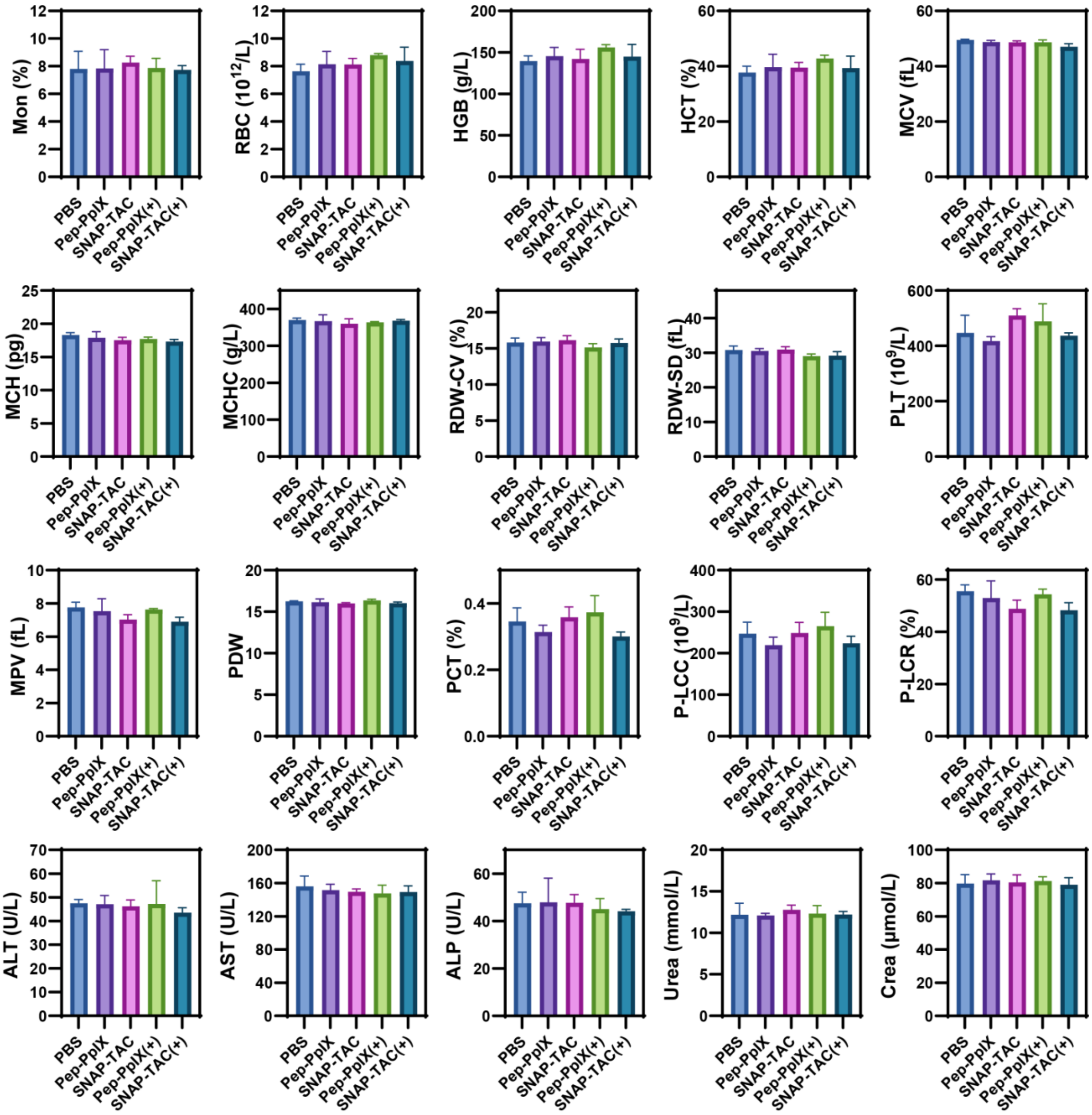

Figure S34. Hematology and serum biochemistry analysis of mice from different treatment groups at the end of the therapeutic period. Data are presented as mean ± SD (n = 3).

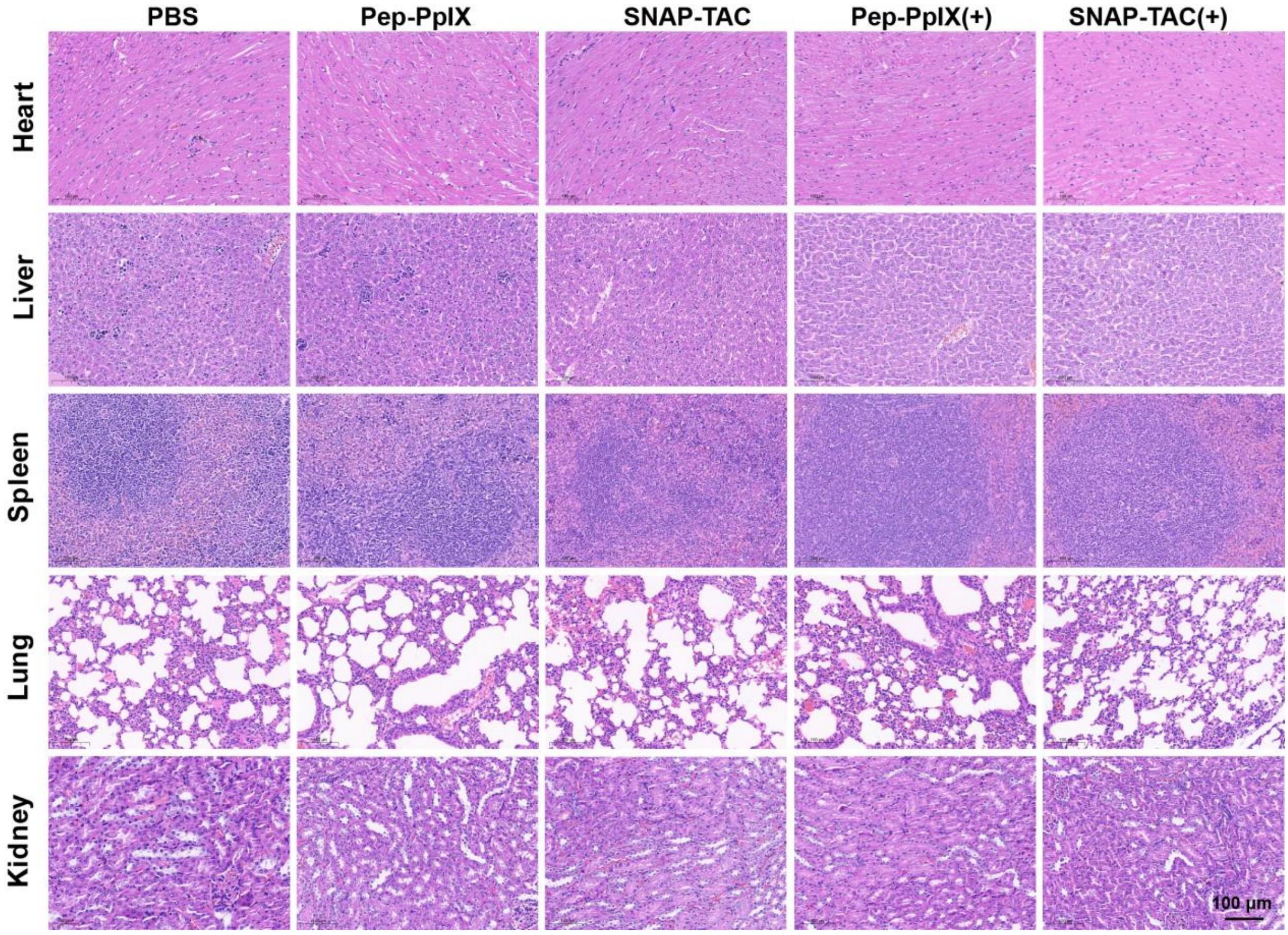

Figure S35. Representative H&E stained images of major organs (heart, liver, spleen, lung, and kidney) collected from mice in different treatment groups. Scale bars = 100 μm.

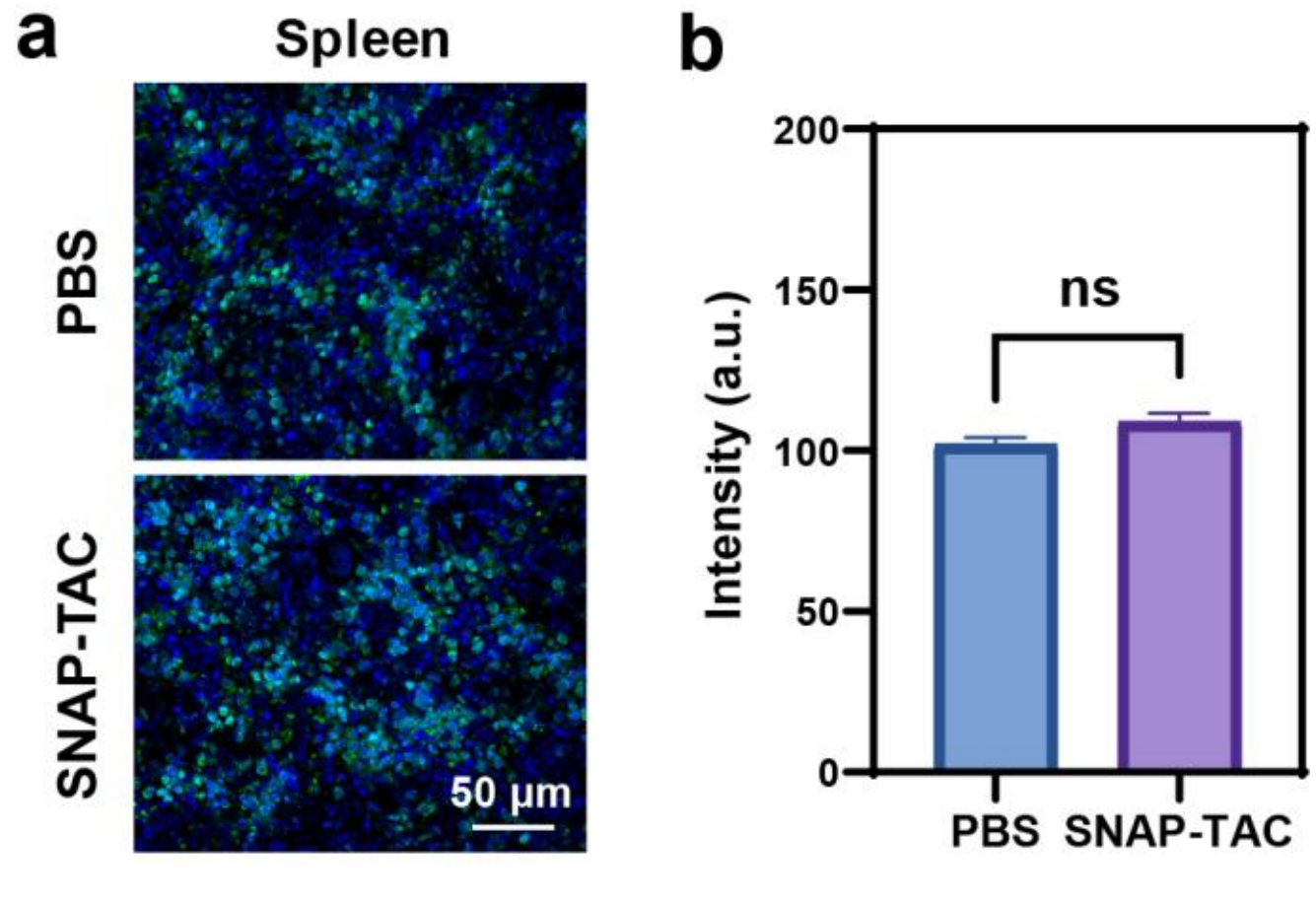

Figure S36. Evaluation of BRD4 protein levels in the spleen. (a) Representative BRD4 immunofluorescence images of the spleen collected from mice in the PBS and SNAP-TAC groups. Scale bars = 50 μm. (b) Quantitative analysis of BRD4 immunofluorescence in the spleen. Mean fluorescence intensity (a.u.) of BRD4 in the spleen sections from the PBS and SNAP-TAC groups. Data are presented as mean ± SD (n = 3). P values were determined by a two-tailed unpaired Student's t-test. ns: not significant.

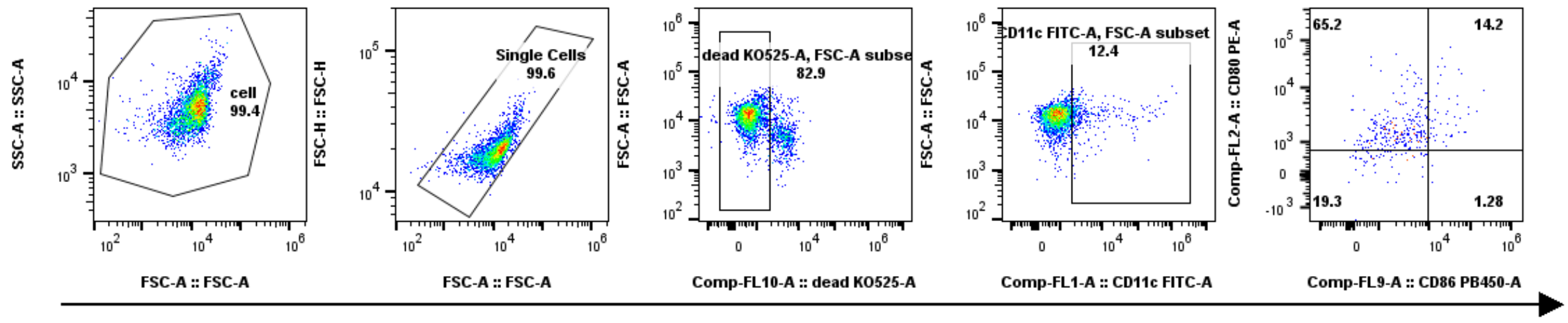

Figure S37. Flow cytometry gating strategy for mature dendritic cells (DCs) in tumor-draining lymph nodes. Briefly, after excluding debris and dead cells, the total DC population was identified by CD11c^{+} expression. Mature DCs were subsequently defined as the CD80^{+} CD86^{+} double-positive subpopulation.

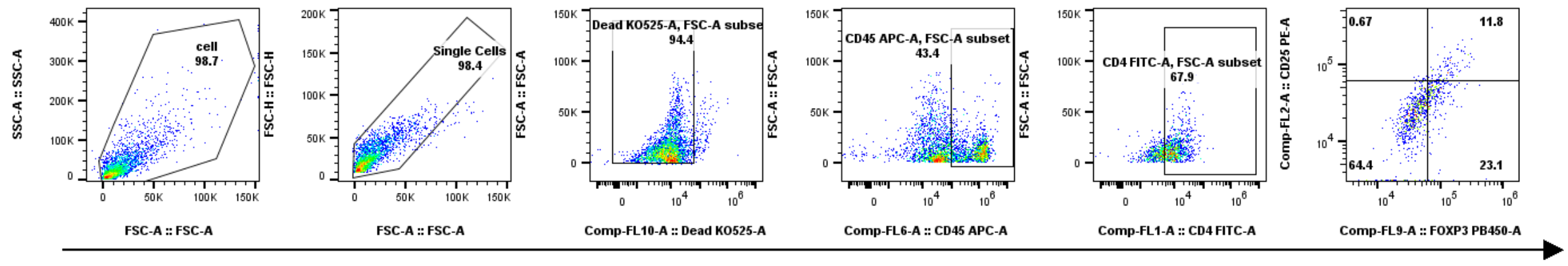

Figure S38. Flow cytometry gating strategy for regulatory T cells (Tregs) in tumor tissues. Briefly, cells were sequentially gated for live cells and leukocytes (CD45^{+}), followed by the identification of CD4^{+} T cells. Tregs were subsequently defined as the CD25^{+} FOXP3^{+} subpopulation.cc

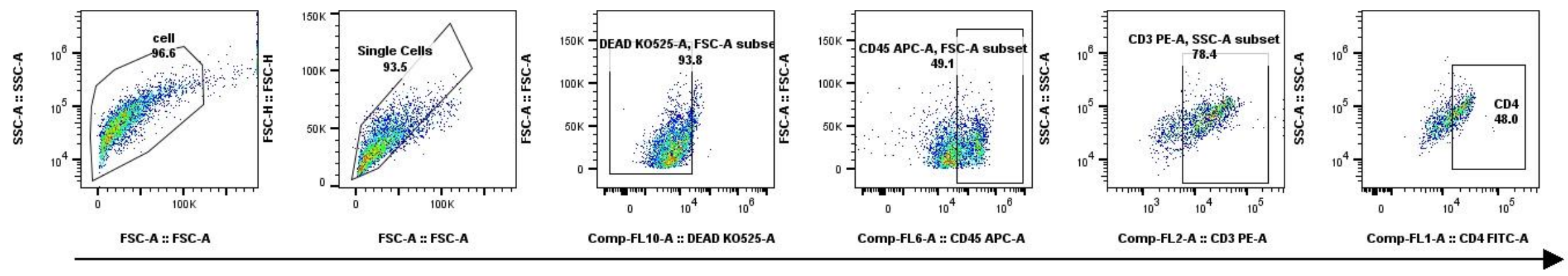

Figure S39. Flow cytometry gating strategy for CD4^{+} T cells in tumor tissues. Briefly, cells were sequentially gated for live cells, leukocytes (CD45^{+}), and T cells (CD3^{+}) before the final identification of the CD4^{+} T cell subset.

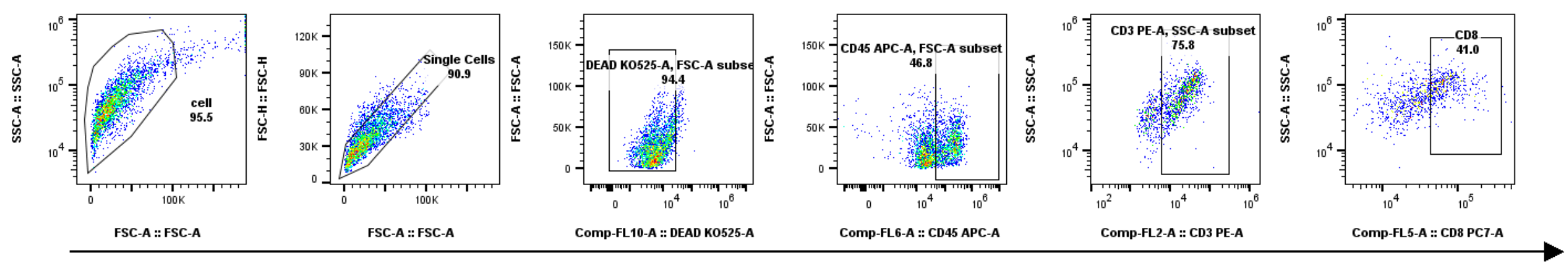

Figure S40. Gating strategy for the identification of tumor-infiltrating CD8^{+} T cells. Briefly, cells were sequentially gated for live cells, leukocytes (CD45^{+}), and T cells (CD3^{+}) before the final identification of the CD8^{+} T cell subset.

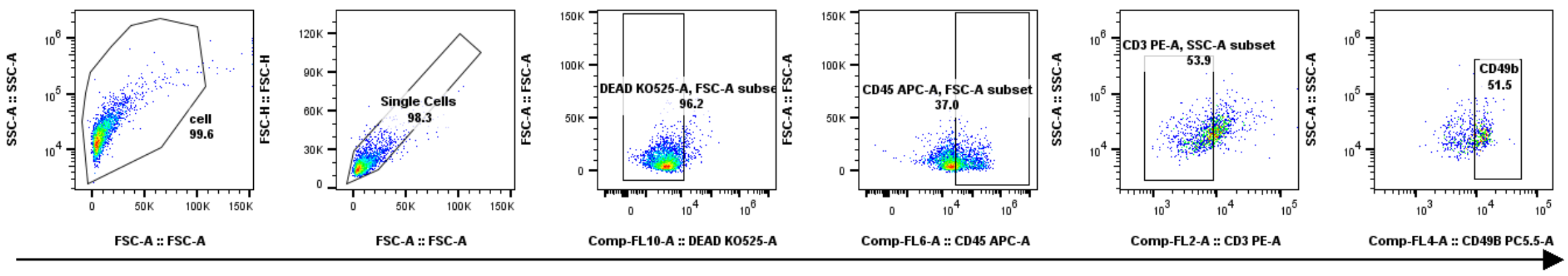

Figure S41. Flow cytometry gating strategy for NK cells in tumor tissues. Briefly, cells were sequentially gated for live cells, leukocytes ($CD45^{+}$), and non-T cells ($CD3^{-}$) before the final identification of the $CD49b^{+}$ NK cell subset.
